# Structure-guided design of picomolar-level macrocyclic TRPC5 channel inhibitors with antidepressant activity

**DOI:** 10.1016/j.apsb.2025.10.028

**Published:** 2025-10-27

**Authors:** Tong Che, Yixiang Chen, Xinyu Cheng, Han Hu, Xiaoyun Wu, Yuting Zhang, Xiaoqiang Yang, Yinzhen Liu, Hui Liu, Weiwei Nan, Shuangyan Wan, Mingxing Yang, Bo Zeng, Jian Li, Jin Zhang, Bing Xiong

**Affiliations:** a The MOE Basic Research and Innovation Center for the Targeted Therapeutics of Solid Tumors, School of Basic Medical Sciences, Jiangxi Medical College, Nanchang University, Nanchang 330031, China; b The Second Affiliated Hospital, Jiangxi Medical College, Nanchang University, Nanchang 330031, China; c Shenzhen Crystalo Biopharmaceutical Co., Ltd., Shenzhen 518118, China; d Key Laboratory of Medical Electrophysiology, Ministry of Education and Sichuan Province and Institute of Cardiovascular Research, Southwest Medical University, Luzhou 646000, China; e Department of Endocrinology, Affiliated Hospital of Southwest Medical University, Luzhou 646000, China; f College of Pharmacy, Gannan Medical University, Ganzhou 341000, China; g Department of Medicinal Chemistry, Shanghai Institute of Materia Medica, Chinese Academy of Sciences, Shanghai 201203, China

**Keywords:** TRPC5, Ion channel, Structure-based drug design, Macrocyclization, Selectivity, Cryo-EM, Antidepressant, Anxiolytic

## Abstract

Recent advances in ion channel structural biology have enhanced structure-based drug design, yet lipid-occupied binding pockets—often large and flat—remain a major hurdle for developing selective small molecules. TRPC5, a brain-enriched channel regulating depression and anxiety, is a promising therapeutic target, but current preclinical candidates suffer from moderate off-target effects. To address this, we designed macrocyclic TRPC5 inhibitors using structure-guided macrocyclization, overcoming lipid-binding site challenges. Among these, JDIC-127 exhibited unprecedented potency with IC_50_ of 374 pmol/L—200-fold more potent than HC-070—and exceptional selectivity. Its specificity arises from interactions with unique structural features near the S5 and S6 helices of TRPC5, minimizing activity against related TRPC channels and other ion channels. This selective inhibition aligns with preclinical evidence supporting JDIC-127's potential in treating neuropsychiatric disorders. The study demonstrates how macrocycles stabilize ligand conformations, enhance affinity, and achieve selectivity in lipid-dominated binding sites. It also highlights the synergy between macrocyclic design, cryo-EM, and computational modeling to address longstanding obstacles in ion channel drug discovery. JDIC-127 serves as a proof-of-concept for the application of macrocyclization in ion channel pharmacology, offering a roadmap for developing innovative therapeutics targeting TRP channels and beyond, with implications for a wide range of diseases.

## Graphical abstract

The macrocyclic compound JDIC-127 has demonstrated exceptional potency towards TRPC5, with an IC_50_ of 374 pmol/L, which is 200-fold more potent than HC-070, and exhibits significant selectivity against other TRP channels.

**Figure:**
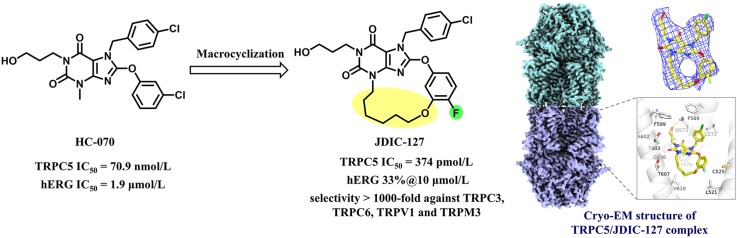

## 1 Introduction

Ion channels, which regulate the flow of ions between intracellular and extracellular environments, play a critical role in nearly all aspects of physiology. Their dysfunction is associated with a wide range of diseases, making ion-channel-targeted drug discovery highly compelling^1^, ^2^, ^3^, ^4^. However, despite the availability of some important drugs in clinical use today, most first-generation ion channel drugs are relatively nonselective, often exhibiting dose-limiting side effects and suboptimal efficacy^5^. With the rapid advancements in structural biology over the past two decades, an increasing number of ion channel structures have been reported^6^, ^7^, ^8^, ^9^. Despite this progress, the discovery of ion channel-targeting drugs remains a formidable challenge^4^^,^^5^^,^^10^. There have been few successes in structure-guided drug design for ion channels^11^, primarily due to the large, flat, and often lipid-exposed nature of their binding pockets.

How can we design potent and selective molecules by leveraging the combination of flat and large binding pockets? Macrocycles offer a promising solution. Macrocycles, which contain a ring of at least 12 heavy atoms, have emerged as a promising strategy in drug discovery^12^, ^13^, ^14^, ^15^. Compared to their linear analogs, macrocycles tend to adopt preorganized, constrained conformations that facilitate extended interactions with target molecules^16^^,^^17^. As a result, they often exhibit enhanced binding affinities, improved selectivity, and superior pharmacological properties^17^, ^18^, ^19^. A range of synthetic macrocycles has been successfully developed as potential therapeutic agents for various pharmaceutical targets, such as kinases^20^^,^^21^, proteases^22^^,^^23^ and G-protein-coupled receptors (GPCRs)^24^^,^^25^. However, few structure-based macrocyclization studies have been conducted on ion channels.

The TRPC5 channel functions as a non-selective, calcium-permeable cation channel that plays a crucial role in transducing sensory stimuli into electrical signals in the human brain^26^^,^^27^. It is primarily activated *via* the G*α*_q_–PLC-coupled pathway and regulates neuronal excitability through integration of signals from GPCRs^28^^,^^29^. Studies in murine models have linked TRPC5 activation to fear-related behaviors and anxiety^28^. Preclinical research has shown that either genetic deletion of TRPC5 or its pharmacological inhibition significantly reduces fear and anxiety behaviors in mice without impairing other behavioral functions^30^^,^^31^. These findings underscore the therapeutic potential of TRPC5 inhibitors as promising candidates for the treatment of anxiety and depression.

**Figure 1:**
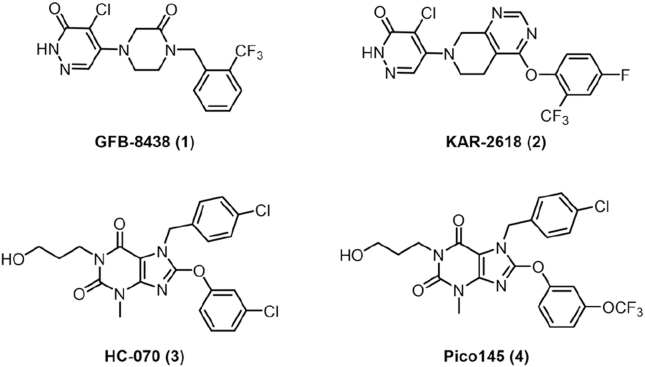
Chemical structures of representative TRPC5 inhibitors (1–4).

Several small molecules have been identified as potent inhibitors of TRPC5^32^^,^^33^, including GFB-8438 (**1**)^34^, KAR-2618 (known as GFB-887, **2**)^35^, HC-070 (**3**)^30^ and Pico145 (**4**)^31^ (Fig. 1). BI 1358894, a small-molecule inhibitor of TRPC5, is currently being developed for clinical use in the treatment of post-traumatic stress disorder (PTSD) and major depressive disorder (MDD)^36^, ^37^, ^38^. Recently, cryo-electron microscopy (cryo-EM) structures of TRPC5 were determined in both apo and ligand-bound states^39^, ^40^, ^41^. Among these studies, one identified the binding site of HC-070, located near the pore helix, at the interface between the S5 helix of one subunit and the S6 helix of the adjacent subunit^41^. HC-070 binds at the protein-lipid interface, where its flexible phenyl ring adopts a cloverleaf-like conformation, leaving an open pocket. This structural insight suggests the potential for designing macrocyclic molecules inspired by HC-070.

Here, we designed and synthesized a series of novel macrocyclic TRPC5 inhibitors by stabilizing the conformations through macrocyclization. The conformation of the macrocyclic compound was further validated by cryo-EM. Additionally, we evaluated the antidepressant and anxiolytic activities of the macrocyclic compounds *in vivo*. Overall, we present macrocyclization as a powerful strategy for structure-guided drug design, enabling the development of novel ion channel ligands.

## 2 Results and discussion

### 2.1 Molecular design of macrocyclic TRPC5 inhibitors

**Figure 2:**
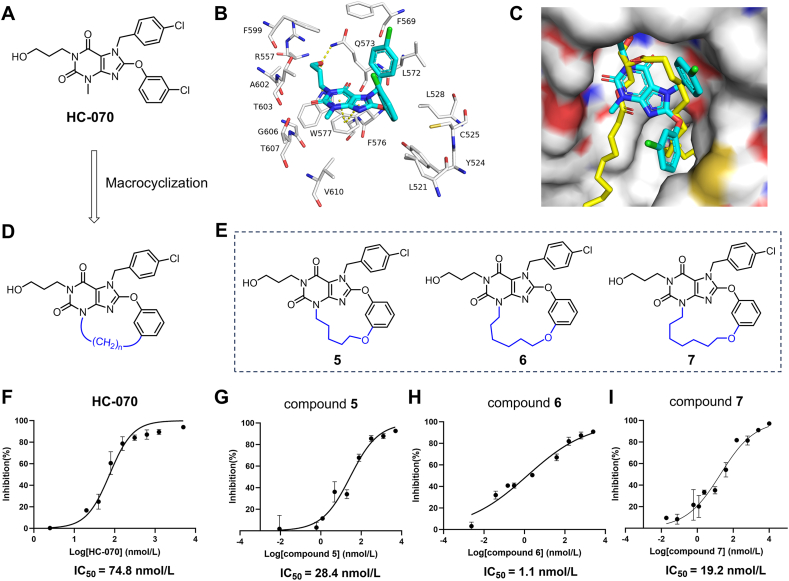
Macrocyclic TRPC5 inhibitors design strategy. (A) The chemical structure of HC-070. (B) The interactions between HC-070 and TRPC5. HC-070 and TRPC5 are shown as sticks, colored in cyan and white, respectively. (C) Molecular surface representation of HC-070 binding pocket. HC-070 and phospholipid are shown as sticks, colored in cyan and yellow, respectively. (D) The design strategy of macrocyclization. (E) Chemical structures of macrocyclic. (F–I) Inhibitory effect of HC-070 (F), compound **5** (G), compound **6** (H) and compound **7** (I) on TRPC5 measured by calcium flux assay (Data are shown as means ± SEM, *n* = 3 independent experiments).

We developed a strategy for generating macrocycle-based TRPC5 inhibitors, informed by the cryo-EM structure of TRPC5 in complex with HC-070 (Fig. 2A)^41^. HC-070 binds at the portal site, formed by the S5 helix, pore helix from one subunit, and S6 from the adjacent subunit, a region typically occupied by phospholipid in the apo state (Fig. 2B and C). The methylxanthine core of HC-070 forms *π*–*π* stacking interactions with F576 on the pore helix, while its hydroxypropyl tail forms hydrogen bonds with Q573 and W577 on the pore helix (Fig. 2B). The flexible chlorophenyl ring of HC-070 is exposed to a hydrophobic cavity and interacts with F569 and L572 on S5. Overall, HC-070 adopts a clover-shaped conformation, binding within a wide, shallow, lipid-exposed lipophilic cavity.

Inspired by these features, we hypothesized that macrocyclization of the HC-070 structure through connecting the benzyl and methylxanthine core with non-polar linker could restrict the conformation to specifically fit the pocket of TRPC5 and enhance the interaction with lipophilic pocket, thus affording more potency and selectivity (Fig. 2D). Therefore, we synthesized a series of macrocyclic derivatives with diverse ring sizes and characterized their inhibitory activity against TRPC5. The 14- to 16-membered ring macrocyclic compounds **5**–**7** were successfully synthesized (Fig. 2E). In TRPC5-stably transfected HEK293T cells, we assessed the inhibitory activity of macrocyclic compounds against TRPC5 using calcium influx analysis under agonist stimulation with 100 nmol/L Englerin A (EA). In comparison with HC-070, macrocycles macrocyclic compounds **5**–**7** show 2- to 60-fold increase in potency (Fig. 2F–I). Among them, compound **6** with 7-atom-length linked exhibited the strongest inhibitory activity. We further used the structure of the TRPC5/HC-070 complex (PDB: 7D4Q) to study the binding mode of compound **6** *via* molecular docking using Schrödinger. A receptor grid (20 Å × 20 Å × 20 Å) centered on the centroid of HC-070 was generated using the Receptor Grid Generation module. Molecular docking was performed using the standard precision mode of Glide, and the top-scoring result was selected as the docking pose. As expected, compound **6** adopts a conformation similar to HC-070 (Supporting Information Fig. S1A). However, the macrocyclic structure induces a planar conformation for the entire molecule, effectively stabilizing its overall conformation. These results validate the feasibility of the macrocyclization strategy.

### 2.2 Structure-based optimization of macrocyclic TRPC5 inhibitors

**Figure 3:**
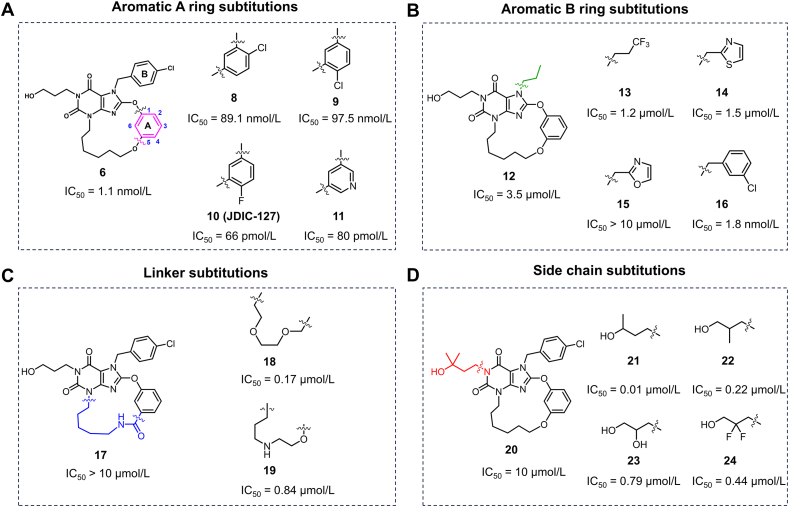
Structure–activity relationship exploration of macrocyclic TRPC5 inhibitors. (A, B) The chemical structure of aromatic A ring (A) and B ring (B) derivatives, and its inhibitory activity against TRPC5. (C, D) The chemical structure of linker substitutions (C) and side chain substitutions (D) derivatives, and their inhibitory activity against TRPC5.

With the primary results that 15-membered macrocyclic compound **6** exhibited the most potent inhibitory activity, further structural optimization based on compound **6** was conducted. The cryo-EM structure of TRPC5 in complex with HC-070 reveals that Y524 and L521 interact hydrophobically with the chlorophenyl ring of HC-070. Halogen substituents, such as chlorine (**8**, **9**), fluorine (**10**), were thereby introduced at the aromatic A ring substitutions of compound **2** (Fig. 3A). The results showed that chlorine atom substitution at 2-position (**8**) or 4-position (**9**) decreased the activity by about 90-fold. Surprisingly, the substitution of a smaller hydrophobic group with a fluorine atom at 4-position (**10**) increased the activity by approximately 15-fold, with an IC_50_ reaching the pmol/L level. Similar results were observed with the substitution of a smaller pyridine ring (**11**), which increased the activity by about 10-fold. These findings indicate that both steric effects and electronegativity may play crucial roles in modulating the compound's bioactivity.

We further investigated the impact of aromatic B ring substitutions on activity. Firstly, we replaced the chlorophenyl ring with hydrophobic alkyl side chains, and the results showed that both propyl (**12**) and trifluoropropyl substitutions (**13**) led to a decrease of over 1000-fold in activity (Fig. 3B). The molecular docking results for compound **12** indicate that, although its hydroxypropyl tail forms hydrogen bonds with Q573 and W577, it disrupts the *π*–*π* interaction with F576, resulting in a decrease in activity (Fig. S1B). We then explored the effects of different heterocyclic substitutions on activity, and the results revealed that only phenyl ring substitution (**16**) was tolerated. The introduction of thiazole ring (**14**) or oxazole ring (**15**) resulted in a more than 1000-fold decrease in activity. These findings suggest that the aromatic B ring may play a crucial role in maintaining the stability of the macrocyclic inhibitors.

With so strict requirements for the aromatic ring moiety to maintain the activity, the substitution effect on linker was explored next. We maintained the 16-membered ring size and replaced the linker with an amide, resulting in compound **17** completely abolishing the activity (Fig. 3C). This might be due to the hydrogen bond donor and acceptor properties of the amide disrupting the binding pose of the macrocycle. The molecular docking results indicate that the macrocyclic compound **17** adopts a distinctly different conformation upon binding to TRPC5 (Fig. S1C). We further replaced the hydrophobic carbon chain with a hydrophobic ether side chain (**18**), which also led to a 100-fold decrease in activity. Finally, we explored the impact of an amino linker on activity. Compound **19** also exhibits reduced activity. Molecular docking reveals that, although compound **19** adopts a favorable conformation, its positively charged side chain may interfere with interactions with the lipid components of the cell membrane (Fig. S1D). Additionally, we conducted further structure-activity relationship (SAR) studies on the hydroxypropyl group, finding that only 1-position methyl substitution (**21**) was tolerated; others led to decreased activity (Fig. 3D). Among all these SAR explorations, aromatic A ring substitution compounds **10** and **11** show the most potent TRPC5 inhibitory activity. Ultimately, compound **10** was selected for further studies. We refer to compound **10** as compound JDIC-127 henceforth.

### 2.3 Chemistry

**Scheme 1:**
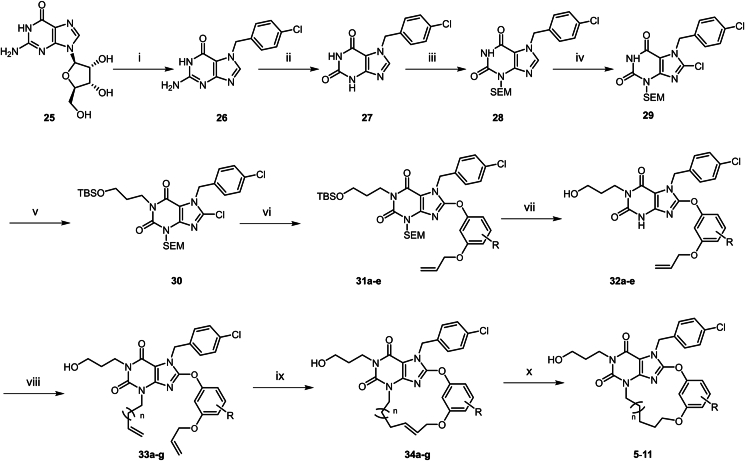
Synthesis of compounds **5**–**11**. Reagents and conditions: (i) 1-chloro-4-(chloromethyl)benzene, DMSO, 50 °C, 16 h; HCl (1 mol/L), 70 °C, 3 h; (ii) NaNO_2_, AcOH, H_2_O, 50 °C, 3 h; (iii) SEMCl, DBU, DMF, rt, 16 h; (iv) NCS, THF, rt, 2 h; (v) (3-bromopropoxy) (*tert*-butyl)dimethylsilane, K_2_CO_3_, DMF, 70 °C, 16 h; (vi) 3-(allyloxy)phenol, DMF, K_2_CO_3_, 80 °C, 16 h; (vii) HCl (12 mol/L), EtOH, 80 °C, 16 h; (viii) 4-bromobut-1-ene, DMF, Cs_2_CO_3_, 100 °C, 3 h; (ix) HG-II, DCM, 40 °C, 16 h; (x) Lindlar (5%), H_2_ (15 psi), EtOAc, rt, 1.5 h.

**Scheme 2:**
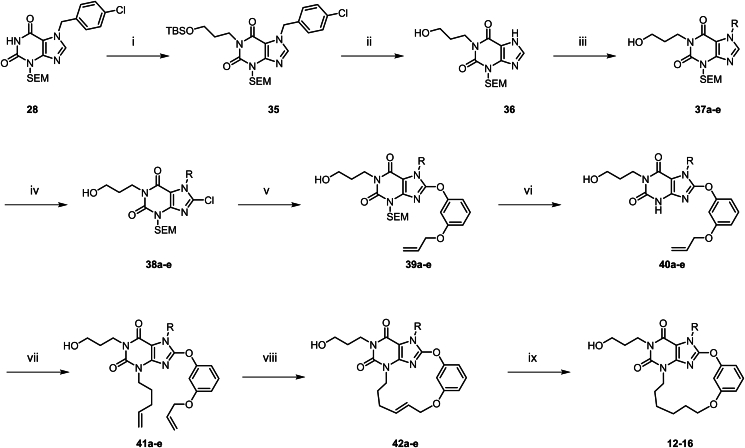
Synthesis of compounds **12**–**16**. Reagents and conditions: (i) (3-bromopropoxy) (*tert*-butyl)dimethylsilane, K_2_CO_3_, DMF, 80 °C, 16 h; (ii) Pd/C, H_2_ (50 psi), MeOH (10 V), 50 °C, 4 h; (iii) DMF, K_2_CO_3_ (2.0 eq.), rt, 16 h; (iv) NCS, THF, rt, 2 h; (v) 3-(allyloxy)phenol, DMF, K_2_CO_3_, 80 °C, 16 h; (vi) HCl (12 mol/L), EtOH, 80 °C, 16 h; (vii) 5-bromopent-1-ene, K_2_CO_3_, DMF, 80 °C, 1 h; (viii) HG-II, DCM, rt, 16 h; (ix) Lindlar (5%), H_2_ (15 psi), EtOAc, rt, 0.5 h.

**Scheme 3:**
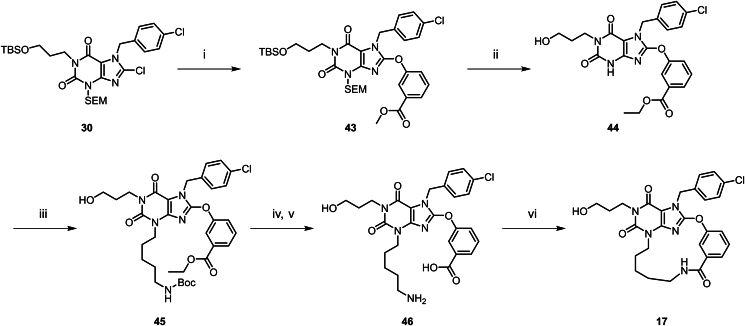
Synthesis of compound **17**. Reagents and conditions: (i) methyl 3-hydroxybenzoate, K_2_CO_3_, DMF, 80 °C, 5 h; (ii) HCl (12 mol/L), EtOH, 80 °C, 16 h; (iii) *tert*-butyl (5-bromopentyl)carbamate, Cs_2_CO_3_, DMF, 80 °C, 3 h; (iv) LiOH, THF/MeOH/H_2_O, rt, 1 h; (v) HCl/EtOAc (4 mol/L), EtOAc, rt, 2 h; (vi) T_3_P, DIEA, DCM, rt, 3 h.

**Scheme 4:**
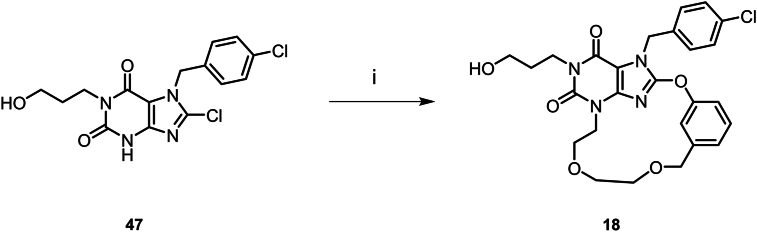
Synthesis of compound **18**. Reagents and conditions: (i) 3-((2-(2-bromoethoxy)ethoxy)methyl)phenol, K_2_CO_3_, DMF, 80 °C, 16 h.

**Scheme 5:**
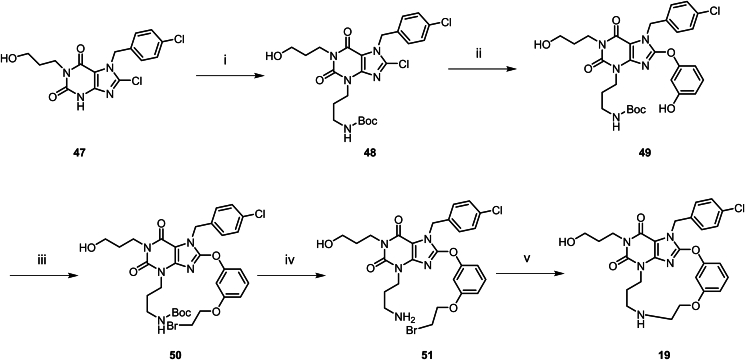
Synthesis of compound **19**. Reagents and conditions: (i) *tert*-butyl (3-bromopropyl)carbamate, Cs_2_CO_3_, DMF, 80 °C, 1 h; (ii) resorcinol, K_2_CO_3_, MeCN, 80 °C, 2 h; (iii) 1,2-dibromoethane, K_2_CO_3_, MeCN, 80 °C, 16 h; (iv) HCl/dioxane (4 mol/L), DCM, rt, 2 h; (v) K_2_CO_3_, MeCN, 50 °C, 16 h.

**Scheme 6:**
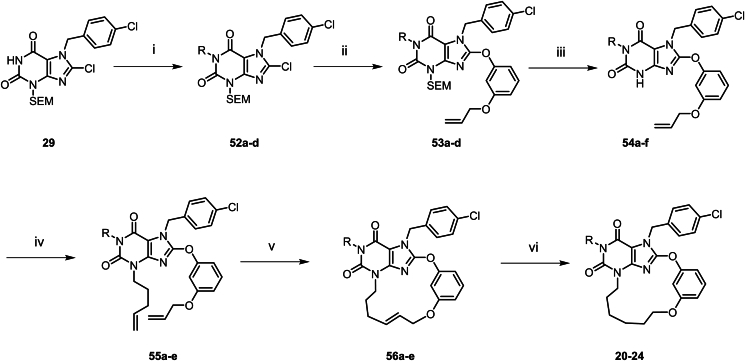
Synthesis of compounds **20**–**24**. Reagents and conditions: (i) 4-bromo-2-methylbutan-2-ol, K_2_CO_3_, DMF, 80 °C, 3 h; (ii) 3-(allyloxy)phenol, K_2_CO_3_, DMF, 80 °C, 16 h; (iii) HCl (12 mol/L), EtOH, 80 °C, 16 h; (iv) 5-bromopent-1-ene, Cs_2_CO_3_, DMF, 80 °C, 1 h; (v) HG-II, DCM, rt, 16 h; (vi) Lindlar (5%), H_2_ (15 psi), EtOAc, rt, 0.5 h.

The synthesis of macrocyclic TRPC5 inhibitors is shown in Scheme 1, Scheme 2, Scheme 3, Scheme 4, Scheme 5, Scheme 6. Compounds **5**–**11** were prepared according to Scheme 1. The commercially available compound **25** underwent a substitution reaction with 4-chlorobenzyl chloride to afford **26**, which underwent diazotization-hydrolysis to afford **27**. The amide nitrogen atom in **27** was then protected with a SEM group, followed by chlorination under NCS conditions to give **29**. Compound **29** underwent a substitution reaction with (3-bromopropoxy) (*tert*-butyl)dimethylsilane to form **30**, which further reacted with diversely substituted 3-(allyloxy)phenol *via* substitution to generate various substituted derivatives **31**. The SEM and TBS protecting groups in **31** were removed under 12 mol/L HCl in ethanol, resulting in the substituted intermediates **32**. Next, **32** reacted with alkyl bromides *via* substitution to produce compounds **33a**–**g**. Under the second-generation Grubbs catalyst, **33a**–**g** underwent Grubbs olefin metathesis to cyclize into the macrocyclic intermediates **34a**–**g**. Finally, the double bond in **34a**–**g** was hydrogenated under Lindlar catalyst conditions, reducing it to furnish the target macrocyclic compounds **5**–**11**.

Compounds **12**–**16** were prepared according to the procedures in Scheme 2. The synthesis method of this series of compounds is analogous to Scheme 1, with the difference being that different haloalkane substituents were introduced *via* compound **36**. Subsequently, the target compounds were obtained through chlorination, phenolic group substitution, deprotection, Grubbs olefin metathesis and hydrogenation using Lindlar catalyst. Compound **17** was prepared according to Scheme 3. Compound **30** undergoes a substitution reaction with methyl 3-hydroxybenzoate to yield **43**. Subsequently, under 12 mol/L HCl in ethanol conditions, the SEM and TBS protecting groups are removed to afford **44**. An amino side chain is then introduced to obtain **45**. The Boc protecting group is cleaved, and the ester group is hydrolyzed to give **46**. Finally, under T3P conditions, an amide condensation reaction is performed to yield compound **17**.

Compound **18** was prepared according to Scheme 4. In DMF solvent, compound **47** undergoes a one-step cyclization reaction with 3-[2-(2-bromoethoxy)ethoxymethyl]phenol to directly afford compound **18**. Compound **19** was prepared according to Scheme 5. Compound **47** undergoes a substitution reaction to introduce an amino side chain, yielding **48**. This is followed by a reaction with resorcinol to afford **49**. The phenolic hydroxyl groups of **49** are further substituted with dibromoethane to give **50**. After removal of the Boc protecting group, **50** undergoes a substitution reaction with an alkane bromo side chain, which subsequently undergoes cyclization to yield compound **19**. Compounds **20**–**24** were synthesized according to the procedures in Scheme 6. The synthesis of this series of compounds is analogous to Scheme 1, with the difference that diverse substituent side chains are introduced *via* intermediate **29**. Subsequent reactions follow the same pathway as Scheme 1, involving Grubbs olefin metathesis and hydrogenation with Lindlar catalyst to yield the target compounds.

### 2.4 *In vitro* pharmacological and selectivity profiling of JDIC-127

**Figure 4:**
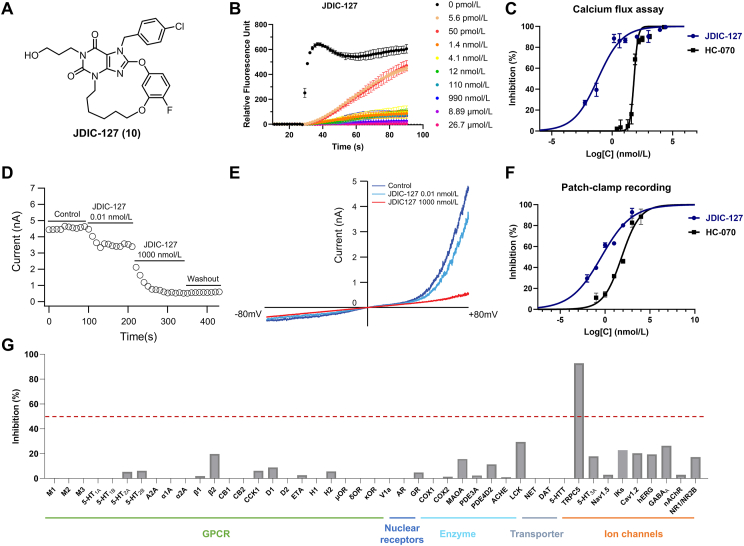
Functional characterization of JDIC-127. (A) The chemical structure of JDIC-127. (B) Dose-response curves of TRPC5 channel inhibition by JDIC-127. (C) Curve fitting of dose-dependent JDIC-127 inhibition of TRPC5 calcium fluorescence signal evoked by 100 nmol/L Englerin A (EA). (D) Example whole-cell patch-clamp data from a TRPC5-expressing HEK 293T cell showing current sampled. (E) Whole-cell patch-clamp recordings in response to a −80 to +80 mV voltage ramp. (F) Dose-response curves of TRPC5 channel inhibition by JDIC-127 in whole-cell patch-clamp experiments by 100 nmol/L EA. (G) The selectivity of JDIC-127 against safety-related targets, including GPCRs, nuclear receptors, enzymes and transporters at 10 μmol/L, and ion channels at 5 μmol/L.

As demonstrated in the SAR studies, JDIC-127 inhibited calcium influx in cells stably expressing TRPC5 channels, with an IC_50_ of 66 pmol/L in a concentration-response study (Fig. 4A–C). More than 80% inhibition was observed at concentrations of 1.4 nmol/L or higher (Fig. 4B). Given that patch-clamp experiments are considered the gold standard for evaluating ion channel modulators^42^, We further assessed the inhibitory activity of JDIC-127 on TRPC5 using whole-cell patch-clamp recordings in TRPC5-stably transfected HEK293T cells under agonist stimulation with 100 nmol/L EA. The results demonstrated that JDIC-127 exhibited potent inhibition of TRPC5, with an IC_50_ of 374 pmol/L (Fig. 4D–F). For comparison, we also evaluated the inhibitory activity of compound HC-070 on TRPC5 under the same conditions. The data revealed that JDIC-127 was approximately 200-fold more potent than HC-070 in inhibiting TRPC5 (IC_50_ = 70.9 nmol/L) (Fig. 4F). Several studies have demonstrated that changes in calcium ion concentration regulate TRPC5 channel activation^43^^,^^44^. TRPC5 channels can be directly activated by increased intracellular Ca^2+^ levels. Accordingly, we evaluated JDIC-127-mediated TRPC5 inhibition under endogenous Ca^2+^-evoked activation conditions, revealing an IC_50_ of 1.4 pmol/L (Supporting Information Fig. S2A and S2B). These results further validate the high potency of JDIC-127 against TRPC5.

Given that TRPC4 and TRPC5 are highly similar and often form heteromeric with TRPC1, we evaluated JDIC-127's activity on TRPC4, TRPC1/4, and TRPC1/5 using whole-cell patch-clamp recordings under agonist stimulation with 100 nmol/L EA in HEK293T cells. The results demonstrated that JDIC-127 also effectively inhibits TRPC4, TRPC1/4, and TRPC1/5 with IC_50_ values of 0.001, 1.86, and 0.358 nmol/L, respectively (Fig. S2C–S2H).

Previous studies have reported that HC-070 exhibits moderate inhibitory activity against hERG and TRPC3, with IC_50_ values below 2 μmol/L^30^, highlighting potential safety concerns associated with its use. In contrast, macrocyclic compounds are known for their diverse functionality, stereochemical complexity, and semi-rigid, preorganized structures, which confer enhanced selectivity in target binding compared to their ring-opened counterparts.

To comprehensively evaluate the selectivity and safety profile of JDIC-127, we utilized the ICESTP™ SafetyOne44 panel to assess its activity against a wide range of safety-related targets, including GPCRs, nuclear receptors, enzymes, transporters, and ion channels. At concentrations of 10 and 5 μmol/L, JDIC-127 exhibited no significant inhibitory or stimulatory effects on any of the 44 targets tested, all of which are commonly associated with clinical adverse drug reactions (ADRs) (Fig. 4G, Supporting Information Tables S1 and S2). In addition, the selectivity of JDIC-127 within the other TRP channels and its potential impact on hERG were further investigated through patch-clamp electrophysiology. At a concentration of 1 μmol/L, JDIC-127 inhibited less than 20% of TRPC3 and TRPC6 activity (Supporting Information Fig. S3), while at 10 μmol/L, it inhibited less than 33% of hERG, TRPV1 and TRPM3 activity (Supporting Information Fig. S4). These results underscore the high selectivity of JDIC-127, which likely contributes to its favorable *in vitro* safety profile, distinguishing it as a promising therapeutic candidate with reduced risk of off-target effects.

### 2.5 Cryo-EM structure of JDIC-127 bound to TRPC5

**Figure 5:**
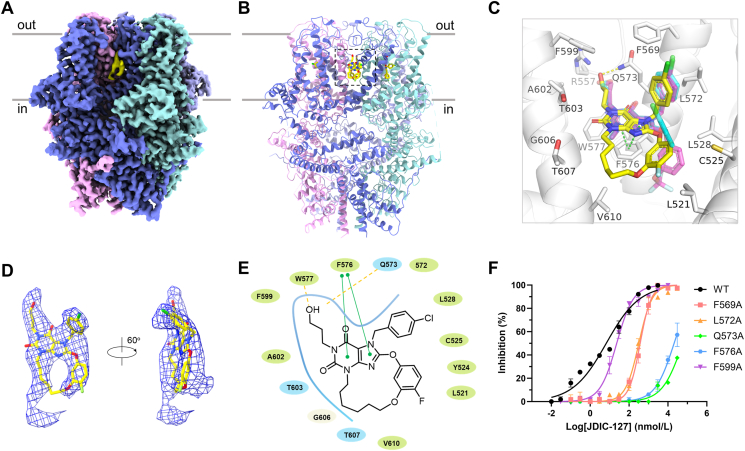
Structure of TRPC5/JDIC-127 complex. (A) Cryo-EM density maps of TRPC5 in complex with JDIC-127 (yellow), reconstructed and contoured at 3*σ*. The subunits are colored by domain. (B) Ribbon diagram of JDIC-127-bound TRPC5 structure is shown. (C) Detailed view of the binding site, illustrating the interactions between JDIC-127 (yellow sticks) and TRPC5. For comparison, the binding conformations of HC-070 (cyan sticks, PDB: 7D4Q) and Pico145 (purple sticks, PDB: 6YSN) with TRPC5 are also displayed. The side chains of crucial residues are shown in sticks. (D) Cryo-EM density maps for JDIC-127 (yellow stick) presented from different angle contoured at 3*σ*. (E) Cartoon representation of the interaction between JDIC-127 and TRPC5. Green solid lines represent *π*–*π* stacking interaction. Yellow dashed lines represent hydrogen bond interactions. (F) The inhibitory effects of JDIC-127 on various TRPC5 mutants, *n* = 3 biological replicates.

To validate the macrocyclic-based design of JDIC-127, we determined the cryo-EM structure of TRPC5 in complex with JDIC-127. For this structural study, we used a C-terminally truncated mouse TRPC5 construct that retains functionality^39^. TRPC5 was extracted from the membranes of HEK293S cells using glyco-diosgenin (10.13039/501100015615GDN) detergent and subjected to cryo-EM in the presence of 50 μmol/L JDIC-127 (Supporting Information Fig. S5). The cryo-EM analysis yielded a 3.43 Å resolution map, enabling clear visualization of four protein subunits and distinct ligand density at the portal site (Fig. 5A, Supporting Information Fig. S6, and Supporting Information Table S3). The ligand density was consistent with the molecular structure of JDIC-127, allowing precise placement and characterization of its binding mode (Fig. 5A–D). Comparison with lipid density in the TRPC5 apo state (EMD-9615) also revealed additional lipid-like densities extending forward and backward from the JDIC-127 density (Fig. 5D, Supporting Information Fig. S7A and S7B). This may be due to incomplete occupancy of the binding pocket by JDIC-127, resulting in partial occupancy. Similar to HC-070, JDIC-127 binds to the canonical binding site, formed by the S5 helix of one subunit and the pore helix and S6 of the adjacent subunit (Fig. 5B and C). The ligand is stabilized through *π*–*π* stacking interactions with F576 and hydrogen bonding with the side chains of Q573 and W577 (Fig. 5E). Unlike the clover-shaped conformation of HC-070, JDIC-127 adopts a flatter macrocyclic conformation, potentially reducing benzene ring movement and minimizing entropy loss. Comparative structural alignment of JDIC-127, HC-070 and Pico145 binding modes, the binding conformation of JDIC-127 is closer to that of Pico145, further validating the high activity of JDIC-127 (Fig. 5F).

During our 3D classification, we identified a novel 3D class featuring two TRPC5 tetramers stacked together. We further processed and modeled this structure yielded a 3.23 Å resolution map (Fig. S6). The structure reveals that the extracellular domains interact in a head-to-head manner *via* loop-mediated interactions, and each tetramer binds four JDIC-127 molecules (Supporting Information Fig. S8A and S8B). Compared to the tetramer map, the JDIC-127 density within the head-to-head TRPC5/JDIC-127 map displays a more complete molecular density, potentially due to better occupancy (Fig. S7C). Further analysis of the binding interface and JDIC-127 binding pockets demonstrates that E467 in the extracellular ECL2 loop forms a salt bridge interaction with R545 from the opposing subunit. Additionally, intrasubunit interactions between E549 and K591 enable I547 in the loop to extend toward the opposing subunit, forming hydrophobic interactions (Fig. S8C). Analysis of the JDIC-127 binding pockets reveals interactions similar to those observed in individual tetramer subunits (Fig. S8D and S8E).

Comparison of the TRPC5/JDIC-127 complex with previously resolved structures of TRPC5 bound to HC-070^41^ and in the apo state^39^ revealed significant conformational differences, with RMSD values of 3.1 and 3.4 Å, respectively (Supporting Information Fig. S9A). The S1–S6 helices, along with the coiled-coil domain, exhibit pronounced shifts in pitch, resulting in an upward-shifted conformation for the TRPC5/JDIC-127 structure compared to the HC-070-bound and apo states (Fig. S9B–S9E). The selectivity filter, defined by G581, expands by 2 Å, while the lower gate, formed by N625, contracts by 0.6 Å in the JDIC-127-bound structure. A similar pattern is observed in the TRPC5/HC-070 structure compared to the apo state, with widening of the selectivity filter and tightening of the lower gate. Despite these structural alterations, the TRPC5/JDIC-127 complex remains in a closed-state conformation (Supporting Information Fig. S10).

To further investigate the binding interactions, we introduced point mutations at residues implicated in JDIC-127 recognition and evaluated their impact on channel inhibition by JDIC-127 (Fig. 5F). Mutations such as Q573A and F576A significantly diminished the inhibitory potency of JDIC-127, underscoring the critical contributions of hydrogen bonding and *π*–*π* stacking interactions to ligand binding.

### 2.6 Pharmacokinetic properties and brain penetrant study of JDIC-127

Given its remarkable *in vitro* TRPC5 inhibitory activity, we subsequently evaluated the pharmacokinetic properties of JDIC-127. Pharmacokinetic studies were performed in male C57BL/6 mice following intravenous (IV) or oral (PO) administration of JDIC-127 at doses of 2 and 10 mg/kg (Supporting Information Fig. S11). The calculated pharmacokinetic parameters are summarized in Table 1. Since TRPC5 channels are predominantly expressed in the brain, we further investigated the brain penetration of JDIC-127 (Table 2). Following intravenous administration, JDIC-127 demonstrated a brain-to-plasma (B/P) ratio of 3.85 at 45 min. Similarly, after oral administration, the compound exhibited a B/P ratio of 2.51 at the same time point. These findings indicate that JDIC-127 possesses favorable pharmacokinetic characteristics, including significant brain permeability, supporting its potential utility in *in vivo* studies for the treatment of depression and other neuropsychiatric disorders.

**Table 1:** Pharmacokinetic properties of JDIC-127.

Administration	*C*_0_ (ng/mL)	*T*_max_ (h)	*C*_max_ (ng/mL)	AUC_0–*t*_ (h∗ng/mL)	AUC_0–∞_ (h∗ng/mL)	MRT (h)	*T*_1/2_ (h)	*V*_d_ (mL/kg)	CL (mL/h/kg)	*V*_SS_ (mL/kg)	ER	*P*_app_ (cm/s)	*F* (%)
C57BL/6 (10 mpk, *p.o.*)	/	0.25	177	187	193	1.9	1.4	107000	51900	/	1.45	1.51 × 10^−6^	7.5
C57BL/6 (2 mpk, i.v.)	1450	/	/	496	499	0.88	0.99	5710	4010	3510

**Table 2:** Brain permeability of JDIC-127.

Administration	Dose (mg/kg)	Time (min)	*C*_pl_ (nmol/L)	*C*_br_ (nmol/kg)	B/P
*p.o.*	10	2	6.36	22.16	3.48
		45	88.83	223.33	2.51
		180	22.96	7.35	0.32
i.v.	2	2	367.10	407.01	1.1
		45	51.63	198.65	3.85
		180	10.12	74.96	7.4

### 2.7 *In vivo* efficacy of JDIC-127 on antidepressant and anxiolytic activity

**Figure 6:**
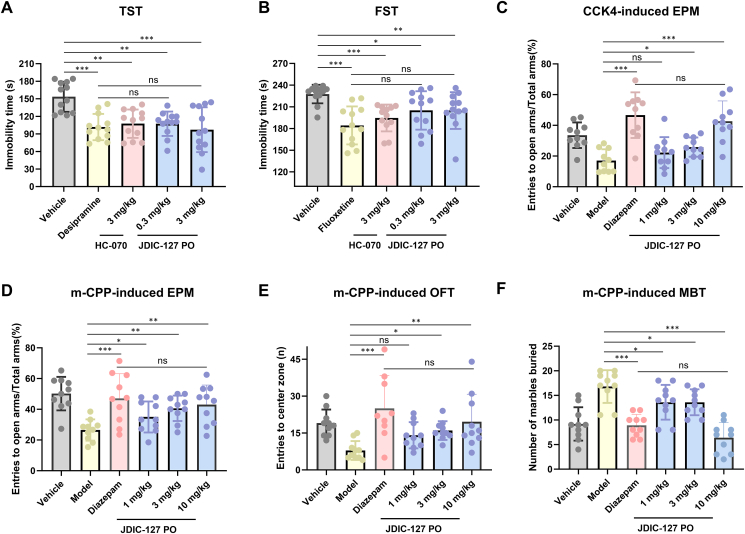
The antidepressant and anxiolytic effects of JDIC-127. (A) Effects of compounds on immobility time in tail suspension test (TST). Mice were administered vehicle, 3 mg/kg HC-070 and 10 mg/kg desipramine, 0.3 or 3 mg/kg JDIC-127 oral administration (*p*.*o*.) 60 min prior to testing. Data are expressed as the mean ± SEM (*n* = 12). (B) Effects of compounds on immobility time in forced swim test (FST). Mice were administered continuously for 14 days with vehicle, 3 mg/kg HC-070 and 1.58 mg/kg fluoxetine, 0.3 or 3 mg/kg JDIC-127 *p*.*o*. 60 min prior to testing. Data are expressed as the mean ± SEM (*n* = 12). (C) Effects of compounds on the percentages of open arm time and entries in the elevated plus maze (EPM) in the cholecystokinin tetrapeptide (CCK-4)-induced anxiety model. Mice were administered vehicle, 0.3 mg/kg CCK-4 intraperitoneal (i.p.) in the model group, 1.5 mg/kg diazepam ip 30 min prior to behavioral testing, 1, 3 or 10 mg/kg JDIC-127 *p*.*o*. 60 min prior to testing. Data are expressed as the mean ± SEM (*n* = 10). (D–F) Effects of compounds on the percentages of open arm time and entries in EPM, number of entries into center zone in open field test (OFT), number of marbles buried in Marble Burying Test (MBT) in the meta-chlorophenylpiperazine (m-CPP)-induced anxiety model. Mice were administered vehicle, 1 mg/kg m-CPP i.p*.* in the model group, 1.5 mg/kg diazepam i.p. 30 min prior to behavioral testing, 1, 3 or 10 mg/kg JDIC-127 *p*.*o*. 60 min prior to testing. Data are expressed as the mean ± SEM (*n* = 10) ∗*P* < 0.05, ∗∗*P* < 0.01, ∗∗∗*P* < 0.001. Statistical analysis was performed using one-way ANOVA.

We further investigated the antidepressant effects of JDIC-127 in behavioral studies, employing HC-070 as a reference compound. Depressive-like behaviors in mice were evaluated using the tail suspension test (TST) and forced swim test (FST). JDIC-127 significantly reduced immobility time in both the TST (Fig. 6A) and FST (Fig. 6B), indicative of robust antidepressant activity. Notably, Oral administration of JDIC-127 achieved comparable efficacy to desipramine and HC-070 but at a lower dose (0.3 mg/kg) in the TST.

TRPC5, a receptor-activated non-selective cation channel, serves as a downstream signaling component for multiple GPCRs, including CCK_2_R, mGluR, and 5-HT_2C_R^28^^,^^45^. It can be can be activated by GPCR–G*α*_q_–PLC pathway. To evaluate the *in vivo* efficacy and mechanism of JDIC-127, we employed cholecystokinin tetrapeptide (CCK-4)-induced CCK_2_R activation model and meta-chlorophenylpiperazine (m-CPP)-triggered 5-HT receptor activation model. In the CCK4-induced anxiety model, the anxiolytic effects of JDIC-127 were also assessed using the elevated plus maze (EPM) test^46^. Administration of 10 mg/kg *p.o.* JDIC-127 significantly increased the percentage of entries in the open arms (Fig. 6C). In the m-CPP induced anxiety model, we evaluated the effects of the compound using the EPM, Open Field Test (OFT), and Marble Burying Test (MBT). In the EPM and OFT experiments, JDIC-127 significantly increased the percentage of entries into the open arms and the number of entries into the central area at 10 mg/kg *p.o* (Fig. 6D and E). In the MBT experiment, JDIC-127 reduced the number of buried marbles (Fig. 6F). Overall, these findings suggest that inhibition of TRPC5 by JDIC-127 alleviates depression- and anxiety-like behaviors in rodent models.

## 3 Conclusions

Recent advances in ion channel structural biology have significantly accelerated structure-based drug design^47^^,^^48^. However, the intrinsic diversity and structural characteristics of ion channel binding pockets—characterized by their large, flat surfaces and frequent occupation by lipids—pose considerable challenges for medicinal chemists^49^. Targeting lipid-occupied binding sites, in particular, has long been regarded as one of the most difficult obstacles in developing potent and selective small molecules for ion channels.

TRPC5 has emerged as a promising therapeutic target, particularly due to its high expression in brain regions such as the amygdala, which are involved in regulating negative emotions like anxiety and depression^32^^,^^33^^,^^50^. TRPC5 is activated by G_q_-coupled GPCRs^28^^,^^29^^,^^45^, such as CCK_2_R, mGluR, and 5-HT_2C_R, serving as a critical ion channel for converting chemical signals into electrical activity in the human brain. Studies demonstrated that TRPC5 inhibitors or TRPC5 knockout mice exhibited significant suppression of CCK-4-induced neuronal currents^28^^,^^30^. This functional role highlights its potential as a novel target for neuropsychiatric disorders, including anxiety and depression. However, current preclinical candidates such as HC-070^30^, are limited by moderate off-target effects. HC-070 exhibits significant inhibition of hERG channels and related TRPC family members, such as TRPC3, with IC_50_ values below 2 μmol/L, underscoring the need for inhibitors with greater potency and selectivity^30^. Structural biology studies have revealed that HC-070 binds to a lipid-occupied pocket, displacing a lipid molecule in the apo state^39^. Specifically, HC-070 binds near the pore helix at the interface between the S5 helix of one subunit and the S6 helix of an adjacent subunit, adopting a cloverleaf-like conformation. Medicinal chemists face considerable challenges in improving both the activity and selectivity of small molecules targeting such binding sites.

In this study, a series of macrocyclic TRPC5 inhibitors was developed using a structure-guided macrocyclization approach. Macrocyclic compounds offer distinct advantages by stabilizing conformational flexibility, enabling ligands to adopt preorganized conformations optimized for interaction with challenging binding pockets. Among the inhibitors developed, JDIC-127 demonstrated exceptional potency, with an IC_50_ in picomolar range. TRPC5 is highly homologous to TRPC4, and both are involved in the neurobiological mechanisms underlying the regulation of anxiety and depression. They can also form heterotetrameric channels with TRPC1. Our experimental results demonstrate that the compound JDIC-127 also effectively inhibits TRPC4, TRPC1/4, and TRPC1/5 channels, exhibiting IC_50_ values in the picomolar to low nanomolar range—indicating exceptionally high inhibitory potency. The selectivity of JDIC-127 was validated by its minimal activity against other TRP channels and ion channels. This remarkable selectivity is likely due to JDIC-127's ability to exploit unique features of TRPC5's binding pocket, particularly at the portal site involving the S5 and S6 helices.

TRPC5 is a key downstream effector of multiple GPCRs. We utilized CCK-4- and m-CPP-induced behavioral paradigms, both of which are known to engage TRPC5 *via* activation of CCK2R and 5-HT_2C_R, respectively. JDIC-127 significantly ameliorated anxiety-like behaviors in these models across multiple assays, including EPM, OFT, and MBT, supporting a TRPC5-dependent mechanism of action. In summary, TRPC5 functions downstream of multiple G*α*_q_-coupled GPCRs, mediating calcium influx and regulating neuronal excitability. JDIC-127 is proposed to exert its antidepressant and anxiolytic effects through inhibition of TRPC5 channel activity, thereby dampening excessive calcium entry and normalizing neuronal hyperexcitability.

Emerging evidence underscores the critical role of lipid molecules, such as phosphatidylinositol phosphates (PIPs) and cholesterol, in nearly all resolved ion channel structures^49^. These lipids are integral to gating, conductance, and overall channel activity. For example, in TRPV1, the vanilloid-binding pocket is occupied by phosphatidylinositides in the closed state^51^, which are displaced by vanilloid agonists to induce conformational changes and channel opening. Similar lipid interactions have been observed in TRPML1^52^, TRPM7^53^, and PKD2 channels^54^, where lipid molecules bind to open, flat binding pockets, complicating drug design. This study highlights the potential of macrocyclic drug design to address these challenges. JDIC-127 exemplifies how macrocyclization can enhance ligand affinity and selectivity by stabilizing interactions within lipid-occupied or lipid-like binding pockets. This approach not only advances the pharmacology of TRPC5 but also offers a promising strategy for other TRP channel families, including TRPV, TRPM, and TRPML, where vanilloid-like pockets or portal sites present viable therapeutic targets^55^.

By leveraging advanced structural biology techniques, such as cryo-EM, alongside computational modeling and SAR studies, it is now possible to address longstanding challenges in ion channel drug discovery. JDIC-127 serves as a proof-of-concept for the application of macrocyclization in the development of innovative therapeutics. Beyond advancing TRPC5 pharmacology, this work lays the foundation for broader applications of macrocyclic compounds in targeting other ion channels, addressing critical limitations in efficacy and side effects. The insights gained from this study provide a roadmap for the future development of ion channel-targeted therapeutics, with implications extending to a wide range of diseases beyond neuropsychiatric disorders.

## 4 Experimental

### 4.1 Chemistry

All starting materials, solvents, and reagents were used directly as obtained commercially without further purification unless otherwise noted. NMR spectra were recorded using CDCl_3_, DMSO-*d*_6_, or MeOD-*d*_4_ on a Bruker Avance 400 MHz spectrometer. Chemical shifts are reported in parts per million referenced with respect to residual solvent (MeOD-*d*_4_) 3.31 ppm, (DMSO-*d*_6_) 2.50 ppm, and (CDCl_3_) 7.26 ppm. Coupling constants (*J*) are expressed in hertz (Hz). Chemical shifts (*δ*) of NMR are reported in parts per million (ppm) units. The first-order peak patterns are indicated as s (singlet) and d (doublet). All compounds submitted for testing were confirmed to be >95.0% purity by HPLC traces.

### 4.2 Calcium flux assay

Intracellular calcium levels were measured using the Calcium 5 Assay Kit (Molecular Devices). Wild-type TRPC5 and TRPC5 mutant constructs (vector; pcDNA3.1) were transfected into HEK 293T cells with PEI transfection reagent. The transfected cells were then plated in a 96-well, clear-bottomed, poly-l-lysine-coated black plate (Costar blk/clrbtm). Cells were cultured overnight in DMEM (Corning) supplemented with 10% FBS (ExCell Bio) at 37 °C in a 5% CO_2_ incubator. Following medium removal, 100 μL of loading buffer (provided in the kit) was added to each well, and plates were incubated for 1 h at 37 °C in 5% CO_2_. Molecules were added to each well 5 min before measurement. To activate the TRPC5 channel, 100 nmol/L Englerin A was used. Fluorescence signals were recorded using a FlexStation 3 (Molecular Devices) with excitation and emission wavelengths set to 485/525 nm, and an automatic emission filter at 515 nm.

### 4.3 Whole cell electrophysiology

For electrophysiological activity testing of compounds against TRPC4 and TRPC5, stably expressed TRPC5 or TRPC4 cells were used for whole-cell patch-clamp recordings, employing a HEKA EPC10 amplifier and PATCHMASTER software (https://www.heka.com/downloads/downloads_main.html#down_patchmaster). Prior to recording, cells were induced with 1 μg/mL tetracycline for 24 h. For TRPC1/4 or TRPC1/5 heteromers, plasmids TRPC1 and TRPC5 or TRPC1 and TRPC4 were co-transfected into HEK293T cells. After 48 to 72 h of transfection, testing was performed. A 1-s ramp protocol from −100 to +100 mV at 0.2 Hz was applied, as previously described^39^. The extracellular solution consisted of 140 mmol/L NaCl, 1 mmol/L MgCl_2_, 2 mmol/L CaCl_2_, 10 mmol/L Hepes, 5 mmol/L KCl, and 10 mmol/L d-glucose, with pH adjusted to 7.4 using NaOH. The pipette solution contained 130 mmol/L CsCl, 1 mmol/L MgCl_2_, 5.7 mmol/L CaCl_2_, 10 mmol/L EGTA, 10 mmol/L Hepes, and pH adjusted to 7.2 using CsOH. Recordings were conducted at room temperature, and the recording chamber (150 μL) was perfused at a rate of approximately 2 mL/min. Signals were sampled at 50 kHz and filtered at 2.9 kHz. The half-maximal inhibitory concentration (IC_50_) of drugs was determined by recording currents at a +100 mV pulse and analyzing current amplitudes at varying drug concentrations, with IC_50_ values calculated using the Hill equation. Englerin A (100 nmol/L) was used to activate TRPC4, TRPC5, TRPC1/4 and TRPC1/5 channels. For Ca^2+^-activated condition, the pipette solution contained 130 mmol/L CsCl, 1 mmol/L MgCl_2_, 8.7 mmol/L CaCl_2_, 10 mmol/L EGTA, 10 mmol/L Hepes, and pH adjusted to 7.2 using CsOH.

### 4.4 Selectivity evaluation of JDIC-127 against safety related targets

The ICESTP™ SafetyOne44 Panel developed by ICE Bioscience was used to assess the selectivity against 44 clinically safety-related targets, including GPCRs, nuclear hormone receptors, ion channels, transporters, kinases and enzymes.

### 4.5 Construct

A DNA segment encoding the truncated TRPC5 channel was synthesized and cloned into the pEGBacMam vector. To enhance protein purification, a MBP tag was appended to the N-terminus of TRPC5 through a linker featuring the HRV 3C cleavage site. For electrophysiological experiments, full-length wild-type or mutant TRPC5 was cloned into a pcDNA3.1 vector. For the heteromers, an mCherry tag was inserted into the TRPC5 or TRPC4 constructs, and a GFP tag was inserted at the C-terminus of TRPC1, all these genes were cloned into the pcDNA3.1 vector.

### 4.6 Expression and purification

The MBP-TRPC5 protein was expressed and purified based on our previously established protocols^39^^,^^56^. Briefly, HEK293S GnTI^−^ cells were transduced with 10% (*v*/*v*) P4 baculovirus at a density of 2.0–3.0 × 10^6^ cells/mL. Twenty-four hours after transduction, 10 mmol/L sodium butyrate was added to boost protein expression and harvested at 72 h. Cells were collected using a centrifuge at 5840×*g* for 10 min at 4 °C. The cell pellet was re-suspended in buffer A (50 mmol/L Hepes pH 7.5, 150 mmol/L NaCl, 1 mmol/L DTT, 10% (*v*/*v*) glycerol, 1% (*v*/*v*) EDTA-free protease inhibitor cocktail, 1% (*w*/*v*) GDN) and then solubilized for 3 h in 4 °C. Then the cell lysate was centrifuged for 50 min at 100,000×*g*, and the supernatant was incubated with MBP resin at 4 °C overnight. The resin was packed onto a disposable gravity column (Bio-Rad) and washed with 10 column volumes of wash buffer (0.02% (*w*/*v*) GDN, 150 mmol/L NaCl, 1 mmol/L DTT, and 25 mmol/L Hepes pH 7.5) and eluted with elute buffer (0.02% (*w*/*v*) GDN, 150 mmol/L NaCl, 1 mmol/L DTT, and 25 mmol/L Hepes pH 7.5, 40 mmol/L maltose). All purification procedures were carried out on ice or at 4 °C. The eluted TRPC5 protein was collected, concentrated, and further purified by size-exclusion chromatography on a Superose 6 column (GE Healthcare) pre-equilibrated with SEC buffer (0.02% (*w*/*v*) GDN, 150 mmol/L KCl, 1 mmol/L DTT, and 25 mmol/L Hepes pH 7.5). Peak fractions were pooled and concentrated to 9 mg/mL. Additionally, 50 μmol/L JDIC-127 was spiked into the protein sample prior to EM grid preparation.

### 4.7 Cryo-EM sample preparation and data acquisition

To prepare cryo-EM grids, 3 μL of the samples were applied to Quantifoil grid R1.2/1.3 Au 300 mesh grids (glow discharged at 15 mA for 40 s with a Glow discharge cleaning system). Grids were blotted with qualitative filter paper in a Vitrobot Mark Ⅳ (Thermo Fisher Scientific) at 4 °C and 100% humidity for 3 s using a blot force of 2 prior to plunging into liquid ethane and stored in liquid nitrogen until checked. For cryo-EM data acquisition, images were collected on a Titan Krios electron microscope (FEI) equipped with a K3 direct electron detector (Gatan) at 130,000 × magnification (pixel size of 0.96 Å/pixel) with EPU data collection software (Thermo Fisher). The movie stacks were automatically acquired with the defocus range from −1.0 to −2.0 μm. Micrographs were collected with a total dose of ∼50.54 e^−^/Å^2^.

### 4.8 Imaging processing and 3D reconstruction

A total of 5898 movie stacks were imported into cryoSPARC v4.1.1^57^. After motion correction, electron-dose weighted and CTF estimation, the initial particles were performed by cryoSPARC blob picker. After 2D classification, we picked 2D classes with channel features as templates. And the particles were performed by cryoSPARC template picker. After four rounds of 2D classification, the good particles proceeded to two rounds of *ab initio* reconstruction and heterogeneous refinement. The particles of the best classes were re-extracted with original box size and further applied for final nonuniform refinement and local refinement. Eventually, two different density maps were obtained. Among them, we found a type of head-to-head structure. We applied C1 and C4 symmetric reconstruction to it, and there was no significant difference in density. The overall resolutions of these two types of density maps are 3.43 and 3.23 Å (determined by gold standard Fourier shell correlation (FSC) using the 0.143 criterion).

### 4.9 Model building

The reference model (PDB: 7D4Q) was rigid-body fit into the EM density map using Chimera. The fit was further adjusted using the jiggle fit function in Coot^58^. Further manual adjustment with the real-space refine zone function in Coot was used to generate an atomic model. The generated model was further refined using the real_space_refine tool in Phenix^59^. MolProbity^60^ and Mtriage were used for validation. PyMOL and Chimera^61^ were used to further analyze the structure and generate figures.

### 4.10 Molecular docking

The cryo-EM structure of TRPC5/HC-070 (PDB: 7D4Q) was prepared in the protein preparation Wizard Workflow of Glide in Maestro (Schrödinger Release 2021-2)^62^. The energy of the protein structure was minimized using the OPLS4 force field until the RMSD of the heavy atoms converged to 0.3 Å. The center of the three-dimensional receptor grid (20 Å × 20 Å × 20 Å) was generated by centroid of HC-070 *via* the Receptor Grid Generation module. The LigPrep module of Maestro was used to generate the three-dimensional (3D) conformations and apply the OPLS4 force field to minimize the energy of the molecules. During the process, ligands were prepared at pH 7.0 ± 2.0 to estimate their protonation using the Epik module and with maximal 32 stereoisomer generations. Molecular docking was performed using the standard precision mode of Glide, and the top-scoring result was selected as the docking pose.

### 4.11 Pharmacokinetic studies

In this study, C57BL/6 mice were once dosed with compound JDIC-127 at a dose of 10 mg/kg orally or 2 mg/kg intravenously. One plasma sample from each of 3 mice was collected per sampling time point. Blood samples were added to EDTA microtainers and placed on wet ice for no longer than 30 min prior to being spun at 2000×*g* for 10 min to yield at least 30 μL of plasma. Plasma samples were immediately drawn off, transferred to 1.7 mL Eppendorf tubes, and placed on dry ice/–65 °C prior to analysis. A validated LC–MS/MS method was used to determine the concentration of a tested compound in C57BL/6 mice plasma. The lower limit of detection (LLOQ) was 1 ng/mL. This study was approved by the Animal Ethics Committee of AnLing Biomed (Shenzhen) Co., Ltd. (Approval No. 2022-01-ALSHZH-05).

### 4.12 Tail suspension test

The experiment was conducted 60 min after administering compound JDIC-127, HC-070, and desipramine. C57BL/6 mice were suspended by the tail at one-third of its length using adhesive tape and hung upside down in the apparatus. The test lasted 6 min, during which immobility time and frequency, struggling time and frequency, and climbing time and frequency were recorded in the final 4 min of the test. This study was approved by the Animal Ethics Committee of Guangzhou Huateng Biomedical Technology Co., Ltd. (Approval No. HTSW221212).

### 4.13 Forced swim test

The experiment was conducted 60 min after administering compound JDIC-127, HC-070, and fluoxetine continuously for 14 days. C57BL/6 mice were placed in an apparatus filled with water maintained at 23.5 ± 0.5 °C to a depth of 10 cm. The test lasted 6 min, and immobility time, swimming time, and struggling time were recorded during the last 4 min. This study was approved by the Animal Ethics Committee of Guangzhou Huateng Biomedical Technology Co., Ltd. (Approval No. HTSW23026).

### 4.14 CCK4-induced anxiety model

All model group animals received an intraperitoneal injection of 0.3 mg/kg CCK-4 30 min prior to the behavioral test. The positive control group received an intraperitoneal injection of 1.5 mg/kg diazepam 30 min prior to the behavioral test. JDIC-127 (1, 3, 10 mg/kg) was administered orally 60 min prior to the behavioral test. This study was approved by the Animal Ethics Committee of HD Biosciences (Approval No. AUF195).

#### 4.14.1 Elevated plus maze

Prior to testing, C57BL/6J mice were acclimated to the testing environment for 60 min. At the start of the test, each mouse was placed at the central junction of the elevated plus maze facing an open arm. Behavioral data were recorded using the ANY-Maze™ video tracking system for a 5-min test session. The percentage of time spent in the open arms (open arm time %) was calculated for statistical analysis.

### 4.15 m-CPP-induced anxiety model

All model group animals received an intraperitoneal injection of 1 mg/kg m-CPP 30 min prior to the behavioral test. The positive control group received an intraperitoneal injection of 1.5 mg/kg diazepam 30 min prior to the behavioral test. JDIC-127 (1, 3, 10 mg/kg) was administered orally 60 min prior to the behavioral test. This study was approved by the Animal Ethics Committee of ICE Bioscience (Approval No. XZICE-IACUC-2024081602).

#### 4.15.1 Elevated plus maze

Prior to testing, C57BL/6J mice were acclimated to the testing environment for 60 min. At the start of the test, each mouse was placed at the central junction of the elevated plus maze facing an open arm. Behavioral data were recorded using the ANY-Maze™ video tracking system for a 5-min test session. The percentage of time spent in the open arms (open arm time %) was calculated for statistical analysis.

#### 4.15.2 Open field test

Prior to testing, C57BL/6J mice were acclimated to the testing environment for 60 min. At test initiation, each mouse was placed in the center of the open field arena. Behavioral data were recorded using the ANY-Maze™ video tracking system during a 10-min test session. The number of entries into the central zone was quantified for statistical analysis.

#### 4.15.3 Marble burying test

Prior to testing, C57BL/6J mice were acclimated to the testing environment for 60 min. Mice were tested in sanitized polycarbonate cages (30 cm × 20 cm × 15 cm) containing 5 cm of fresh bedding. Twenty glass marbles (Ø15 mm) were arranged in equidistant 5 × 4 grid prior to testing. Behavior was recorded for 30 min using the ANY-Maze™ tracking system. The number of marbles buried was counted post-test, with burial defined as >50% of the marble's volume covered by bedding material.

## Author contributions

Tong Che, Jin Zhang and Bing Xiong conceived and design the project; Tong Che, Yixiang Chen, Xinyu Cheng, Jian Li, Jin Zhang and Bing Xiong designed the experiments; Tong Che and Xiaoyun Wu designed, synthesized the compounds and collected the data; Yixiang Chen, Yuting Zhang, Weiwei Nan and Shuangyan Wan prepared cryo-EM samples; Xinyu Cheng, Jian Li and Jin Zhang carried out cryo-EM data processing, performed molecular modeling, and analyzed the data; Yixiang Chen, Han Hu and Mingxing Yang carried out calcium flux assay and electrophysiological experiments; Yixiang Chen and Xiaoqiang Yang performed *in vivo* antidepressant studies; Yinzhen Liu and Hui Liu performed the PK studies; Bo Zeng, Jian Li, Jin Zhang and Bing Xiong discussed and analyzed the results; Tong Che, Yixiang Chen, Xinyu Cheng, Jin Zhang and Bing Xiong wrote and revised the manuscript, which was then edited by all authors.

## Data availability

All data needed to evaluate the conclusions of the paper are present in the paper or the Supporting Information The cryo-EM maps have been deposited in the Electron Microscopy Data Bank (EMDB) under accession codes EMD-62784 (TRPC5/JDIC-127) and EMD-64801 (head-to-head TRPC5/JDIC-127). Coordinates have been deposited in the Protein Data Bank (PDB) under accession codes 9L3F (TRPC5/JDIC-127) and 9V6J (head-to-head TRPC5/JDIC-127). The PDB and EMDB accession codes for the published structures used in this study are 7D4Q (EMD-30576) and 6AEI (EMD-9615). All other data are available from the corresponding author upon request. Source data are provided with this paper.

## Conflicts of interest

The authors declare no conflicts of interest.

## Appendix A Supplementary data

The following is the Supporting Information to this article:
