# Supplementary material for: Structure-guided design of picomolar-level macrocyclic TRPC5 channel inhibitors with antidepressant activity
Source: Acta Pharm Sin B. 2025 Oct 27;16(1):371–86. doi: 10.1016/j.apsb.2025.10.028 (PMC12827894; doi:10.1016/j.apsb.2025.10.028)
Supplement: Multimedia component 1 [file mmc1.pdf]

Supporting Information for

Original article

Structure-guided design of picomolar-level macrocyclic TRPC5 channel inhibitors with antidepressant activity

Tong Che[a,b,†], Yixiang Chen[a,b,†], Xinyu Cheng[a,b,†], Han Hu[c], Xiaoyun Wu[c], Yuting Zhang[c], Xiaoqiang Yang[c], Yinzhen Liu[c], Hui Liu[c], Weiwei Nan[c], Shuangyan Wan[a,b], Mingxing Yang[c], Bo Zeng[d,e], Jian Li[f,*], Jin Zhang[a,b,*], Bing Xiong[g,*]

[a]*The MOE Basic Research and Innovation Center for the Targeted Therapeutics of Solid Tumors, School of Basic Medical Sciences, Jiangxi Medical College, Nanchang University, Nanchang 330031, China*

[b]*The Second Affiliated Hospital, Jiangxi Medical College, Nanchang University, Nanchang 330031, China*

[c]*Shenzhen Crystalo Biopharmaceutical Co., Ltd., Shenzhen 518118, China*

[d]*Key Laboratory of Medical Electrophysiology, Ministry of Education and Sichuan Province and Institute of Cardiovascular Research, Southwest Medical University, Luzhou 646000, China*

[e]*Department of Endocrinology, Affiliated Hospital of Southwest Medical University, Luzhou 646000, China*

[f]*College of Pharmacy, Gannan Medical University, Ganzhou 341000, China*

[g]*Department of Medicinal Chemistry, Shanghai Institute of Materia Medica, Chinese Academy of Sciences, Shanghai 201203, China*

Received 11 March 2025; received in revised form 11 July 2025; accepted 23 July 2025

[*]Corresponding authors.

E-mail addresses: rmsl_2040@163.com (Jian Li), zhangxiaokong@hotmail.com (Jin Zhang), bxiong@simm.ac.cn (Bing Xiong).

[†]These authors made equal contributions to this work.

Supporting figures

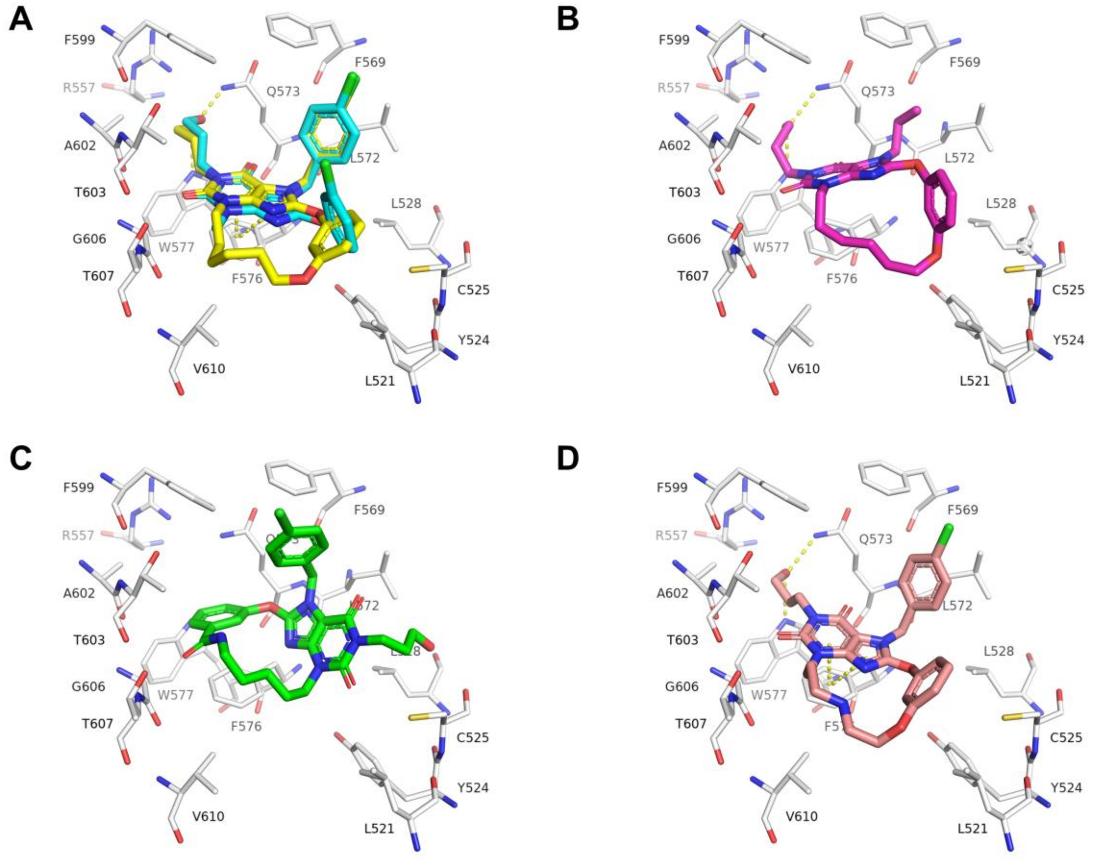

Figure S1 Proposed binding modes for macrocyclic TRPC5 inhibitors. (A) Overlay of the docking pose of compound **6** (colored in yellow) with HC-070 (colored in cyan) to the binding site of the TRPC5/HC-070 structure (PDB: 7D4Q). (B–D) The docking pose of compound **12** (colored in magenta), **17** (colored in green) and **19** (colored in orange).

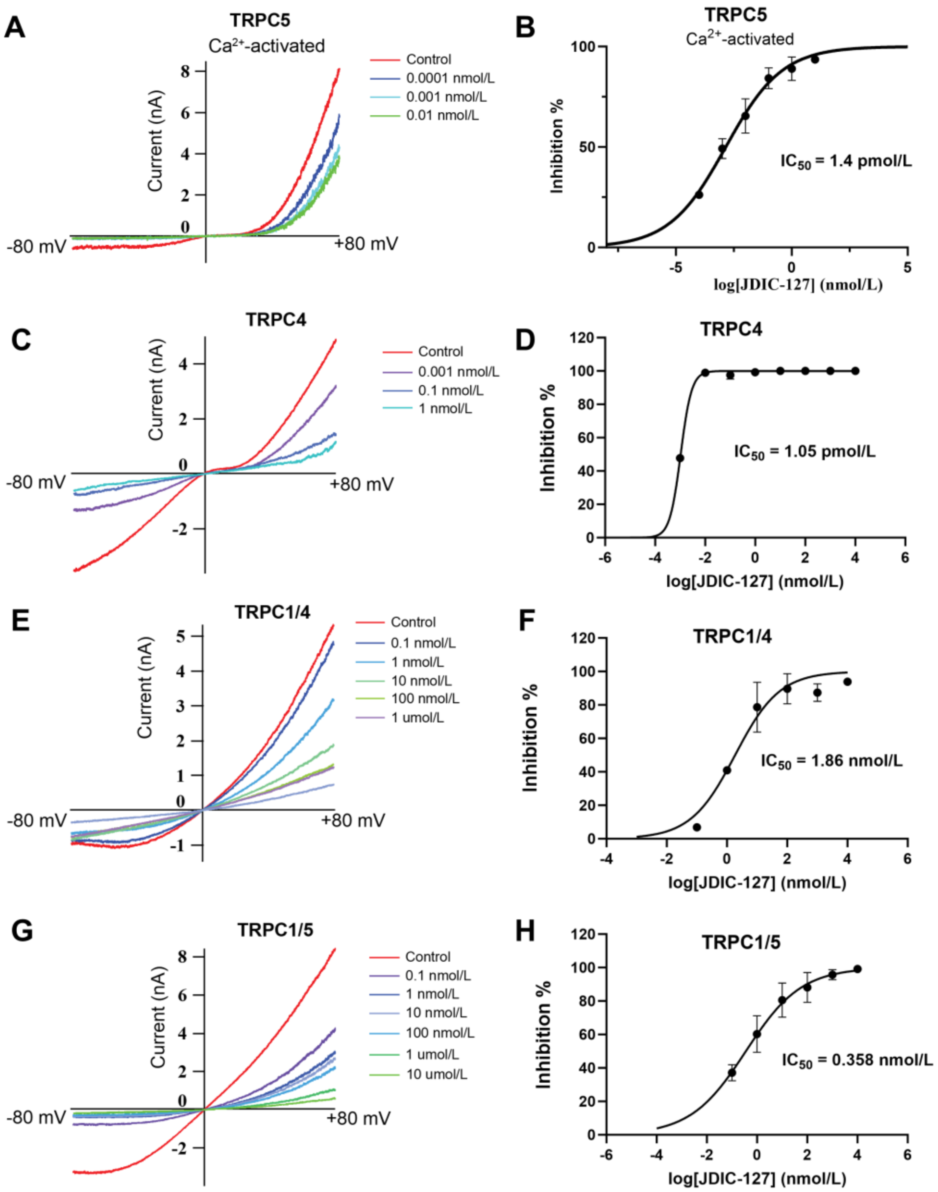

Figure S2 The potency of JDIC-127 against TRPC5, TRPC4, TRPC1/4 and TRPC1/5 channel. (A, B) The inhibition of JDIC-127 against TRPC5 by Ca^{2+}-activated. (A, B) Whole-cell patch-clamp recordings and dose-response curves of JDIC-127 against TRPC5 by Ca^{2+}-activated. (C, D) Whole-cell patch-clamp recordings and dose-response curves of JDIC-127 against TRPC4 by 100 nmol/L EA. (E, F) Whole-cell patch-clamp recordings and dose-response curves of JDIC-127 against TRPC1/4 by 100 nmol/L EA. (G, H) Whole-cell patch-clamp recordings and dose-response curves of JDIC-127 against TRPC1/5 by 100 nmol/L EA.

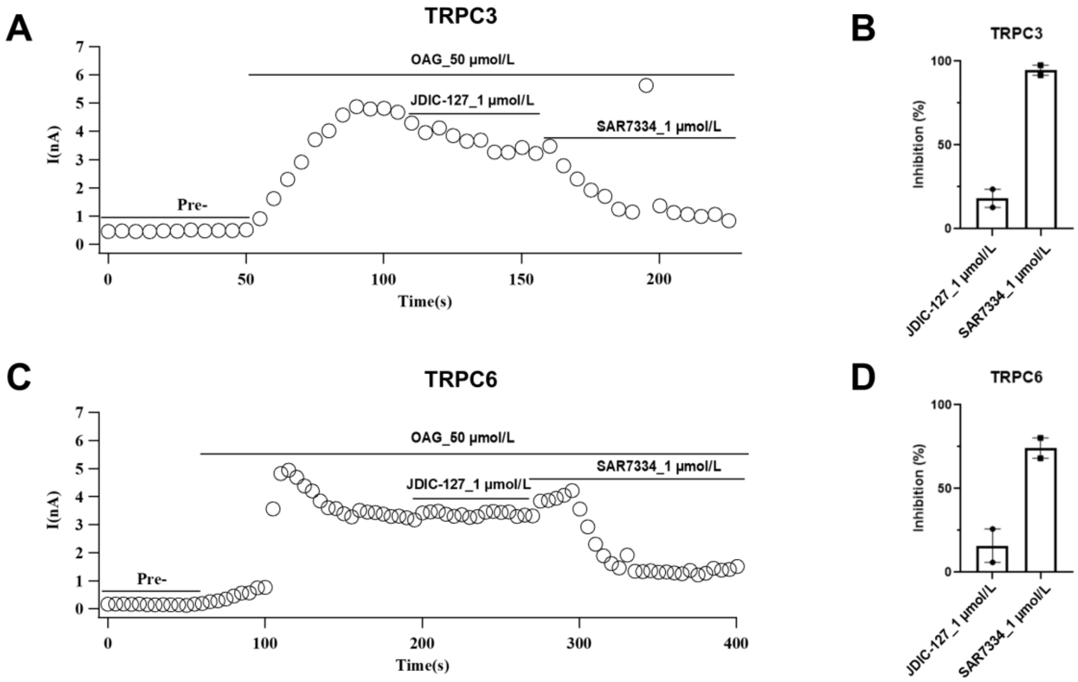

Figure S3 The selectivity of JDIC-127 toward other TRPC family. (A, B) The inhibition of JDIC-127 against TRPC3 by patch-clamp experiments. (C, D) The inhibition of JDIC-127 against TRPC6 by patch-clamp experiments.

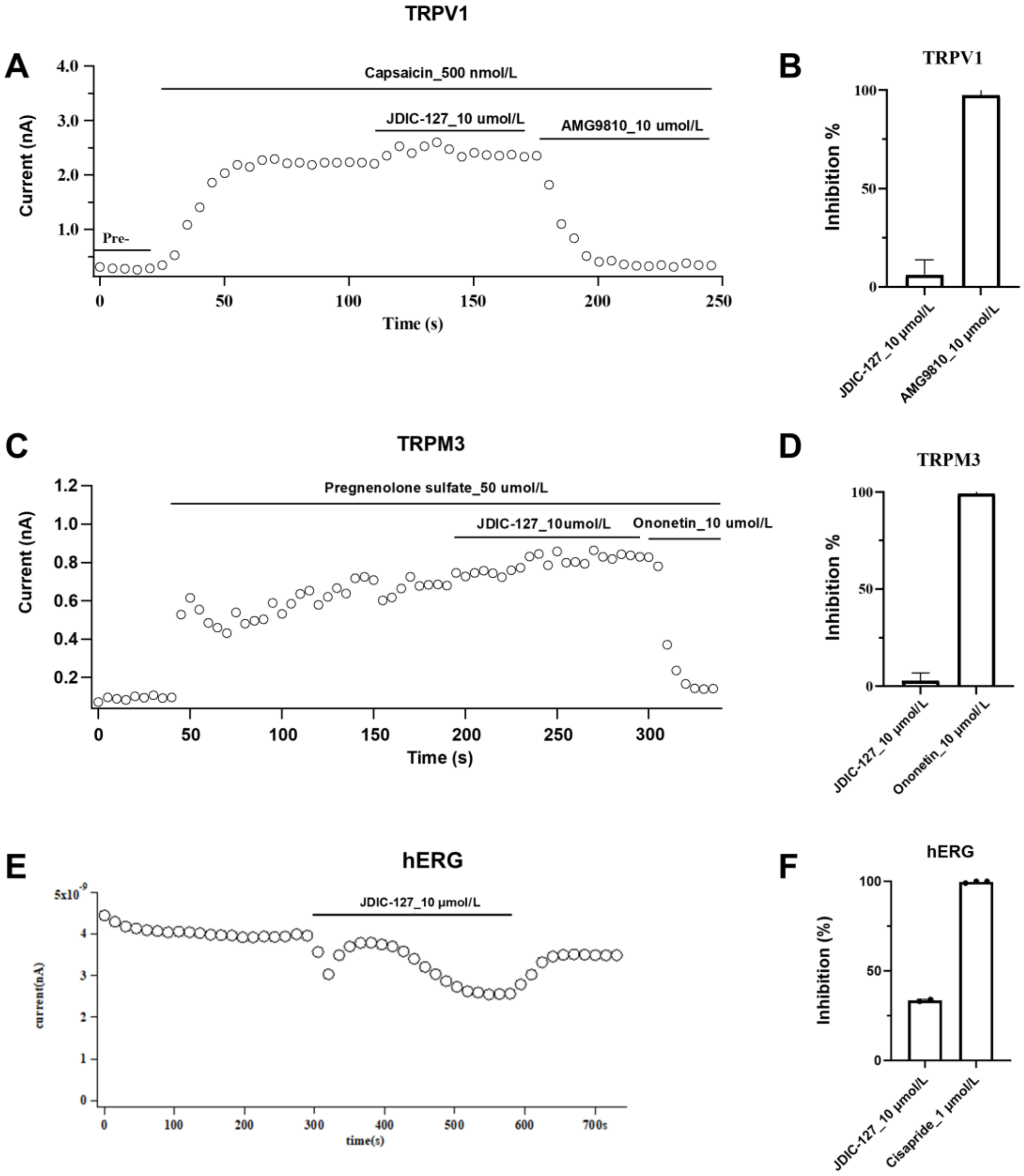

Figure S4 The selectivity of JDIC-127 toward other TRP channels and hERG. (A, B) The inhibition of JDIC-127 against TRPV1 by patch-clamp experiments. (C, D) The inhibition of JDIC-127 against TRPM3 by patch-clamp experiments. (E, F) The inhibition of JDIC-127 against hERG by patch-clamp experiments.

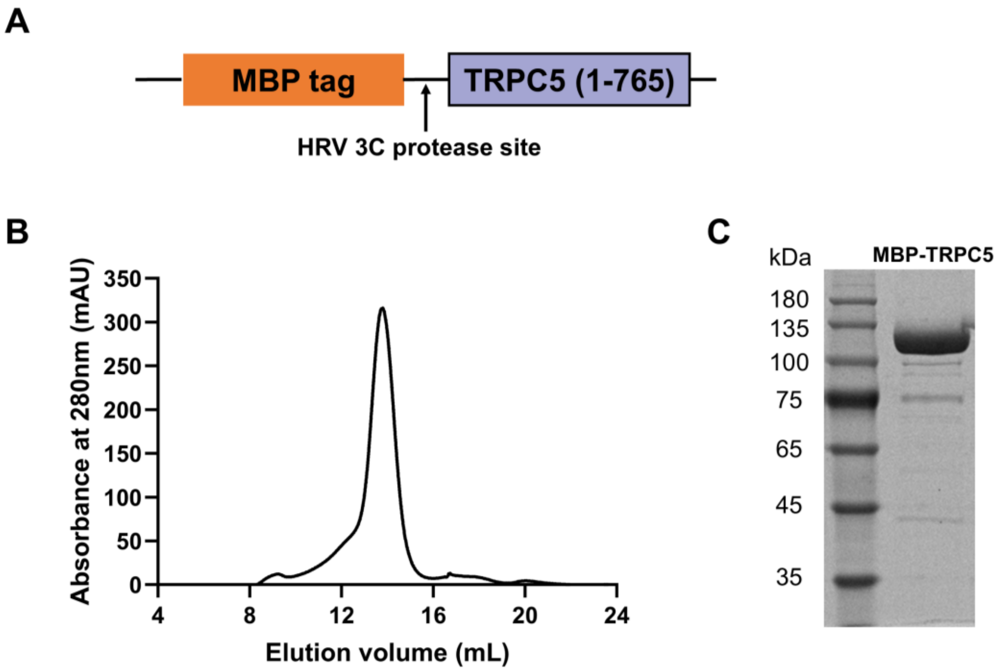

Figure S5 Purification of TRPC5. (A) A cartoon of the expressed TRPC5 construct. (B) A representative size-exclusion chromatogram profile of the purified TRPC5. (C) The purified sample of TRPC5 was stained with Coomassie blue on SDS-PAGE gel.

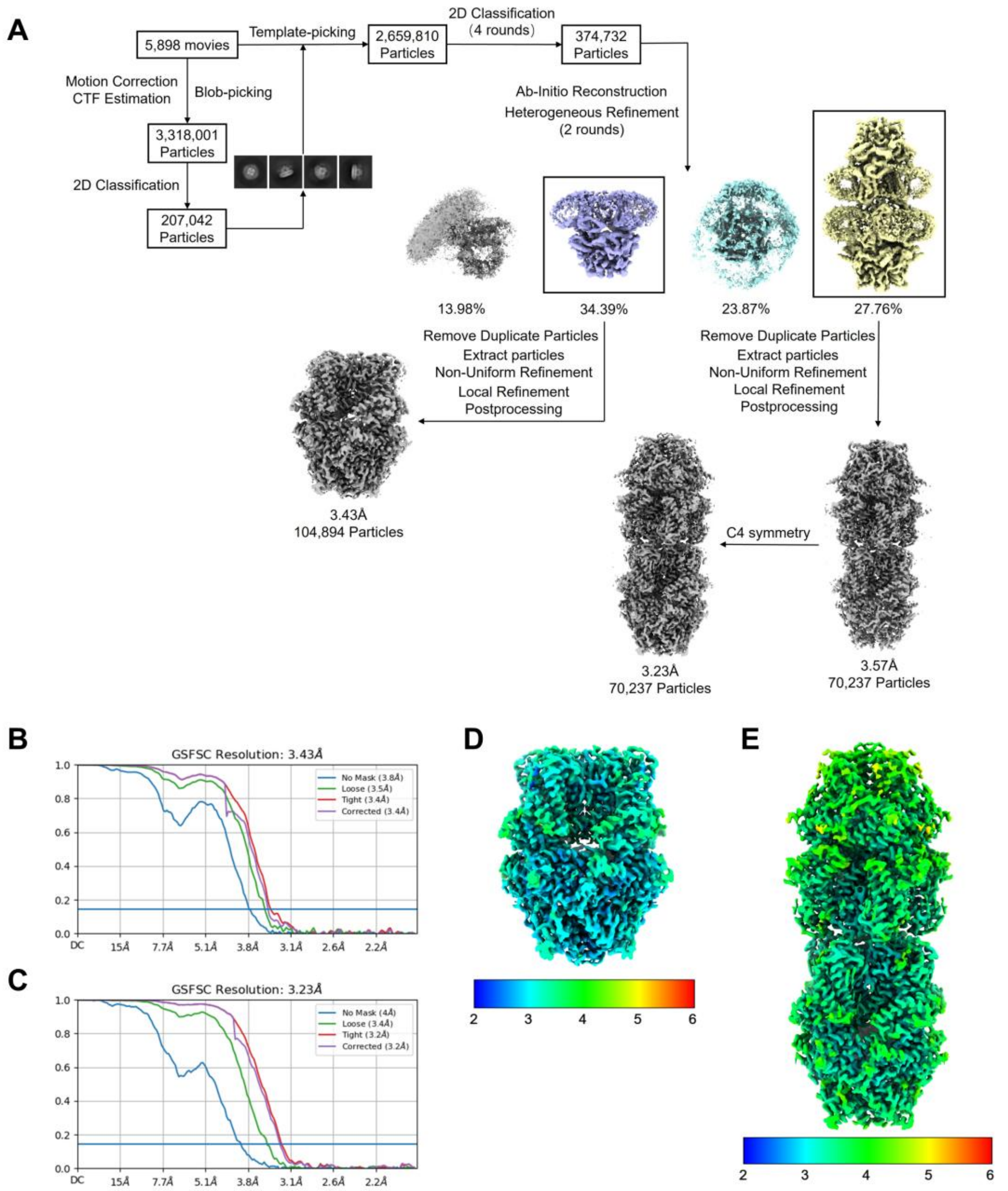

Figure S6 Cryo-EM data processing of TRPC5/JDIC-127 complex. (A) The workflow of cryo-EM data processing. A total of 5898 movies were collected for HCN1-ivabradine. Particles were autopicked in cryoSPARC, 2D and 3D classifications were conducted to remove bad particles. (B, C) Gold-Standard Fourier Shell Correlations (GSFSC) curve, the final TRPC5/JDIC-127 complex maps were determined to 3.43 Å (B), and head-to-head TRPC5/JDIC-127 complex maps were determined to 3.23 Å (C). (D, E) Local resolution presented as coloring of the TRPC5/JDIC-127 cryo-EM map (D) and head-to-head TRPC5/JDIC-127 cryo-EM map (E).

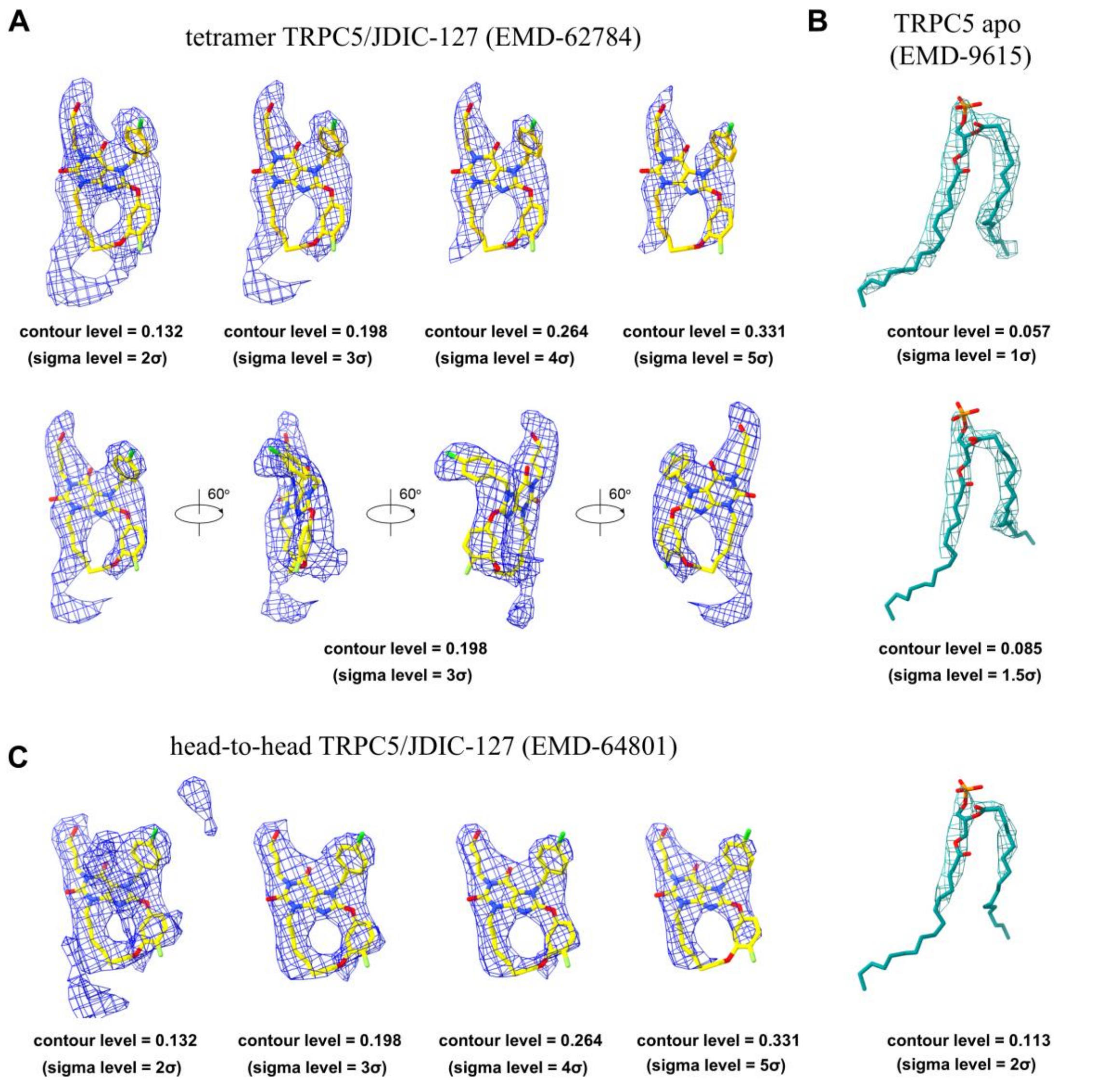

Figure S7 Cryo-EM density maps for JDIC-127. (A) Density map for JDIC-127 in the tetrameric TRPC5/JDIC-127 complex (EMD-62784), shown at different contour levels and viewing angles (map contoured at 3σ). (B) Density map for lipid in the binding pocket of apo TRPC5 (EMD-9615), shown at different contour levels. (C) Density map for JDIC-127 in the head-to-head TRPC5/JDIC-127 complex (EMD-64801), shown at different contour levels.

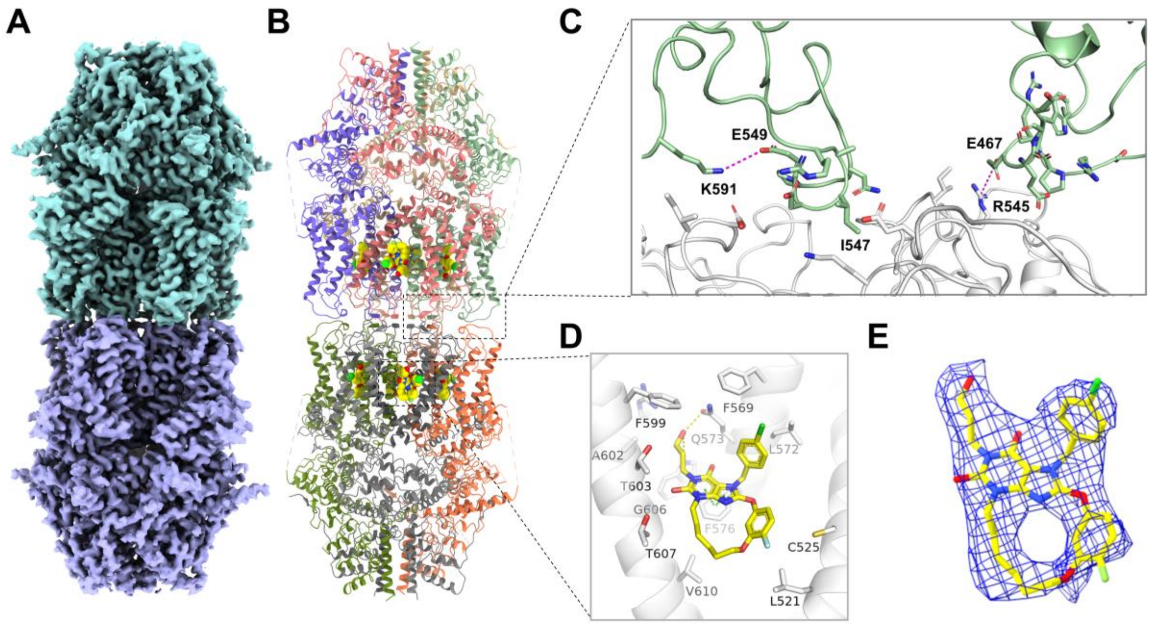

Figure S8 Structure of head-to-head TRPC5/JDIC-127 complex. (A) Cryo-EM reconstructed density maps of head-to-head TRPC5/JDIC-127 complex. (B) Ribbon diagram of head-to-head TRPC5/JDIC-127 structure is shown. (C) The detailed interaction of the head-to-head tetramer interface. (D) Detailed view of the binding site, illustrating the interactions between JDIC-127 (yellow sticks) and TRPC5 in head-to-head TRPC5/JDIC-127 complex. (E) The cryo-EM density map for JDIC-127 contoured at 3σ.

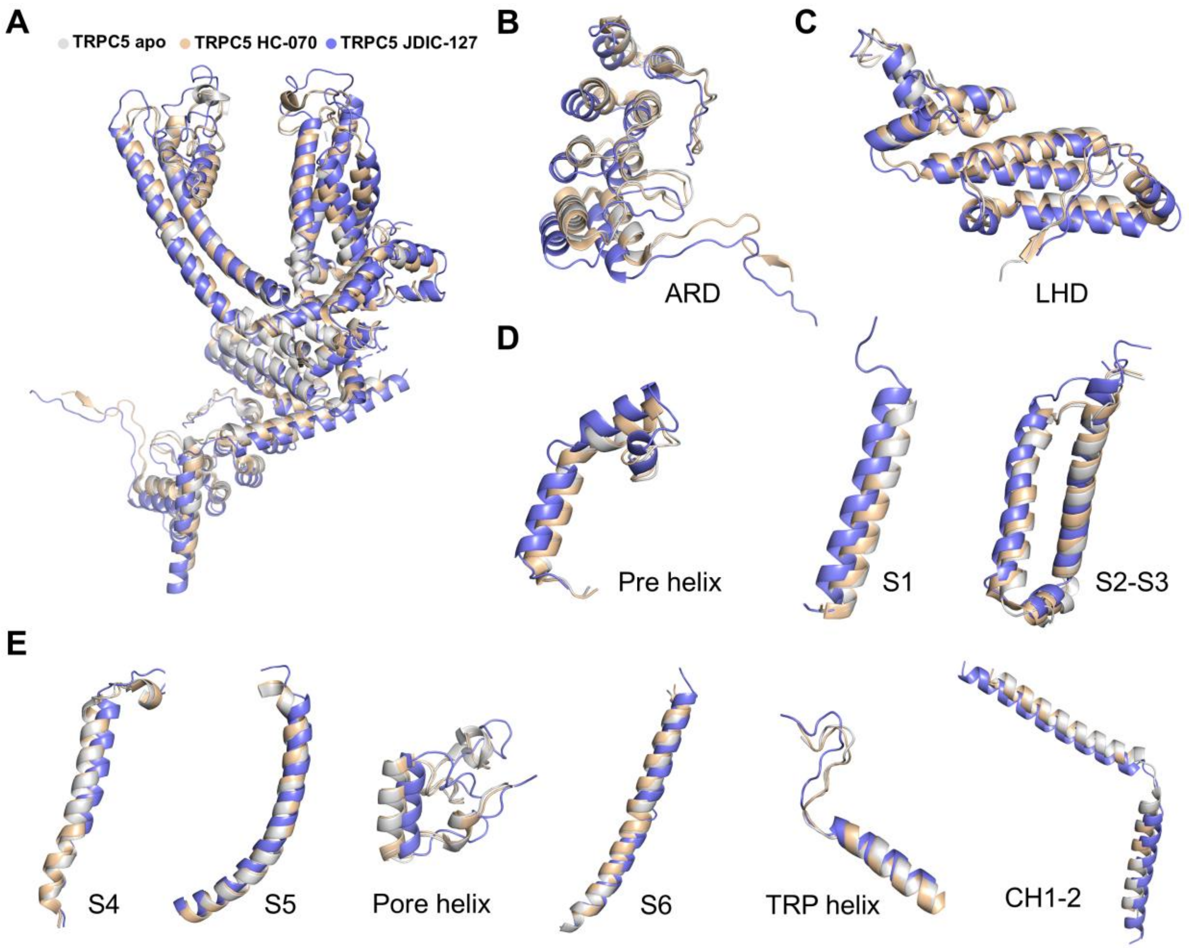

Figure S9 Structure comparison between TRPC5 apo, HC-070 bound TRPC5 and JDIC-127 bound TRPC5. (A) The overall conformational difference in one subunit between apo TRPC5 (gray, PDB: 6AEI), HC-070 bound TRPC5 (wheat, PDB: 7D4Q) and JDIC-127 bound TRPC5 (slate). (B, C) The conformational difference in ankyrin repeat domain (ARD) and linker-helix domain (LHD). (D, E) The conformational difference in pre helix, S1–S6, pore helix, TRP helix and CH1-2.

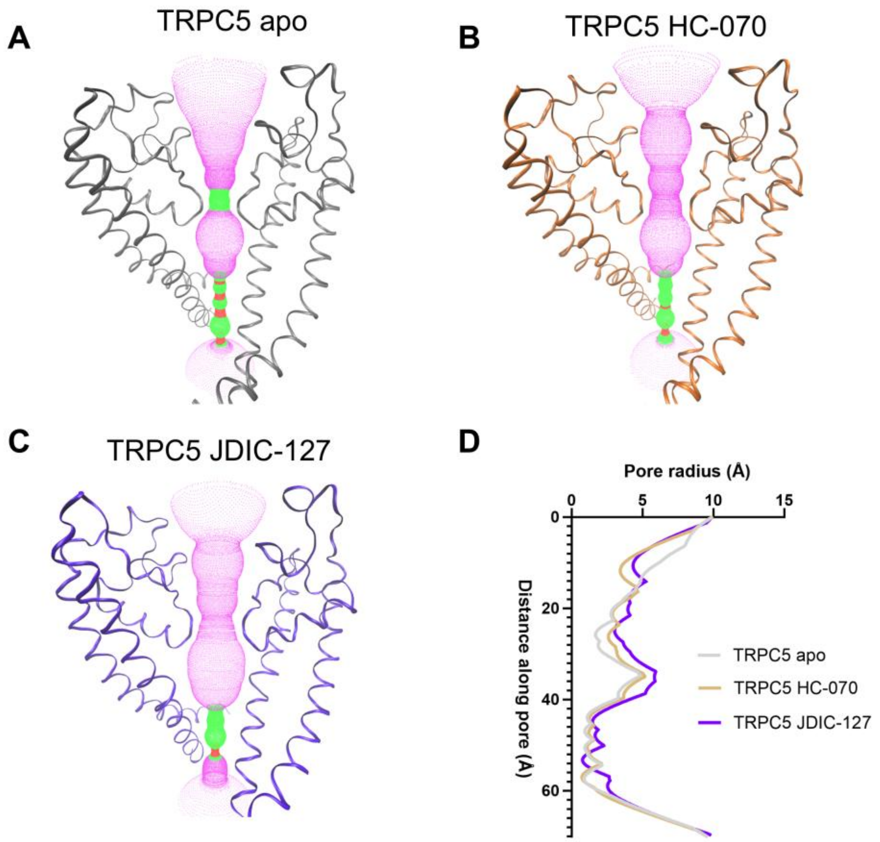

Figure S10 Ion conduction pathway comparison between TRPC5 apo, HC-070 bound TRPC5 and JDIC-127 bound TRPC5. (A) Ribbon representation of pore of TRPC5 apo (gray, PDB: 6AEI). (B) Ribbon representation of pore of TRPC5 HC-070 (wheat, PDB: 7D4Q). (C) Ribbon representation of pore of TRPC5 JDIC-127 (slate). (D) Pore radius along the central axis.

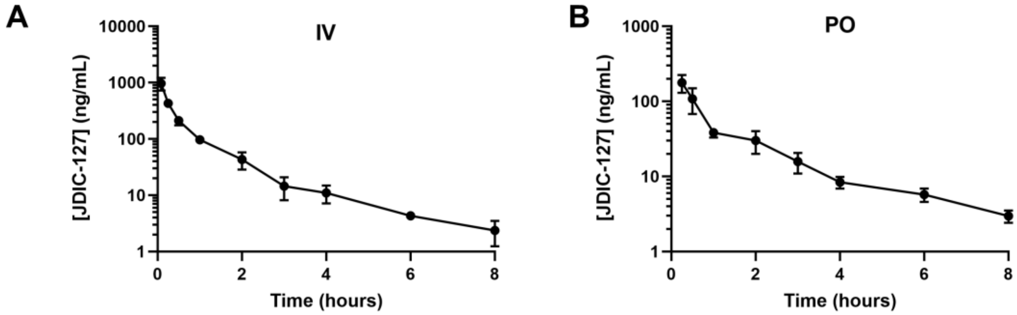

Figure S11 Pharmacokinetic properties (PK) of JDIC-127. (A) PK profiles of JDIC-127 after intravenous administration in C57BL/6 mice. (B) PK profiles of JDIC-127 after oral administration in C57BL/6 mice.

Supporting tables

Table S1 The selectivity of JDIC-127 against safety-related targets, including GPCRs, nuclear receptors, enzymes and transporters.

Class	Target	Agonist			Antagonist		
		JDIC-127 @10 μmol/L	Reference Compound	EC_{50} (nmol/L)	JDIC-127 @10 μmol/L	Reference Compound	IC_{50} (nmol/L)
GPCR	M1	–0.83%	Acetylcholine	56.58	0.22%	Atropine	5.436
	M2	2.82%	Acetylcholine	149	–0.26%	Atropine	5.512
	M3	0.99%	Acetylcholine	16.15	–1.46%	Atropine	5.173
	$5\text{-}HT_{1A}$	–1.59%	Serotonin	12.86	–6.70%	WAY-100635	0.454
	$5\text{-}HT_{1B}$	10.95%	RU 24969	4.249	–14.65%	SB-224289	0.6348
	$5\text{-}HT_{2A}$	3.92%	Serotonin	10.8	5.24%	Risperidone	0.6832
	$5\text{-}HT_{2B}$	0.67%	Serotonin	13.32	–0.45%	RS-127445	0.9722
	A2A	–1.55%	NECA	19.01	–2.78%	ZM241385	251.9
	α1A	0.76%	Epinephrine	4.02	–3.05%	Prazosin	4.692
	α2A	–1.14%	Epinephrine	1.216	–8.62%	Yohimbine	28.01
	β1	–1.87%	Isoprenaline	0.07325	1.84%	Propranolol	8.449
	β2	–1.53%	Isoprenaline	0.4256	19.63%	ICI 118551	0.3423
	CB1	33.32%	CP55940	0.148	–9.83%	Rimonabant	3.211
	CB2	26.01%	CP55940	0.2215	–0.84%	SR144528	30.43
	CCK1	–3.36%	CCK-8	0.26	6.16%	Loxiglumide	352.9
	D1	–1.54%	Dopamine	2.462	8.89%	SCH23390	1.497
	D2	–9.18%	Dopamine	3.148	–1.55%	Amisulpride	1.718
	ETA	1.45%	Endothelin 2	1.891	2.63%	Macitentan	3.292
	H1	3.21%	Histamine	16.94	–3.39%	Clemastine	5.601
	H2	–6.71%	Histamine	4.036	5.60%	Nizatidine	111.6
	μOR	0.96%	Endomorphin 1	7.166	–16.93%	Naloxone	4.413
	δOR	7.81%	SNC 80	0.3412	–21.62%	Naloxone	46.15
	κOR	0.96%	Endomorphin 1	7.166	–16.93%	Naloxone	4.413
	V1a	–7.01%	Argipressin	0.2507	–0.83%	Conivaptan	16.97
Nuclear receptors	GR	0.05%	DXMS	2.78	4.73%	Mifepristone	14.1
	AR	0.06%	DHT	0.2443	–7.02%	Enzalutamide	270.3
Enzyme	COX1	N/A	N/A	N/A	–5.92%	SC-560	70.51
	COX2	N/A	N/A	N/A	1.38%	Valdecoxib	19.04
	MAO A	N/A	N/A	N/A	15.50%	Clorgyline	1.59
	PDE3 A	N/A	N/A	N/A	2.27%	Cilostamide	28.47
	PDE4 D2	N/A	N/A	N/A	11.36%	ML-030	2.26
	ACHE	N/A	N/A	N/A	0.88%	Donepezil	25.54
	LCK	N/A	N/A	N/A	29.39%	TG 100572	0.76
Transporter	5-HTT	N/A	N/A	N/A	–31.82%	Centanafadine	126.2
	DAT	N/A	N/A	N/A	–57.57%	Centanafadine	143.9
	NET	N/A	N/A	N/A	–5.84%	Centanafadine	139

Table S2 The selectivity of JDIC-127 against safety-related targets of ion channels.

Class	Target	Agonist			Antagonist		
		JDIC-127 @ 5 μmol/L	Reference Compounds	EC_{50} (nmol/L)	JDIC-127 @5 μmol/L	Reference Compounds	IC_{50}(nmol/L) or % Inh
Ion Channels	5-HT_{3A}	0.39%	Serotonin	366.9	17.73%	Ondansetron	1.191
	Nav1.5	N/A	N/A	N/A	2.98%	TTX	96.23%
	I_{Ks}	N/A	N/A	N/A	22.77%	293B	90.96%
	Cav1.2	N/A	N/A	N/A	20.23%	Nifedipine	89.04%
	hERG	N/A	N/A	N/A	19.40%	Cisapride	21.11
	nAChRα4β2	0.97%	Acetylcholine	948.3	2.92%	Adiphenine	547.1
	$GABA_A$	0.74%	GABA	1648	26.35%	PTX	6758
	NR1/NR2B	1.53%	Glycine	132.1	17.19%	D-AP5	87.44%

Table S3 Cryo-EM data collection, refinement and validation statistics.

Structure	TRRPC5/JDIC-127	Head-to-Head TRRPC5/JDIC-127
EMDB accession code	EMD-62784	EMD-64801
PDB accession code	9L3F	9V6J
**Data collection and processing**		
Magnification	130,000	130,00
Voltage (kV)	300	300
Electron exposure (e–/Å^2)	50.54	50.54
Defocus range (μm)	–1.0 ~ –2.0	–1.0 ~ –2.0
Pixel size (Å)	0.96	0.96
Symmetry imposed	*C4*	*C4*
Initial particle images (#)	2,658,910	2,658,910
Final particle images (#)	104,894	70,237
Map resolution (Å)	3.43	3.23
FSC threshold	0.143	0.143
**Refinement**		
Initial model used (PDB code)	7D4Q	7D4Q
Model resolution (Å)	2.74	2.74
FSC threshold	0.143	0.143
**Model composition**		
Non-hydrogen atoms	22,652	45,304
Protein residues	2752	5504
Ligands	12	24
***B* factors (Å^2)**		
Protein	70.12	68.15
Ligand	143.70	141.70
r.m.s. deviations		
Bond lengths (Å)	0.003	0.007
Bond angles (°)	0.574	1.090
**Validation**		
MolProbity score	1.23	1.25
Clashscore	2.94	3.07
Poor rotamers (%)	0.33	0.77
Ramachandran plot		
Favored (%)	97.06	97.13
Allowed (%)	2.94	2.87
Disallowed (%)	0.00	0.00

3. Chemical synthesis section

3.1. General information of chemical synthesis

All starting materials, solvents, and reagents were used directly as obtained commercially without further purification unless otherwise noted. ^1H NMR spectra were recorded using $CDCl_3$, DMSO-d_6, or MeOD-d_4 on a Bruker Avance 400 MHz spectrometer. Chemical shifts are reported in parts per million referenced with respect to residual solvent (MeOD-d_4) 3.31 ppm, (DMSO-d_6) 2.50 ppm, and ($CDCl_3$) 7.26 ppm. Coupling constants (*J*) are expressed in hertz (Hz). Chemical shifts (δ) of NMR are reported in parts per million (ppm) units. The first-order peak patterns are indicated as s (singlet) and d (doublet). All compounds submitted for testing were confirmed to be > 95.0% purity by HPLC traces.

*3.2. General procedures for the synthesis of compounds **5**–**11***

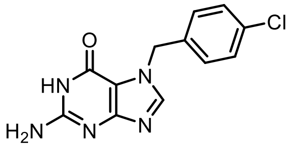

2-Amino-7-(4-chlorobenzyl)-1,7-dihydro-6*H*-purin-6-one (26). To a solution of 2-amino-9-((2*R*,3*R*,4*S*,5*R*)-3,4-dihydroxy-5-(hydroxymethyl)tetrahydrofuran-2-yl)-1,9-dihydro-6*H*-purin-6-one (**25**, 10.0 g, 35.3 mmol, 1.00 eq.) in DMSO (70.0 mL) was added 1-chloro-4-(chloromethyl)benzene (7.11 g, 44.1 mmol, 1.25 eq.). The mixture was stirred at 50 ℃ for 16 h. Then the mixture was stirred with HCl (1 mol/L, 5 V) at 70 ℃ for 3 h under N_2 atmosphere. Cooled the reaction mixture to room temperature, filtered the mixture and the filter cake was washed with H_2O 200 mL, EtOH 50 mL. Concentrated the filter cake to give compound **26** as a white solid (3.50 g, 36.0% yield). ^1H NMR (400 MHz, DMSO-d_6): δ 11.60 (s, 1H), 10.89 (s, 1H), 8.14 (s, 1H), 7.44–7.38 (m, 2H), 7.37–7.32 (m, 2H), 5.40 (s, 2H). LC–MS: 276.1 ($[M+H]^+$).

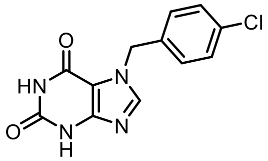

7-(4-Chlorobenzyl)-3,7-dihydro-1*H*-purine-2,6-dione (27). To a solution of **26** (3.5 g, 12.70 mmol, 1.00 eq.) in AcOH (20 mL), $NaNO_2$ (964 mg, 13.97 mmol, 1.10 eq.) in H_2O (4.00 mL) was dropwise to the mixture at 50 ℃, the mixture was stirred at 50 ℃ for 3 h.

Added 100 mL H_2O to the reaction mixture, filtered the mixture and the filter cake was concentrated to give compound **27** as a white solid (1.20 g, 34.2% yield). ^{1}H NMR (400 MHz, DMSO-d_6): δ 11.60 (s, 1H), 10.89 (s, 1H), 8.14 (s, 1H), 7.44–7.38 (m, 2H), 7.37–7.32 (m, 2H), 5.40 (s, 2H). LC–MS: 277.0 ([M+H]$^+$).

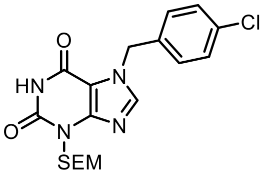

7-(4-Chlorobenzyl)-3-((2-(trimethylsilyl)ethoxy)methyl)-3,7-dihydro-1*H*-purine-2,6-dione (28). To a solution of **27** (100 mg, 361 μmol, 1.00 eq.) in DMF (3.00 mL) was added DBU (55.0 mg, 361 μmol, 54.5 μL, 1.00 eq.) and SEM-Cl (60.3 mg, 361 μmol, 64.0 μL, 1.00 eq.). The mixture was stirred at rt for 16 h. Concentrated the reaction mixture to get the crude product. The residue was purified by prep-TLC (Petroleum ether/Ethyl acetate = 3/1) to give compound **28** as a white solid (20.0 mg, 13.6% yield). ^{1}H NMR (400 MHz, $CDCl_3$): δ 8.42 (s, 1H), 7.61 (s, 1H), 7.37–7.32 (m, 2H), 7.31–7.27 (m, 2H), 5.50 (s, 2H), 5.42 (s, 2H), 3.72 (dd, J = 7.8, 9.0 Hz, 2H), 1.04–0.91 (m, 2H), 0.03–0.01 (m, 9H). LC–MS: 407.1 ([M+H]$^+$).

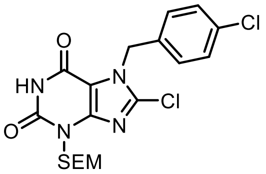

8-Chloro-7-(4-chlorobenzyl)-3-((2-(trimethylsilyl)ethoxy)methyl)-3,7-dihydro-1*H*-purine-2,6-dione (29). To a solution of **28** (2.50 g, 6.14 mmol, 1.00 eq.) in THF (15.0 mL) was added NCS (1.23 g, 9.22 mmol, 1.50 eq.). The mixture was stirred at rt for 2 h. The reaction mixture was quenched by addition H_2O 50.0 mL at rt, and then extracted with EtOAc 40.0 mL (20.0 mL × 2). The combined organic layers concentrated under reduced pressure to give compound **29** as a yellow oil (3.00 g, crude). LC–MS: 442.1 ([M+H]$^+$).

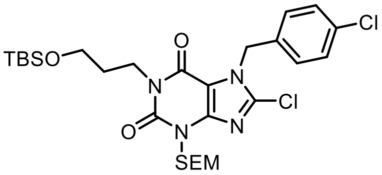

1-(3-((*tert*-Butyldimethylsilyl)oxy)propyl)-8-chloro-7-(4-chlorobenzyl)-3-((2-(trimethylsilyl)ethoxy) methyl)-3,7-dihydro-1*H*-purine-2,6-dione (30). To a solution of

29 (5.21 g, 11.8 mmol, 1.00 eq.) in DMF (14.0 mL) was added K_2CO_3 (3.26 g, 23.6 mmol, 2.00 eq.) and (3-bromopropoxy)(*tert*-butyl)dimethylsilane (4.48 g, 17.71 mmol, 1.50 eq.). The mixture was stirred at 70 ℃ for 16 h. The reaction mixture was quenched by addition H_2O 50 mL at rt, and then extracted with EtOAc 40 mL (20 mL × 2). The combined organic layers concentrated under reduced pressure to give compound **30** as a yellow oil (7.24 g, 99.9% yield). LC–MS: 612.2 ($[M+H]^+$).

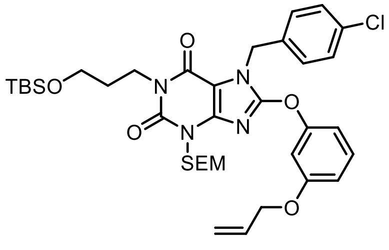

8-(3-(Allyloxy)phenoxy)-1-(3-((*tert*-butyldimethylsilyl)oxy)propyl)-7-(4-chlorobenzyl)-3-((2-(trimethylsilyl)ethoxy)methyl)-3,7-dihydro-1*H*-purine-2,6-dione (31a). To a solution of **30** (7.24 g, 11.8 mmol, 1.00 eq.) in DMF (50.0 mL) was added K_2CO_3 (3.26 g, 23.6 mmol, 2.00 eq.) and 3-(allyloxy)phenol (1.95 g, 13.0 mmol, 1.10 eq.). The mixture was stirred at 80 ℃ for 16 h. The reaction mixture was quenched by addition H_2O 200 mL at rt, and then extracted with EtOAc 180 mL (60.0 mL × 3). The combined organic layers concentrated under reduced pressure to give a residue. The residue was purified by silica gel column chromatography (Petroleum ether/Ethyl acetate=100/1 to 5/1) to give compound **31a** as a yellow oil (5.0 g, 45.5% yield). LC–MS: 727.6 ($[M+H]^+$).

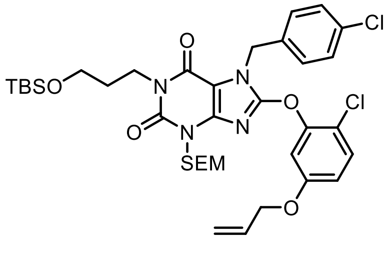

8-(5-(Allyloxy)-2-chlorophenoxy)-1-(3-((*tert*-butyldimethylsilyl)oxy)propyl)-7-(4-chlorobenzyl)-3-((2-(trimethylsilyl)ethoxy)methyl)-3,7-dihydro-1*H*-purine-2,6-dione (31b). Compound **31b** was prepared using a similar procedure to that described above for the synthesis of **31a** by **30** and 5-(allyloxy)-2-chlorophenol. The residue was purified by silica gel column chromatography (Petroleum ether/Ethyl acetate=100/1 to 5/1) to give compound **31b** as a yellow oil (2.0 g, 80.6% yield). LC–MS: 762.2 ($[M+H]^+$).

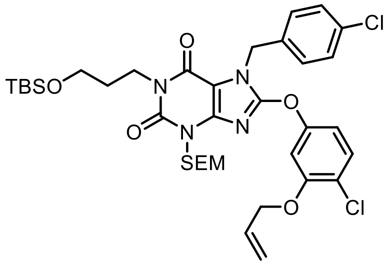

8-(3-(Allyloxy)-4-chlorophenoxy)-1-(3-((*tert*-butyldimethylsilyl)oxy)propyl)-7-(4-chlorobenzyl)-3-((2-(trimethylsilyl)ethoxy)methyl)-3,7-dihydro-1*H*-purine-2,6-dione (31c). Compound **31c** was prepared using a similar procedure to that described above for the synthesis of **31a** by **30** and 3-(allyloxy)-4-chlorophenol. The residue was purified by silica gel column chromatography (Petroleum ether/Ethyl acetate=100/1 to 5/1) to give compound **31c** as a yellow oil (3.0 g, 48.5% yield). LC–MS: 761.3 ($[M+H]^+$).

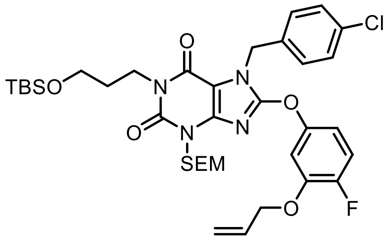

8-(3-(Allyloxy)-4-fluorophenoxy)-1-(3-((*tert*-butyldimethylsilyl)oxy)propyl)-7-(4-chlorobenzyl)-3-((2-(trimethylsilyl)ethoxy)methyl)-3,7-dihydro-1*H*-purine-2,6-dione (31d). Compound **31d** was prepared using a similar procedure to that described above for the synthesis of **31a** by **30** and 3-(allyloxy)-4-fluorophenol. The residue was purified by silica gel column chromatography (Petroleum ether/Ethyl acetate=100/1 to 5/1) to give compound **31d** as a yellow oil (2.6 g, 87.2% yield). LC–MS: 745.2 ($[M+H]^+$).

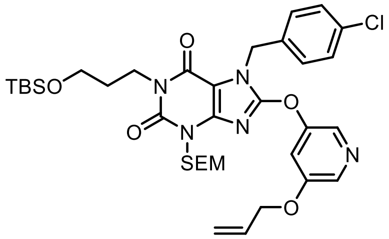

8-((5-(Allyloxy)pyridin-3-yl)oxy)-1-(3-((*tert*-butyldimethylsilyl)oxy)propyl)-7-(4-chlorobenzyl)-3-((2-(trimethylsilyl)ethoxy)methyl)-3,7-dihydro-1*H*-purine-2,6-dione (31e). Compound **31e** was prepared using a similar procedure to that described above for the synthesis of **31a** by **30** and 5-(allyloxy)pyridin-3-ol. The residue was purified by silica gel column chromatography (Petroleum ether/Ethyl acetate=50/1 to 2/1) to give compound

31e as a brown oil (1.7 g, 89.5% yield). LC–MS: 728.5 ([M+H]$^+$).

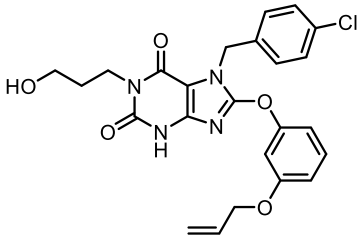

8-(3-(Allyloxy)phenoxy)-7-(4-chlorobenzyl)-1-(3-hydroxypropyl)-3,7-dihydro-1*H*-purine-2,6-dione (32a). To a solution of **31a** (3.00 g, 4.12 mmol, 1.00 eq.) in EtOH (20.0 mL) was added HCl (12.0 mol/L, 3.00 mL). The mixture was stirred at 80 ℃ for 16 h. Concentrated the reaction mixture to get the crude product. The residue was purified by silica gel column chromatography (Petroleum ether/Ethyl acetate = 50/1 to 2/1) to give compound **32a** as a yellow solid (1.00 g, 50.2% yield). LC–MS: 483.1 ([M+H]$^+$).

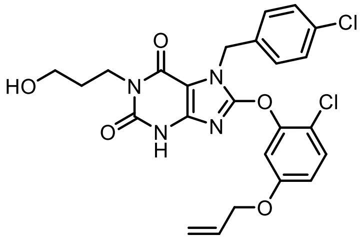

8-(5-(Allyloxy)-2-chlorophenoxy)-7-(4-chlorobenzyl)-1-(3-hydroxypropyl)-3,7-dihydro-1*H*-purine-2,6-dione (32b). Compound **32b** was prepared using a similar procedure to that described above for the synthesis of **32a** by **31b**. The residue was purified by column chromatography (SiO_2, Petroleum ether/Ethyl acetate=50/1 to 2/1) to give compound **32b** as a white solid (1.1 g, 54.0% yield). LC–MS: 517.0 ([M+H]$^+$).

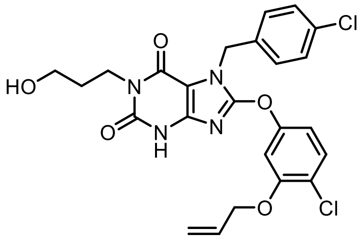

8-(3-(Allyloxy)-4-chlorophenoxy)-7-(4-chlorobenzyl)-1-(3-hydroxypropyl)-3,7-dihydro-1*H*-purine-2,6-dione (32c). Compound **32c** was prepared using a similar procedure to that described above for the synthesis of **32a** by **31c**. The residue was purified by column chromatography (SiO_2, Petroleum ether/Ethyl acetate=100/1 to 2/1) to give compound **32c** as a white solid (0.9 g, 44.2% yield). LC–MS: 517.1 ([M+H]$^+$).

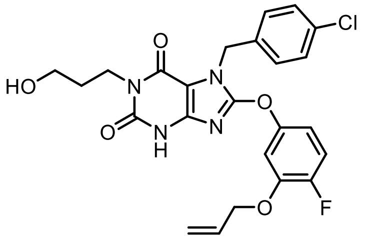

8-(3-(Allyloxy)-4-fluorophenoxy)-7-(4-chlorobenzyl)-1-(3-hydroxypropyl)-3,7-dihydro-1*H*-purine-2,6-dione (32d). Compound **32d** was prepared using a similar procedure to that described above for the synthesis of **32a** by **31d**. The residue was purified by column chromatography (Petroleum ether/Ethyl acetate= 100/1 to 1/2) to give compound **32d** as a white solid (0.8 g, 45.8% yield). LC–MS: 501.1 ($[M+H]^+$).

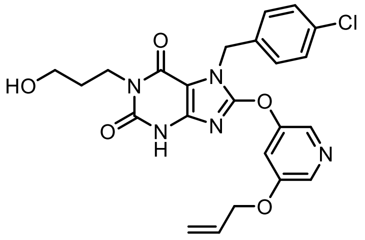

8-((5-(Allyloxy)pyridin-3-yl)oxy)-7-(4-chlorobenzyl)-1-(3-hydroxypropyl)-3,7-dihydro-1*H*-purine-2,6-dione (32e). Compound **32e** was prepared using a similar procedure to that described above for the synthesis of **32a** by **31e**. The residue was purified by column chromatography (Petroleum ether/Ethyl acetate= 10/1) to give compound **32e** as a colorless oil (0.5 g, 62.7% yield). LC–MS: 484.2 ($[M+H]^+$).

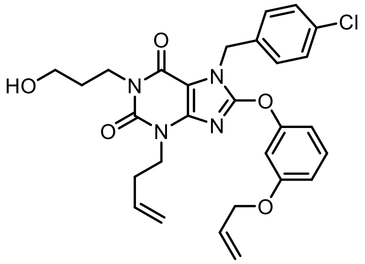

8-(3-(Allyloxy)phenoxy)-3-(but-3-en-1-yl)-7-(4-chlorobenzyl)-1-(3-hydroxypropyl)-3,7-dihydro-1*H*-purine-2,6-dione (33a). To a solution of **32a** (100 mg, 207 μmol, 1.00 eq.) in DMF (2 mL) was added 4-bromobut-1-ene (41.9 mg, 311 μmol, 31.5 μL, 1.50 eq.), Cs_2CO_3 (135 mg, 414 μmol, 2.00 eq.). The mixture was stirred at 100 ℃ for 3 h. The reaction mixture was quenched by addition H_2O 20 mL at rt, and then extracted with EtOAc 10.0 mL. The combined organic layers were concentrated under reduced pressure to give a residue. The residue was purified by prep-TLC (Petroleum ether/Ethyl acetate=1/1) to

give compound **33a** as a colorless oil (50.0 mg, 45.0% yield). LC–MS: 538.0 ([M+H]$^+$).

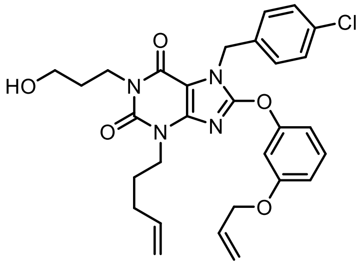

8-(3-(Allyloxy)phenoxy)-7-(4-chlorobenzyl)-1-(3-hydroxypropyl)-3-(pent-4-en-1-yl)-3,7-dihydro-1*H*-purine-2,6-dione (33b). Compound **33b** was prepared using a similar procedure to that described above for the synthesis of **33a** by **32a** and 5-bromopent-1-ene. The crude product was purified by silica gel column chromatography (Petroleum ether/Ethyl acetate=1/1) to give **33b** (50.0 mg, 21.9% yield) as a colorless oil. LC–MS: 551.2 ([M+H]$^+$).

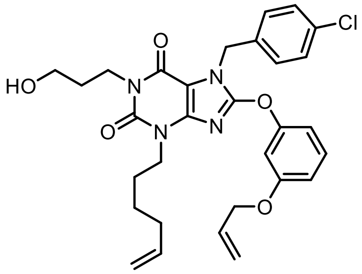

8-(3-(Allyloxy)phenoxy)-7-(4-chlorobenzyl)-3-(hex-5-en-1-yl)-1-(3-hydroxypropyl)-3,7-dihydro-1*H*-purine-2,6-dione (33c). Compound **33c** was prepared using a similar procedure to that described above for the synthesis of **33a** by **32a** and 6-bromohex-1-ene. The crude product was purified by silica gel column chromatography (Petroleum ether/Ethyl acetate=1/1) to give **33b** (120 mg, 51.3% yield) as a colorless oil. LC–MS: 565.2 ([M+H]$^+$).

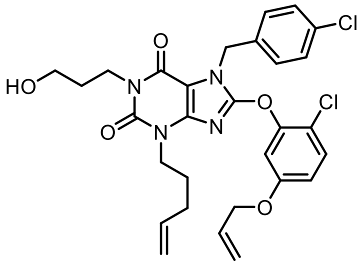

8-(5-(Allyloxy)-2-chlorophenoxy)-7-(4-chlorobenzyl)-1-(3-hydroxypropyl)-3-(pent-4-en-1-yl)-3,7-dihydro-1*H*-purine-2,6-dione (33d). Compound **33d** was prepared using a similar procedure to that described above for the synthesis of **33a** by **32b** and 5-bromopent-

1-ene. The crude product was purified by silica gel column chromatography (Petroleum ether/Ethyl acetate=3/2) to give **33d** (400 mg, 70.7% yield) as a white solid. LC–MS: 586.1 ($[M+H]^+$).

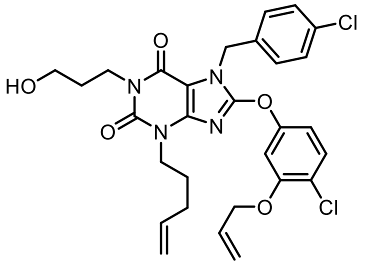

8-(3-(Allyloxy)-4-chlorophenoxy)-7-(4-chlorobenzyl)-1-(3-hydroxypropyl)-3-(pent-4-en-1-yl)-3,7-dihydro-1*H*-purine-2,6-dione (33e). Compound **33e** was prepared using a similar procedure to that described above for the synthesis of **33a** by **32c** and 5-bromopent-1-ene. The crude product was purified by silica gel column chromatography (Petroleum ether/Ethyl acetate=2/3) to give **33e** (350 mg, 68.7% yield) as a white oil. LC–MS: 585.3 ($[M+H]^+$).

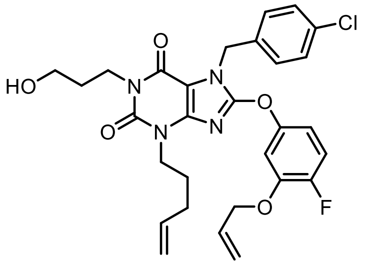

8-(3-(Allyloxy)-4-fluorophenoxy)-7-(4-chlorobenzyl)-1-(3-hydroxypropyl)-3-(pent-4-en-1-yl)-3,7-dihydro-1*H*-purine-2,6-dione (33f). Compound **33f** was prepared using a similar procedure to that described above for the synthesis of **33a** by **32d** and 5-bromopent-1-ene. The crude product was purified by silica gel column chromatography (Petroleum ether/Ethyl acetate = 2/3) to give **33f** (300 mg, 66.0% yield) as a white oil. LC–MS: 569.4 ($[M+H]^+$).

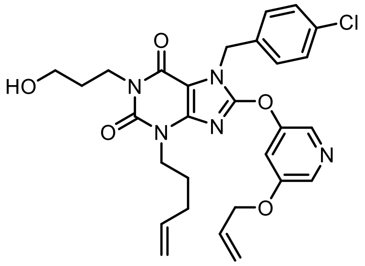

8-((5-(Allyloxy)pyridin-3-yl)oxy)-7-(4-chlorobenzyl)-1-(3-hydroxypropyl)-3-(pent-4-

en-1-yl)-3,7-dihydro-1*H*-purine-2,6-dione (33g). Compound **33g** was prepared using a similar procedure to that described above for the synthesis of **33a** by **32e** and 5-bromopent-1-ene. The crude product was purified by silica gel column chromatography (Petroleum ether/Ethyl acetate = 3/1) to give **33g** (190 mg, 42.2% yield) as a yellow oil. LC–MS: 552.8 ($[M+H]^+$).

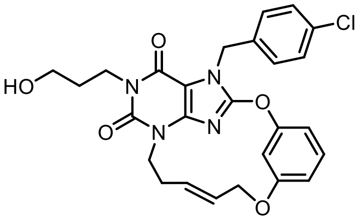

(1^8E,6*E*)-1^7-(4-Chlorobenzyl)-1^1-(3-hydroxypropyl)-1^2,1^3,1^6,1^7-tetrahydro-1^1*H*-2,4-dioxa-1(8,3)-purina-3(1,3)-benzenacyclononaphan-6-ene-1^2,1^6-dione (34a). To a solution of **33a** (20.0 mg, 37.2 μmol, 1.00 eq.) in DCM (2.00 mL) was added [1,3-bis(2,4,6-trimethylphenyl)imidazolidin-2-ylidene]-dichloro-[(2-isopropoxyphenyl)methylene]ruthenium (HG-II, 17.50 mg, 27.93 μmol, 0.1 eq.).The mixture was stirred at 40 ℃ for 16 h. Concentrated the reaction mixture to get the crude product. The residue was purified by prep-HPLC (column: Welch Ultimate C18 150 mm × 25 mm × 5 μm; mobile phase: [water (TFA)-ACN]; B%: 44%–74%, 10 min) to give **34a** as a white solid (2.42 mg, 12.6% yield). ^{1}H NMR (400 MHz, MeOD-d_4): δ 7.47 (d, J = 8.5 Hz, 2H), 7.37 (d, J = 8.4 Hz, 2H), 7.23 (t, J = 8.2 Hz, 1H), 6.90 (t, J = 2.2 Hz, 1H), 6.79 (dd, J = 1.5, 8.0 Hz, 1H), 6.72 (dd, J = 1.6, 8.4 Hz, 1H), 5.72–5.60 (m, 1H), 5.59–5.49 (m, 1H), 5.44 (s, 2H), 4.55 (d, J = 6.8 Hz, 2H), 4.16–4.05 (m, 4H), 3.62 (t, J = 6.4 Hz, 2H), 2.49–2.38 (m, 2H), 1.88 (quin, J = 6.7 Hz, 2H). LC–MS: 509.3 ($[M+H]^+$).

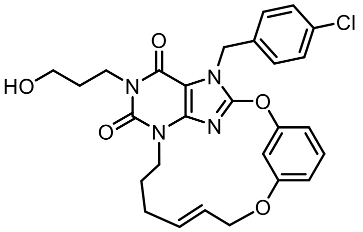

(1^8E,6*E*)-1^7-(4-Chlorobenzyl)-1^1-(3-hydroxypropyl)-1^2,1^3,1^6,1^7-tetrahydro-1^1*H*-2,4-dioxa-1(8,3)-purina-3(1,3)-benzenacyclodecaphan-6-ene-1^2,1^6-dione (34b). Compound **34b** was prepared using a similar procedure to that described above for the synthesis of **34a** by **33b**. The residue was purified by prep-HPLC (column: Welch Ultimate C18 150

mm × 25 mm × 5 μm; mobile phase: [water (TFA)-ACN]; B%: 50%–80%, 10 min) to give **34b** as a white solid (7.86 mg, 16.2% yield). ^{1}H NMR (400 MHz, MeOD-d_4): δ 7.83 (s, 1H), 7.44 (s, 4H), 7.27 (t, J = 8.2 Hz, 1H), 6.86-6.76 (m, 2H), 6.09-5.93 (m, 1H), 5.66 (dt, J = 15.6, 5.7 Hz, 1H), 5.42 (s, 2H), 4.70 (br d, J = 5.3 Hz, 2H), 4.45 (t, J = 5.2 Hz, 1H), 4.00–3.86 (m, 4H), 3.43 (q, J = 6.1 Hz, 2H), 2.07 (br s, 2H), 1.78-1.64 (m, 4H). LC–MS: 523.3 ([M+H]$^+$).

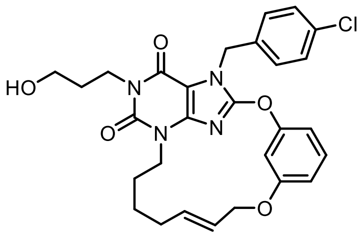

(1^8*E*,6*E*)-1^7-(4-Chlorobenzyl)-1^1-(3-hydroxypropyl)-1^2,1^3,1^6,1^7-tetrahydro-1^1*H*-2,4-dioxa-1(8,3)-purina-3(1,3)-benzenacycloundecaphan-6-ene-1^2,1^6-dione (34c). Compound **34c** was prepared using a similar procedure to that described above for the synthesis of **34a** by **33c**. The residue was purified by prep-HPLC (column: Welch Ultimate C18 150 mm × 25 mm × 5 μm; mobile phase: [water (TFA)-ACN]; B%: 52%–82%, 10 min) to give **34c** as a white solid (15.5 mg, 16.1% yield). ^{1}H NMR (400 MHz, DMSO-d_6): δ 7.52–7.39 (m, 4H), 7.31 (t, J = 8.2 Hz, 1H), 6.96 (t, J = 2.1 Hz, 1H), 6.89–6.74 (m, 2H), 5.52 (br s, 2H), 5.40 (s, 2H), 4.61 (br s, 2H), 4.45 (br s, 1H), 3.92 (br t, J = 7.3 Hz, 2H), 3.82 (br t, J = 7.6 Hz, 2H), 3.43 (br t, J = 6.0 Hz, 2H), 2.06 (br d, J = 12.4 Hz, 2H), 1.74–1.61 (m, 4H), 1.30 (br d, J = 6.5 Hz, 2H). LC–MS: 537.4 ([M+H]$^+$).

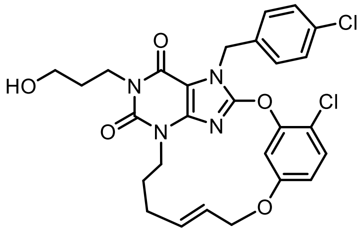

(1^8*E*,6*E*)-3^4-Chloro-1^7-(4-chlorobenzyl)-1^1-(3-hydroxypropyl)-1^2,1^3,1^6,1^7-tetrahydro-1^1*H*-2,4-dioxa-1(8,3)-purina-3(1,3)-benzenacyclodecaphan-6-ene-1^2,1^6-dione (34d). Compound **34d** was prepared using a similar procedure to that described above for the synthesis of **34a** by **33d**. The residue was purified by prep-HPLC (column: Welch Ultimate C18 150 mm × 25 mm × 5 μm; mobile phase: [water (TFA)-ACN]; B%: 69%–99%, 10 min) to give **34d** as a white solid (9.6 mg, 10.1% yield). ^{1}H NMR (400 MHz, DMSO-d_6):

δ 7.95 (d, J = 2.88 Hz, 1H), 7.42–7.48 (m, 5H), 6.85 (dd, J = 8.88, 2.75 Hz, 1H), 5.94–6.05 (m, 1H), 5.65 (dt, J = 15.60, 5.58 Hz, 1H), 5.45 (s, 2H), 4.71 (br d, J = 5.50 Hz, 2H), 4.45 (t, J = 5.19 Hz, 1H), 3.87–3.99 (m, 4H), 3.43 (br d, J = 5.50 Hz, 2H), 2.07 (br s, 2H), 1.64–1.78 (m, 4H). LC–MS: 557.0 ($[M+H]^+$).

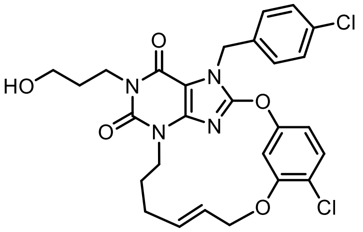

(1^8E,6E)-3^6-Chloro-1^7-(4-chlorobenzyl)-1^1-(3-hydroxypropyl)-1^2,1^3,1^6,1^7-tetrahydro-1^1H-2,4-dioxa-1(8,3)-purina-3(1,3)-benzenacyclodecaphan-6-ene-1^2,1^6-dione (34e). Compound **34e** was prepared using a similar procedure to that described above for the synthesis of **34a** by **33e**. The residue was purified by prep-HPLC (column: Welch Ultimate C18 150 mm × 25 mm × 5 μm; mobile phase: [water (TFA)-ACN]; B%: 52%–82%, 10 min) to give **34e** as a white solid (34.0 mg, 34.6% yield). ^{1}H NMR (400 MHz, DMSO-d_6): δ 7.96 (d, J = 2.75 Hz, 1H), 7.37–7.52 (m, 5H), 6.85-6.96 (m, 1H), 6.02 (br dd, J = 14.38, 7.50 Hz, 1H), 5.62–5.78 (m, 1H), 5.43 (s, 2H), 4.82 (br d, J = 5.50 Hz, 2H), 3.88–4.03 (m, 4H), 3.43 (br t, J = 6.44 Hz, 2H), 2.02-2.12 (m, 2H), 1.66–1.79 (m, 4H). LC–MS: 557.0 ($[M+H]^+$).

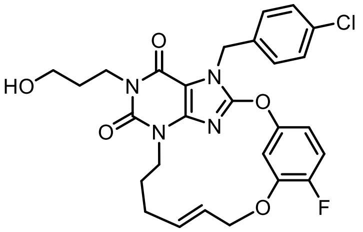

(1^8E,6E)-1^7-(4-Chlorobenzyl)-3^4-fluoro-11-(3-hydroxypropyl)-1^2,1^3,1^6,1^7-tetrahydro-1^1H-2,4-dioxa-1(8,3)-purina-3(1,3)-benzenacyclodecaphan-6-ene-1^2,1^6-dione (34f). Compound **34g** was prepared using a similar procedure to that described above for the synthesis of **34a** by **33f**. The residue was purified by prep-HPLC (column: Welch Ultimate C18 150 mm × 25 mm × 5 μm; mobile phase: [water (TFA)-ACN]; B%: 47%–77%, 10 min) to give **34f** as a white solid (43 mg, 45.1% yield). ^{1}H NMR (400 MHz, DMSO-d_6): δ 7.94 (dd, J = 7.50, 2.88 Hz, 1H), 7.44 (s, 4 H), 7.25 (dd, J=10.88, 9.01 Hz, 1 H), 6.84 (dt, J = 8.82, 3.22 Hz, 1H), 6.10–5.98 (m, 1H), 5.68 (dt, J = 15.35, 5.77 Hz, 1H), 5.42 (s, 2H),

4.78 (br d, J = 5.63 Hz, 2H), 4.00–3.84 (m, 4H), 3.43 (br t, J = 6.25 Hz, 2H), 2.07 (br s, 2H), 1.80–1.62 (m, 4H). LC–MS: 541.6 ($[M+H]^+$).

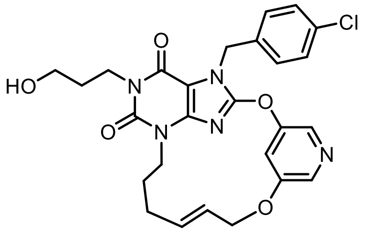

(1^8E,6E)-1^7-(4-Chlorobenzyl)-1^1-(3-hydroxypropyl)-1^2,1^3,1^6,1^7-tetrahydro-1^1H-2,4-dioxa-1(8,3)-purina-3(3,5)-pyridinacyclodecaphan-6-ene-1^2,1^6-dione (34g). Compound **34g** was prepared using a similar procedure to that described above for the synthesis of **34a** by **33g**. The residue was purified by prep-TLC (Petroleum ether/Ethyl acetate = 10/1) to give **34g** as a white solid (190 mg, 88.0% yield). ^{1}H NMR (400 MHz, $CDCl_3$): δ 8.57–8.50 (m, 1H), 8.44–8.11 (m, 2H), 7.49–7.33 (m, 4H), 6.10–5.98 (m, 1H), 5.71 (dt, J = 15.57, 5.60 Hz, 1H), 5.49-5.41 (m, 2H), 4.80 (br d, J = 5.50 Hz, 2H), 4.20 (br t, J = 5.94 Hz, 2H), 4.11–4.02 (m, 2H), 3.55 (br t, J = 5.38 Hz, 2H), 2.12–2.22 (m, 2H), 1.79–1.98 (m, 4H).

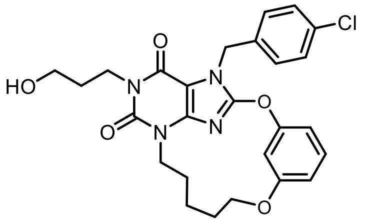

(E)-1^7-(4-Chlorobenzyl)-1^1-(3-hydroxypropyl)-1^2,1^3,1^6,1^7-tetrahydro-1^1H-2,4-dioxa-1(8,3)-purina-3(1,3)-benzenacyclononaphane-1^2,1^6-dione (5). To a solution of Lindlar (100 mg, 58.9 μmol, 1.00 eq.) in EtOAc (2.00 mL) was added **34a** (30.0 mg, 58.9 μmol, 1.00 eq.) in EtOAc (10 mL). The suspension was degassed and purged with H_2 for 3 times, the mixture was stirred under H_2 (15 psi) at rt for 1.5 h. Filtered the mixture and the filtrate was concentrated to get the crude product. The residue was purified by prep-HPLC (column: Welch Ultimate C18 150 mm × 25 mm × 5 μm; mobile phase: [water (TFA)-ACN]; B%: 52%–82%, 10 min) to give compound **5** as a white solid (3.00 mg, 9.96% yield). ^{1}H NMR (400 MHz, MeOD-d_4): δ 7.53–7.46 (m, 2H), 7.41–7.35 (m, 2H), 7.27 (t, J = 8.3 Hz, 1H), 7.08 (t, J = 2.3 Hz, 1H), 6.83–6.77 (m, 1H), 6.73 (td, J = 1.2, 8.4 Hz, 1H), 5.46 (s, 2H), 4.13–4.06 (m, 4H), 4.02 (t, J = 5.9 Hz, 2H), 3.60 (t, J = 6.4 Hz, 2H), 1.96–

1.76 (m, 6H), 1.31–1.23 (m, 2H). LC–MS: 511.2 ([M+H]$^+$). HPLC RT: 2.37 min, purity 99.9%.

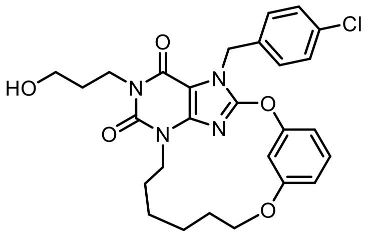

(*E*)-1^7-(4-Chlorobenzyl)-1^1-(3-hydroxypropyl)-1^2,1^3,1^6,1^7-tetrahydro-1^1*H*-2,4-dioxa-1(8,3)-purina-3(1,3)-benzenacyclodecaphane-1^2,1^6-dione (6). Compound **6** was prepared using a similar procedure to that described above for the synthesis of **5** by **34b**. The residue was purified by prep-HPLC (column: Welch Ultimate C18 150 mm × 25 mm × 5 μm; mobile phase: [water (TFA)-ACN]; B%: 58%–88%, 50 min) to give **6** as a white solid (10.9 mg, 21.6% yield). ^{1}H NMR (400 MHz, DMSO-d_6): δ 7.94 (s, 1H), 7.45 (s, 4H), 7.28 (t, J = 8.3 Hz, 1H), 6.84 (dd, J = 1.3, 8.4 Hz, 1H), 6.76 (dd, J = 1.9, 8.3 Hz, 1H), 5.44 (s, 2H), 4.47 (t, J = 5.3 Hz, 1H), 4.21–4.10 (m, 2H), 3.98–3.87 (m, 4H), 3.46–3.40 (m, 2H), 1.78 (br d, J = 2.4 Hz, 2H), 1.74–1.62 (m, 4H), 1.48–1.35 (m, 4H). LC–MS: 525.3 ([M+H]$^+$). HPLC RT: 2.46 min, purity 99.5%.

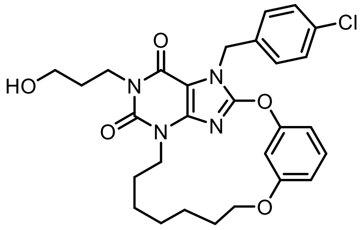

(*E*)-1^7-(4-Chlorobenzyl)-1^1-(3-hydroxypropyl)-1^2,1^3,1^6,1^7-tetrahydro-1^1*H*-2,4-dioxa-1(8,3)-purina-3(1,3)-benzenacycloundecaphane-1^2,1^6-dione (7). Compound **7** was prepared using a similar procedure to that described above for the synthesis of **5** by **34c**. The residue was purified by prep-HPLC (column: Welch Ultimate C18 150 mm × 25 mm × 5 μm; mobile phase: [water (TFA)-ACN]; B%: 58%–88%, 10 min) to give **7** as a white solid (4.62 mg, 45.6% yield,). ^{1}H NMR (400 MHz, $CDCl_3$): δ 7.48 (d, J = 8.0 Hz, 2H), 7.40–7.30 (m, 3H), 6.97 (s, 1H), 6.89 (br d, J = 7.9 Hz, 1H), 6.79 (br d, J = 8.8 Hz, 1H), 5.41 (s, 2H), 4.20 (br t, J = 5.6 Hz, 2H), 4.13 (br t, J = 7.4 Hz, 2H), 3.95–3.89 (m, 2H), 3.54 (t, J = 5.2 Hz, 2H), 1.91 (br s, 2H), 1.51–1.24 (m, 10H). LC–MS: 539.4 ([M+H]$^+$). HPLC RT: 2.55 min, purity 99.7%.

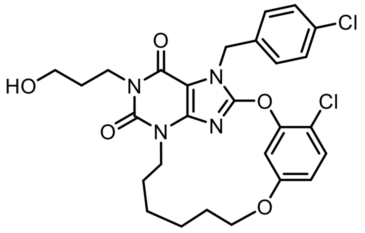

(E)-3^6-Chloro-1^7-(4-chlorobenzyl)-1^1-(3-hydroxypropyl)-1^2,1^3,1^6,1^7-tetrahydro-1^1*H*-2,4-dioxa-1(8,3)-purina-3(1,3)-benzenacyclodecaphane-1^2,1^6-dione (8). Compound **8** was prepared using a similar procedure to that described above for the synthesis of **5** by **34d**. The residue was purified by prep-HPLC (column: Welch Ultimate C18 150 mm × 25 mm × 5 μm; mobile phase: [water (TFA)-ACN]; B%: 57%–87%, 10 min) to give **8** as a white solid (20.1 mg, 45.9% yield). ^{1}H NMR (400 MHz, $CDCl_3$): δ 8.17 (d, J = 2.88 Hz, 1H), 7.50–7.66 (m, 2H), 7.31–7.37 (m, 3H), 6.77 (dd, J = 8.88, 2.88 Hz, 1H), 5.52 (s, 2H), 4.13–4.27 (m, 4H), 4.00–4.07 (m, 2H), 3.50–3.57 (m, 2H), 1.86–1.97 (m, 6H), 1.45–1.61 (m, 4H). LC–MS: 559.2 ($[M+H]^+$). HPLC RT: 2.81 min, purity 96.5%.

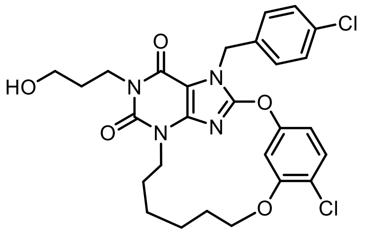

(E)-3^4-Chloro-1^7-(4-chlorobenzyl)-1^1-(3-hydroxypropyl)-1^2,1^3,1^6,1^7-tetrahydro-1^1*H*-2,4-dioxa-1(8,3)-purina-3(1,3)-benzenacyclodecaphane-1^2,1^6-dione (9). Compound **9** was prepared using a similar procedure to that described above for the synthesis of **5** by **34e**. The residue was purified by prep-HPLC (column: Welch Ultimate C18 150 mm × 25 mm × 5 μm; mobile phase: [water (TFA)-ACN]; B%: 60%–80%, 10 min) to give **9** as a white solid (33 mg, 21.8% yield). ^{1}H NMR (400 MHz, DMSO-d_6): δ 8.11 (d, J = 2.50 Hz, 1H), 7.37–7.51 (m, 5H), 6.91 (dd, J = 8.76, 2.63 Hz, 1H), 5.43 (s, 2H), 4.20–4.33 (m, 2H), 3.83–3.99 (m, 4H), 3.43 (br s, 2H), 1.80 (br s, 2H), 1.63–1.74 (m, 4H), 1.38–1.48 (m, 4H). LC–MS: 559.2 ($[M+H]^+$). HPLC RT: 2.69 min, purity 99.3%.

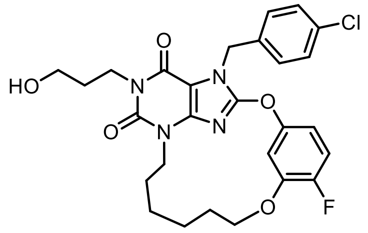

(*E*)-1^7-(4-Chlorobenzyl)-3^4-fluoro-11-(3-hydroxypropyl)-1^2,1^3,1^6,1^7-tetrahydro-1^1*H*-2,4-dioxa-1(8,3)-purina-3(1,3)-benzenacyclodecaphane-1^2,1^6-dione (10). Compound **10** was prepared using a similar procedure to that described above for the synthesis of **5** by **34f**. The residue was purified by prep-HPLC (column: Welch Ultimate C18 150 mm × 25 mm × 5 μm; mobile phase: [water (TFA)-ACN]; B%: 60%–80%, 10 min) to give **10** as a white solid (16.0 mg, 10.6% yield). ^{1}H NMR (400 MHz, DMSO-d_6): δ 8.08 (dd, *J* = 7.50, 2.75 Hz, 1H), 7.45 (s, 4H), 7.26 (dd, *J* = 11.01, 9.01 Hz, 1H), 6.87 (dt, *J* = 8.72, 3.14 Hz, 1H), 5.43 (s, 2H), 4.30–4.21 (m, 2H), 3.96–3.84 (m, 4H), 3.43 (br t, *J* = 6.44 Hz, 3H), 1.80 (br s, 2H), 1.73–1.62 (m, 4H), 1.42 (br s, 4H). LC–MS: 543.2 ([M+H]$^+$). HPLC RT: 2.56 min, purity 99.3%.

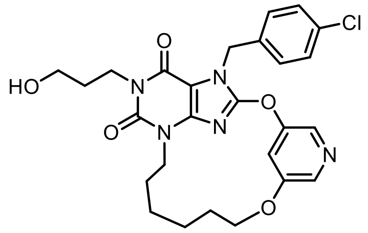

(*E*)-1^7-(4-Chlorobenzyl)-1^1-(3-hydroxypropyl)-1^2,1^3,1^6,1^7-tetrahydro-1^1*H*-2,4-dioxa-1(8,3)-purina-3(3,5)-pyridinacyclodecaphane-1^2,1^6-dione (11). Compound **11** was prepared using a similar procedure to that described above for the synthesis of **5** by **34g**. The residue was purified by prep-HPLC (column: Welch Ultimate C18 150 mm × 25 mm × 5 μm; mobile phase: [water (TFA)-ACN]; B%: 35%–65%, 10 min) to give **11** as a white solid (5 mg, 4.98% yield). ^{1}H NMR (400 MHz, $CDCl_3$): δ 8.32 (s, 1H), 8.25–7.96 (m, 2H), 7.38 (br d, *J* = 8.38 Hz, 2H), 7.27 (br d, *J* = 8.50 Hz, 2H), 5.38 (s, 2H), 4.21–4.07 (m, 4H), 4.01–3.88 (m, 2H), 3.65 (q, *J* = 7.00 Hz, 1H), 3.46 (br t, *J* = 5.50 Hz, 2H), 1.89–1.80 (m, 4H), 1.52–1.39 (m, 4H), 1.17 (t, *J* = 7.00 Hz, 2H). LC–MS: 526.4 ([M+H]$^+$). HPLC RT: 2.32 min, purity 95.2%.

3.3. General procedures for the synthesis of compounds ***12–16***

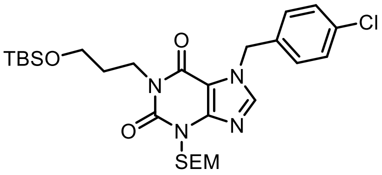

1-(3-((*tert*-Butyldimethylsilyl)oxy)propyl)-7-(4-chlorobenzyl)-3-((2-(trimethylsilyl)ethoxy)methyl)-3,7-dihydro-1*H*-purine-2,6-dione (35). To a solution of **28** (20.0 g, 49.1 mmol, 1.00 eq.) and (3-bromopropoxy)(*tert*-butyl)dimethylsilane (14.9 g, 58.9 mmol, 1.20 eq.) in DMF (100 mL) was added K_2CO_3 (10.1 g, 73.7 mmol, 1.50 eq.). The mixture was stirred at 80 ℃ for 16 h. The reaction mixture was partitioned between H_2O 20.0 mL and EtOAc 30.0 mL. The organic phase was separated, filtered and concentrated under reduced pressure to give compound **35** as white solid (25.0 g, 88.1% yield). LC–MS: 579.2 ($[M+H]^+$).

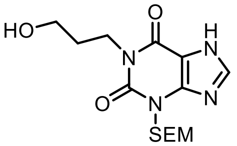

1-(3-Hydroxypropyl)-3-((2-(trimethylsilyl)ethoxy)methyl)-3,7-dihydro-1*H*-purine-2,6-dione (36). To a solution of **35** (25.0 g, 43.1 mmol, 1.00 eq.) in MeOH (250 mL) was added Pd/C (10.0 g, 9.40 mmol, 10% purity). The mixture was stirred under H_2 (50 psi) at 50 ℃ for 4 h. Filtered and concentrated under reduced pressure to give a residue. The residue was purified by column chromatography (Petroleum ether/Ethyl acetate = 10/1) to give **36** as a white solid (14.0 g, 95.2% yield). LC–MS: 341.1 ($[M+H]^+$).

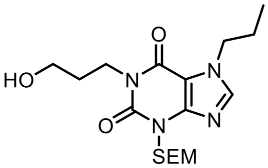

1-(3-Hydroxypropyl)-7-propyl-3-((2-(trimethylsilyl)ethoxy)methyl)-3,7-dihydro-1*H*-purine-2,6-dione (37a). To a solution of **36** (100 mg, 294 μmol, 1.00 eq.) in DMF (1 mL) was added DIEA (75.9 mg, 587 μmol, 102 μL, 2.00 eq.) and 1-bromopropane (72.3 mg, 587 μmol, 53.4 μL, 2.00 eq.). The mixture was stirred at 70 ℃ for 2 h. The reaction mixture was partitioned between H_2O 10 mL and EtOAc 20.0 mL. The organic phase was separated, filtered and concentrated under reduced pressure to give a residue. The residue was purified

by column chromatography (SiO_2, Ethyl acetate) to give **37a** as colorless oil (80.0 g, 71.2% yield). LC–MS: 383.1 ($[M+H]^+$).

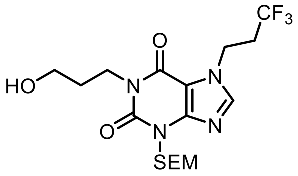

1-(3-Hydroxypropyl)-7-(3,3,3-trifluoropropyl)-3-((2-(trimethylsilyl)ethoxy)methyl)-3,7-dihydro-1H-purine-2,6-dione (37b). To a solution of **36** (0.1 g, 294 μmol, 1.00 eq.) in DMF (1 mL) was added Cs_2CO_3 (143.55 mg, 440.59 μmol, 1.50 eq.) and 1, 1, 1-trifluoro-3-iodo-propane (131.57 mg, 587.46 μmol, 68.85 μL, 2.00 eq.). The mixture was stirred at 100 ℃ for 16 h. The reaction mixture was partitioned between H_2O 10 mL and EtOAc 20 mL. The organic phase was separated, filtered and concentrated under reduced pressure to give a residue. The residue was purified by column chromatography (SiO_2, Ethyl acetate) to give **37b** as colorless oil (30 mg, 23.4% yield). LC–MS: 437.2 ($[M+H]^+$).

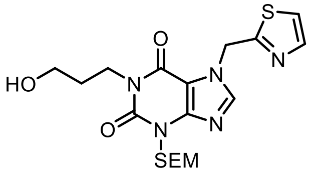

1-(3-Hydroxypropyl)-7-(thiazol-2-ylmethyl)-3-((2-(trimethylsilyl)ethoxy)methyl)-3,7-dihydro-1*H*-purine-2,6-dione (37c). To a solution of **36** (1.5 g, 4.41 mmol, 1.00 eq.) and 2-(chloromethyl)thiazole (706.36 mg, 5.29 mmol, 1.20 eq.) in DMF (15 mL) was added Cs_2CO_3 (2.15 g, 6.61 mmol, 1.50 eq.). The mixture was stirred at 80 ℃ for 16 h. The reaction mixture was partitioned between H_2O 20 mL and EtOAc 20 mL. The organic phase was separated, filtered and concentrated under reduced pressure to give compound **37c** as brown oil (1.8 g, 93.3% yield). LC–MS: 438.1 ($[M+H]^+$).

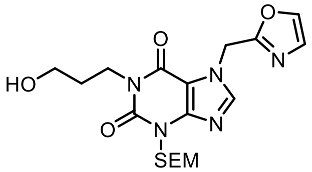

1-(3-Hydroxypropyl)-7-(oxazol-2-ylmethyl)-3-((2-(trimethylsilyl)ethoxy)methyl)-3,7-dihydro-1*H*-purine-2,6-dione (37d). A mixture of **36** (1.5 g, 4.41 mmol, 1.00 eq.), 2-(chloromethyl)oxazole (**2e**, 621.41 mg, 5.29 mmol, 1.20 eq.), Cs_2CO_3 (2.87 g, 8.81 mmol, 2.00 eq.) in DMF (15 mL) was degassed, and then the mixture was stirred at rt for 16 h

under N_2 atmosphere. The reaction mixture was partitioned between H_2O 20 mL and EtOAc 20 mL. The organic phase was separated, filtered and concentrated under reduced pressure to give a residue. The residue was purified by column chromatography (SiO_2, DCM: MeOH = 10:1) to give **37d** as a white oil (1.3 g, 70.0% yield). LC–MS: 422.1 ($[M+H]^+$).

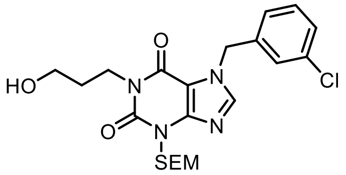

7-(3-Chlorobenzyl)-1-(3-hydroxypropyl)-3-((2-(trimethylsilyl)ethoxy)methyl)-3,7-dihydro-1*H*-purine -2,6-dione (37e). Compound **37e** was prepared using a similar procedure to that described above for the synthesis of **37d** by 1-chloro-3-(chloromethyl)benzene. The combined organic layers were washed with aq. NaCl 3.00 mL concentrated under reduced pressure to give **37e** as a yellow solid (1.5g, 73.2% yield). LC–MS: 465.1 ($[M+H]^+$).

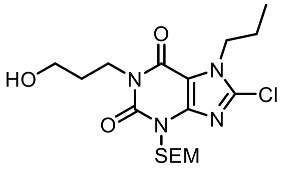

8-Chloro-1-(3-hydroxypropyl)-7-propyl-3-((2-(trimethylsilyl)ethoxy)methyl)-3,7-dihydro-1*H*-purine-2,6-dione (38a). To a solution of **37a** (1.00 g, 2.61 mmol, 1.00 eq.) in THF (10 mL) was added NCS (419 mg, 3.14mmol, 1.20 eq.). The mixture was stirred at rt for 2 h. The reaction mixture was partitioned between H_2O 20.0 mL and EtOAc 30.0 mL. The organic phase was separated, filtered and concentrated under reduced pressure to give compound **38a** as yellow oil (1.0 g, 92.2% yield). LC–MS: 439.2 ($[M+Na]^+$).

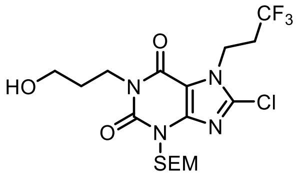

8-Chloro-1-(3-hydroxypropyl)-7-(3,3,3-trifluoropropyl)-3-((2-(trimethylsilyl)ethoxy)methyl)-3,7-dihydro-1*H*-purine-2,6-dione (38b). Compound **38b** was prepared using a similar procedure to that described above for the synthesis of

38a by **37b**. Filtered and concentrated under reduced pressure to give compound **38b** as yellow oil (130 mg, 85.3%). LC–MS: 493.0 ([M+Na]$^+$).

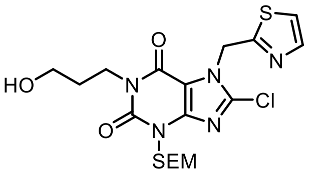

8-Chloro-1-(3-hydroxypropyl)-7-(thiazol-2-ylmethyl)-3-((2-(trimethylsilyl)ethoxy)methyl)-3,7-dihydro-1*H*-purine-2,6-dione (38c). Compound **38c** was prepared using a similar procedure to that described above for the synthesis of **38a** by **37c**. Filtered and concentrated under reduced pressure to give compound **38c** as brown oil (1.8 g, 92.8%). LC–MS: 472.0 ([M+H]$^+$).

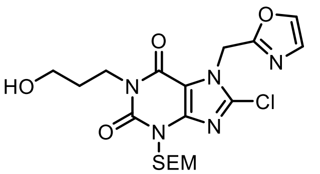

8-Chloro-1-(3-hydroxypropyl)-7-(oxazol-2-ylmethyl)-3-((2-(trimethylsilyl)ethoxy)methyl)-3,7-dihydro-1*H*-purine-2,6-dione (38d). Compound **38d** was prepared using a similar procedure to that described above for the synthesis of **38a** by **37d**. Filtered and concentrated under reduced pressure to give compound **38d** as a white solid (1.05 g, 76.7%). LC–MS: 455.9 ([M+H]$^+$).

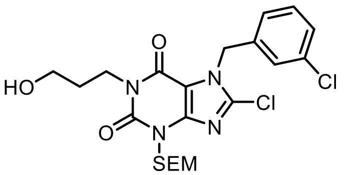

8-Chloro-7-(3-chlorobenzyl)-1-(3-hydroxypropyl)-3-((2-(trimethylsilyl)ethoxy)methyl)-3,7-dihydro-1*H*-purine-2,6-dione (38e). Compound **38e** was prepared using a similar procedure to that described above for the synthesis of **38a** by **37e**. The combined organic layers were washed with aq. NaCl 3.0 mL, concentrated under reduced pressure to give **38e** as a yellow solid (2.0 g, 93.5% yield). LC–MS: 499.1 ([M+H]$^+$).

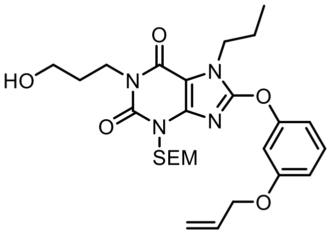

8-(3-(Allyloxy)phenoxy)-1-(3-hydroxypropyl)-7-propyl-3-((2-(trimethylsilyl)ethoxy)methyl)-3,7-dihydro-1*H*-purine-2,6-dione (39a). To a solution of **38a** (1.00 g, 2.40 mmol, 1.00eq.) in DMF (10.0 mL) was added K_2CO_3 (497 mg, 3.60 mmol, 1.50 eq.) and 3-(allyloxy)phenol (540 mg, 3.60 mmol, 1.50 eq.). The mixture was stirred at 80 ℃ for 16 h. The reaction mixture was partitioned between H_2O 20 mL and EtOAc 30.0 mL. The organic phase was separated, filtered and concentrated under reduced pressure to give a residue. The residue was purified by column chromatography (SiO_2, Petroleum ether/Ethyl acetate=1/1) to give compound **39a** as yellow oil (540 mg, 42.4% yield). LC–MS: 531.4 ($[M+H]^+$).

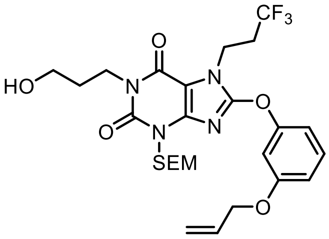

8-(3-(Allyloxy)phenoxy)-1-(3-hydroxypropyl)-7-(3,3,3-trifluoropropyl)-3-((2-(trimethylsilyl) ethoxy)methyl)-3,7-dihydro-1*H*-purine-2,6-dione (39b). Compound **39b** was prepared using a similar procedure to that described above for the synthesis of **39a** by **38b**. The reaction mixture was partitioned between H_2O 10 mL and EtoAc 20 mL. The organic phase was separated, filtered and concentrated under reduced pressure to give compound **39b** as brown oil (195 mg, 78.9% yield). LC–MS: 585.4 ($[M+H]^+$).

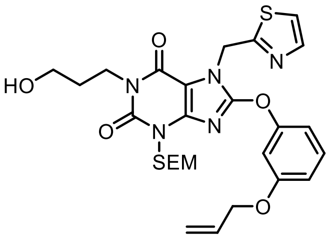

8-(3-(Allyloxy)phenoxy)-1-(3-hydroxypropyl)-7-(thiazol-2-ylmethyl)-3-((2-(trimethylsilyl) ethoxy)methyl)-3,7-dihydro-1*H*-purine-2,6-dione (39c). Compound

39c was prepared using a similar procedure to that described above for the synthesis of **39a** by **38c**. The reaction mixture was partitioned between H_2O 25 mL and EtOAc 30 mL. The organic phase was separated, filtered and concentrated under reduced pressure to give compound **39c** as brown oil (2.0 g, 90.1% yield). LC–MS: 586.2 ([M+H]$^+$).

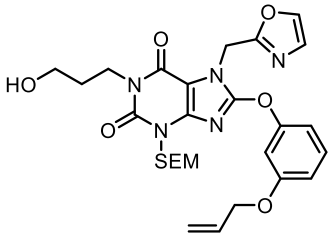

8-(3-(Allyloxy)phenoxy)-1-(3-hydroxypropyl)-7-(oxazol-2-ylmethyl)-3-((2-(trimethylsilyl) ethoxy)methyl)-3,7-dihydro-1*H*-purine-2,6-dione (39d). Compound **39d** was prepared using a similar procedure to that described above for the synthesis of **39a** by **38d**. The reaction mixture was partitioned between H_2O 10 mL and EtoAc 20 mL. The organic phase was separated, filtered and concentrated under reduced pressure to give compound **39d** as a white solid (1.3 g, 99.1% yield). LC–MS: 570.5 ([M+H]$^+$).

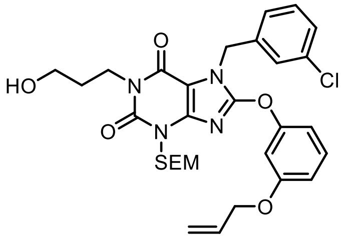

8-(3-(Allyloxy)phenoxy)-7-(3-chlorobenzyl)-1-(3-hydroxypropyl)-3-((2-(trimethylsilyl)ethoxy) methyl)-3,7-dihydro-1H-purine-2,6-dione (39e). Compound **39e** was prepared using a similar procedure to that described above for the synthesis of **39a** by **38e**. The reaction mixture was quenched by addition H_2O 50.0 mL, and then extracted with EtOAc 50.0 mL (25.0 mL × 2). The combined organic layers were washed with aq. NaCl 5.0 mL, concentrated under reduced pressure to give compound **39e** as a yellow solid (6.0 g, 98.0% yield). LC–MS: 613.2 ([M+H]$^+$).

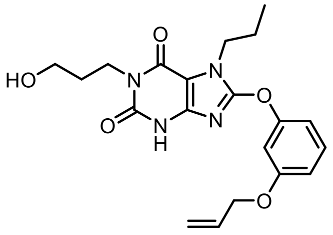

8-(3-(Allyloxy)phenoxy)-1-(3-hydroxypropyl)-7-propyl-3,7-dihydro-1*H*-purine-2,6-dione (40a). To a solution of **39a** (540 mg, 1.02mmol, 1.00 eq.) in EtOH (5 mL) was added HCl (12 mol/L, 0.5 mL). The mixture was stirred at 80 ℃ for 16 h. The reaction mixture was partitioned between H_2O 10.0 mL and EtOAc 20.0 mL. The organic phase was separated, filtered and concentrated under reduced pressure to give compound **40a** as brown oil (300 mg, 74.9%). LC–MS: 400.9 ($[M+H]^+$).

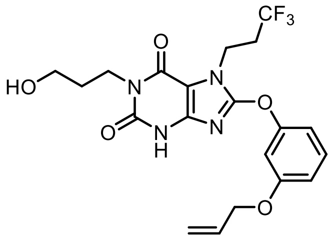

8-(3-(Allyloxy)phenoxy)-1-(3-hydroxypropyl)-7-(3,3,3-trifluoropropyl)-3,7-dihydro-1H-purine-2,6-dione (40b). Compound **40b** was prepared using a similar procedure to that described above for the synthesis of **40a** by **39b**. The residue was purified by column chromatography (SiO_2, Petroleum ether/Ethyl acetate=1/1) to give compound **40b** as a white solid (0.69 g, 23.4% yield). LC–MS: 455.2 ($[M+H]^+$).

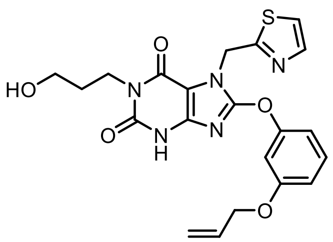

8-(3-(Allyloxy)phenoxy)-1-(3-hydroxypropyl)-7-(thiazol-2-ylmethyl)-3,7-dihydro-1*H*-purine-2,6-dione (40c). Compound **40c** was prepared using a similar procedure to that described above for the synthesis of **40a** by **39c**. The residue was purified by column chromatography (SiO_2, Petroleum ether/Ethyl acetate=1/1) to give compound **40c** as a brown solid (1.25 g, 59.5% yield). LC–MS: 456.0 ($[M+H]^+$).

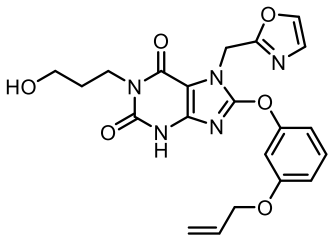

8-(3-(Allyloxy)phenoxy)-1-(3-hydroxypropyl)-7-(oxazol-2-ylmethyl)-3,7-dihydro-1*H*-purine-2,6-dione (40d). Compound **40d** was prepared using a similar procedure to that described above for the synthesis of **40a** by **39d**. The residue was purified by column chromatography (SiO_2, Petroleum ether/Ethyl acetate = 1/1) to give compound **40d** as a white solid (110 mg, 11.0% yield). LC–MS: 440.0 ($[M+H]^+$).

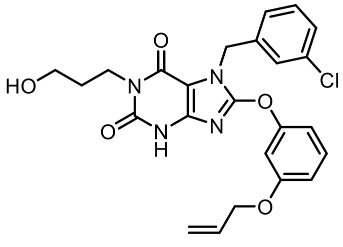

8-(3-(Allyloxy)phenoxy)-7-(3-chlorobenzyl)-1-(3-hydroxypropyl)-3,7-dihydro-1*H*-purine-2,6-dione (40e). Compound **40e** was prepared using a similar procedure to that described above for the synthesis of **40a** by **39e**. The residue was purified by column chromatography (Petroleum ether/Ethyl acetate= 50/1 to 2/1) to give compound **40e** as a yellow solid (2.00 g, 42.3% yield). LC–MS: 483.1 ($[M+H]^+$).

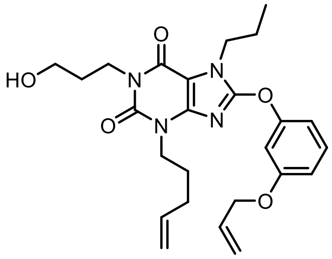

8-(3-(Allyloxy)phenoxy)-1-(3-hydroxypropyl)-3-(pent-4-en-1-yl)-7-propyl-3,7-dihydro-1*H*-purine-2,6-dione (41a). To a solution of compound **40a** (300 mg, 749 μmol, 1.00 eq.) in DMF (2.00 mL) was added Cs_2CO_3 (488 mg, 1.50 mmol, 2.00 eq.) and compound 5-bromopent-1-ene (134 mg, 899 μmol, 106 μL, 1.20 eq.). The mixture was stirred at 80 ℃ for 1 h. The reaction mixture was partitioned between H_2O 10.0 mL and EtOAc 20.0mL. The organic phase was separated, filtered and concentrated under reduced pressure to give compound **41a** as brown oil (300 mg, 85.5% yield). LC–MS: 469.4 ($[M+H]^+$).

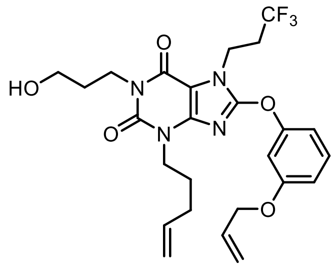

8-(3-(Allyloxy)phenoxy)-1-(3-hydroxypropyl)-3-(pent-4-en-1-yl)-7-(3,3,3-trifluoropropyl)-3,7-dihydro-1*H*-purine-2,6-dione (41b). Compound **41b** was prepared using a similar procedure to that described above for the synthesis of **41a** by **40b**. The reaction mixture was partitioned between H_2O 10 mL and EtOAc 20 mL. The organic phase was separated, filtered and concentrated under reduced pressure to give a residue. The residue was purified by column chromatography (Petroleum ether/Ethyl acetate = 1/1) to give compound **41b** as brown oil (78.81 mg, 68.5% yield). LC–MS: 535.3 ($[M+Na]^+$).

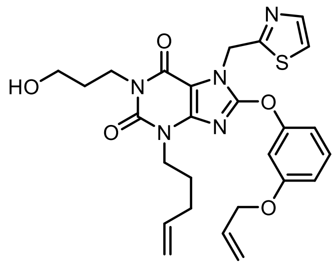

8-(3-(Allyloxy)phenoxy)-1-(3-hydroxypropyl)-3-(pent-4-en-1-yl)-7-(thiazol-2-ylmethyl)-3,7-dihydro-1*H*-purine-2,6-dione (41c). Compound **41c** was prepared using a similar procedure to that described above for the synthesis of **41a** by **40c**. The reaction mixture was partitioned between H_2O 10 mL and EtOAc 20 mL. The organic phase was separated, filtered and concentrated under reduced pressure to give a residue. The residue was purified by column chromatography (Petroleum ether/Ethyl acetate = 1/1) to give compound **41c** as brown oil (140 mg, 30.5% yield). LC–MS: 524.2 ($[M+H]^+$).

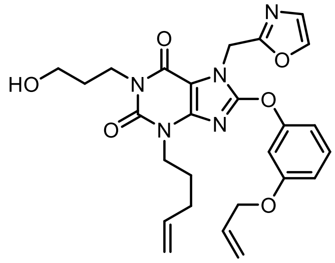

8-(3-(Allyloxy)phenoxy)-1-(3-hydroxypropyl)-7-(oxazol-2-ylmethyl)-3-(pent-4-en-1-yl)-3,7-dihydro-1*H*-purine-2,6-dione (41d). Compound **41d** was prepared using a similar

procedure to that described above for the synthesis of **41a** by **40d**. The reaction mixture was partitioned between H_2O 10 mL and EtOAc 20 mL. The organic phase was separated, filtered and concentrated under reduced pressure to give a residue. The residue was purified by column chromatography (Petroleum ether/Ethyl acetate=1/1) to give compound **41d** as brown oil (140 mg, 30.5% yield). LC–MS: 508.2 ($[M+H]^+$).

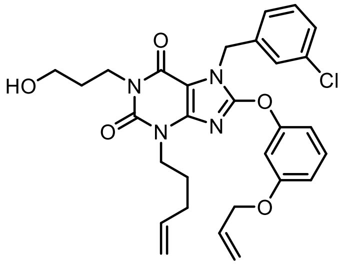

8-(3-(Allyloxy)phenoxy)-7-(3-chlorobenzyl)-1-(3-hydroxypropyl)-3-(pent-4-en-1-yl)-3,7-dihydro-1*H*-purine-2,6-dione (41e). Compound **41e** was prepared using a similar procedure to that described above for the synthesis of **41a** by **40e**. The reaction mixture was quenched by addition H_2O 20.0 mL, and then extracted with EtOAc 20.0 mL (10.0 mL × 2). The crude product was purified by silica gel column chromatography (Petroleum ether/Ethyl acetate = 1/1) to give **41e** (2.2 g, 96.9% yield) as a yellow solid. LC–MS: 551.2 ($[M+H]^+$).

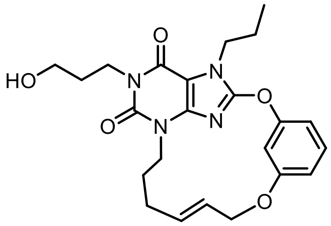

(1^8*E*,6*E*)-1^1-(3-Hydroxypropyl)-1^7-propyl-1^2,1^3,1^6,1^7-tetrahydro-1^1*H*-2,4-dioxa-1(8,3)-purina-3(1,3)-benzenacyclodecaphan-6-ene-1^2,1^6-dione (42a). To a solution of compound **41a** (100 mg, 213 μmol, 1.00 eq.) in DCM (10.0 mL) was added HG-II (40.1 mg, 64.0 μmol, 0.30 eq.). The mixture was stirred at rt for 16 h. Filtered and concentrated under reduced pressure to give a residue. The residue was purified by column chromatography (SiO_2, Petroleum ether/Ethyl acetate=2/1). Furthermore, the residue was purified by prep-HPLC (column: Welch Xtimate C18 150 mm × 25 mm × 5 μm; mobile phase: [water (TFA)-ACN]; gradient: 42%–72% B over 10 min) to give compound **42a** as off-white solid (13.7 mg, 14.5% yield). ^1H NMR (400 MHz, DMSO-d_6) δ 7.80 (t, J = 2.13 Hz, 1H), 7.28 (t, J = 8.19 Hz, 1H), 6.87 (dd, J = 8.07, 1.44 Hz, 1H), 6.80 (dd, J = 8.25,

1.63 Hz, 1H), 6.05–5.93 (m, 1H), 5.66 (dt, *J* = 15.57, 5.66 Hz, 1H), 4.69 (br d, *J* = 5.25 Hz, 2H), 4.15 (br t, *J* = 6.82 Hz, 2H), 3.98–3.86 (m, 4H), 3.44 (br s, 2H), 2.06 (br d, *J* = 6.50 Hz, 2H), 1.84–1.63 (m, 6H), 0.87 (t, *J* = 7.38 Hz, 3H). LC–MS: 441.3 ($[M+H]^+$).

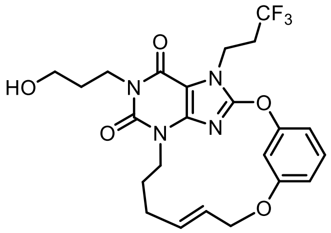

(1^8E,6E)-1^1-(3-Hydroxypropyl)-1^7-(3,3,3-trifluoropropyl)-1^2,1^3,1^6,1^7-tetrahydro-1^1H-2,4-dioxa-1(8,3)-purina-3(1,3)-benzenacyclodecaphan-6-ene-1^2,1^6-dione (42b). Compound **42b** was prepared using a similar procedure to that described above for the synthesis of **42a** by **41b**. The residue was purified by prep-HPLC (column: Phenomenex luna C18 150 mm ×25mm × 10 μm; mobile phase: [water (FA)-ACN]; gradient: 51%–81% B over 10 min) to give compound **42b** as a white solid (10.64 mg, 14.3% yield). ^{1}H NMR (400 MHz, DMSO-d_6): δ 7.78 (t, *J* = 2.19 Hz, 1H), 7.30 (t, *J* = 8.25 Hz, 1H), 6.83 (ddd, *J* = 15.20, 8.13, 1.94 Hz, 2H), 6.03-5.91 (m, 1H), 5.67 (dt, *J* = 15.60, 5.64 Hz, 1H), 4.69 (br d, *J* = 5.38 Hz, 2H), 4.44 (br t, *J* = 6.44 Hz, 3H), 4.02–3.86 (m, 4H), 3.43 (br t, *J* = 6.32 Hz, 4H), 2.99–2.84 (m, 2H), 2.06 (br s, 2H), 1.78–1.63 (m, 4H). LC–MS: 495.4 ($[M+H]^+$).

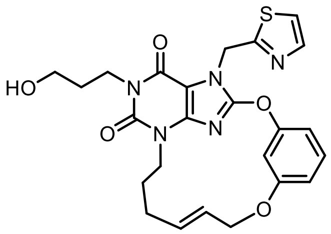

(1^8E,6E)-1^1-(3-Hydroxypropyl)-1^7-(thiazol-2-ylmethyl)-1^2,1^3,1^6,1^7-tetrahydro-1^1H-2,4-dioxa-1(8,3)-purina-3(1,3)-benzenacyclodecaphan-6-ene-1^2,1^6-dione (42c). Compound **42c** was prepared using a similar procedure to that described above for the synthesis of **42a** by **41c**. Filtered and concentrated under reduced pressure to give a residue. The residue was purified by column chromatography (SiO_2, Petroleum ether/Ethyl acetate = 1/1) to give compound **42c** as a yellow solid (54 mg, 63.4% yield). ^{1}H NMR (400 MHz, DMSO-d_6) δ 7.97–7.81 (m, 3H), 7.36 (t, *J* = 8.19 Hz, 1H), 6.94–6.82 (m, 2H), 6.23–6.06 (m, 1H), 5.88 (s, 2H), 5.77 (dt, *J* = 15.45, 5.78 Hz, 1H), 4.81 (br d, *J* = 5.38 Hz, 2H), 4.10–3.96 (m, 4H), 3.52 (br s, 2), 2.17 (br s, 2H), 1.93–1.72 (m, 4H). LC–MS: 496.0 ($[M+H]^+$).

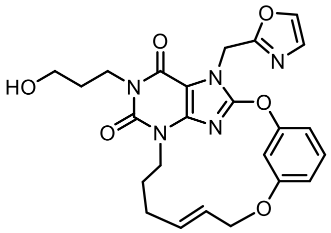

(1^8E,6*E*)-1^1-(3-Hydroxypropyl)-1^7-(oxazol-2-ylmethyl)-1^2,1^3,1^6,1^7-tetrahydro-1^1H-2,4-dioxa-1(8,3)-purina-3(1,3)-benzenacyclodecaphan-6-ene-1^2,1^6-dione (42d). Compound **42d** was prepared using a similar procedure to that described above for the synthesis of **42a** by **41d**. The residue was purified by prep-HPLC (column: Phenomenex luna C18 150 mm ×25mm × 10 μm; mobile phase: [water (FA)-ACN]; gradient: 51%–81% B over 10 min) to give compound **42d** as a white solid (4.5 mg, 23.8% yield). ^{1}H NMR (400 MHz, DMSO-d_6) δ 8.18–8.09 (m, 1H), 7.84 (s, 1H), 7.32–7.19 (m, 2H), 6.86–6.68 (m, 2H), 6.13–6.00 (m, 1H), 5.72-5.60 (m, 3H), 4.73 (br d, J = 5.38 Hz, 2H), 4.45 (t, J = 5.19 Hz, 1H), 3.98 (br t, J = 7.50 Hz, 2H), 3.88 (br t, J = 7.32 Hz, 2H), 3.41 (q, J = 6.17 Hz, 2H), 2.08 (br s, 2H), 1.62–1.79 (m, 4H). LC–MS: 4480.2 ([M+H]$^+$).

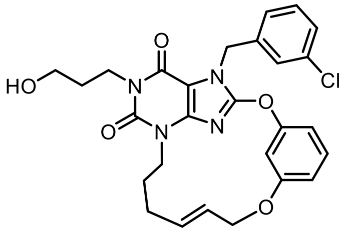

(1^8E,6*E*)-1^7-(3-Chlorobenzyl)-1^1-(3-hydroxypropyl)-1^2,1^3,1^6,1^7-tetrahydro-1^1H-2,4-dioxa-1(8,3)-purina-3(1,3)-benzenacyclodecaphan-6-ene-1^2,1^6-dione (42e). Compound **42e** was prepared using a similar procedure to that described above for the synthesis of **42a** by **41e**. The residue was purified by prep-HPLC (column: Welch Xtimate C18 150 mm × 25 mm ×5 μm; mobile phase: [water(TFA)-ACN]; gradient: 58%–78% B over 10 min) to give **42e** as an off-white solid (200 mg, 9.2% yield). ^{1}H NMR (400 MHz, DMSO-d_6): δ 7.85–7.82 (m, 1H), 7.52–7.49 (m, 1H), 7.42–7.33 (m, 3H), 7.30–7.24 (m, 1H), 6.84–6.76 (m, 2H), 6.05–5.95 (m, 1H), 5.72–5.60 (m, 1H), 5.46–5.41 (m, 2H), 4.73–4.66 (m, 2H), 3.98–3.89 (m, 4H), 3.45–3.41 (m, 2H), 2.10–2.02 (m, 2H), 1.76–1.65 (m, 4H). LC–MS: 523.3 ([M+H]$^+$).

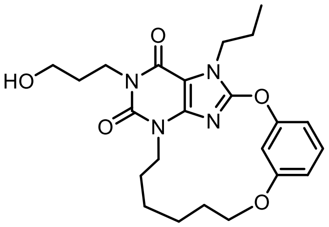

(*E*)-1¹-(3-Hydroxypropyl)-1⁷-propyl-1²,1³,1⁶,1⁷-tetrahydro-1¹*H*-2,4-dioxa-1(8,3)-purina-3(1,3)-benzenacyclodecaphane-1²,1⁶-dione (12). A mixture of **42a** (0.078 g, 177.08 μmol, 1.00 eq.) and LINDLAR (17.7 μmol, 0.10 eq.) in EtOAc (16 mL) was degassed and purged with H_2 for 3 times, and then the mixture was stirred at rt for 0.5 h under H_2 (15 psi) atmosphere. Filtered the mixture and the filtrate was concentrated to get the crude product. The residue was purified by column chromatography (SiO_2, Petroleum ether/Ethyl acetate=1/1). Furthermore, the residue was purified by prep-HPLC (column: Welch Xtimate C18 150 mm × 25 mm × 5 μm; mobile phase: [water (TFA)-ACN]; gradient: 45%–75% B over 10 min) to give compound **12** as yellow gum (40.3 mg, 50.7 % yield). ^{1}H NMR (400 MHz, DMSO-d_6): δ 7.87 (t, J = 2.25 Hz, 1H), 7.28 (t, J = 8.19 Hz, 1H), 6.87 (dd, J = 8.00, 1.50 Hz, 1H), 6.75 (dd, J = 8.25, 1.63 Hz, 1H), 4.21–4.10 (m, 4H), 3.94–3.85 (m, 4H), 3.44 (br s, 2H), 3.41 (br s, 2H), 1.85-1.63 (m, 8H), 1.40 (br s, 4H), 0.87 (t, J = 7.44 Hz, 3H). LC–MS: 443.1 ([M+H]$^+$). HPLC RT: 2.63 min, purity 98.6%.

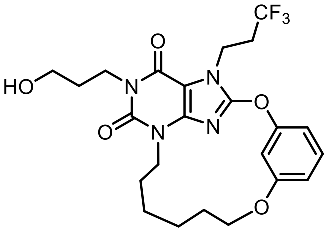

(*E*)-1¹-(3-Hydroxypropyl)-1⁷-(3,3,3-trifluoropropyl)-1²,1³,1⁶,1⁷-tetrahydro-1¹*H*-2,4-dioxa-1(8,3)-purina-3(1,3)-benzenacyclodecaphane-1²,1⁶-dione (13). Compound **13** was prepared using a similar procedure to that described above for the synthesis of **12** by **42b**. The residue was purified by prep-HPLC (column: Welch Xtimate C18 150 mm × 25 mm × 5 μm; mobile phase: [water (TFA)-ACN]; gradient: 44%–74% B over 10 min) to give compound **13** as a pink solid (44.87 mg, 44.3% yield). ^{1}H NMR (400 MHz, DMSO-d_6): δ 7.90 (t, J = 2.13 Hz, 1H), 7.32 (t, J = 8.19 Hz, 1H), 6.87 (dd, J = 8.07, 1.56 Hz, 1H), 6.78 (dd, J = 8.25, 1.63 Hz, 1H), 4.47 (br t, J = 6.57 Hz, 3H), 4.22–4.13 (m, 2H), 3.99–

3.88 (m, 4H), 3.44 (br s, 2H), 2.99-2.88 (m, 2H), 1.79 (br s, 2H), 1.75–1.65 (m, 4H), 1.43 (br s, 4H). LC–MS: 497.3 ($[M+H]^+$). HPLC RT: 2.28 min, purity 99.1%.

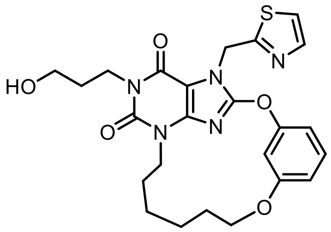

(*E*)-1[1]-(3-Hydroxypropyl)-1[7]-(thiazol-2-ylmethyl)-1[2],1[3],1[6],1[7]-tetrahydro-1[1]*H*-2,4-dioxa-1(8,3)-purina-3(1,3)-benzenacyclodecaphane-1[2],1[6]-dione (14). Compound **14** was prepared using a similar procedure to that described above for the synthesis of **12** by **42c**. The residue was purified by prep-HPLC (column: Welch Xtimate C18 150 mm × 25 mm × 5 µm; mobile phase: [water (TFA)-ACN]; gradient: 40%–70% B over 10 min) to give compound **14** as brown gum (10.89 mg, 54.2% yield). ^1H NMR (400 MHz, DMSO-d_6) δ 8.06–8.00 (m, 1H), 7.90–7.87 (m, 1H), 7.86–7.83 (m, 1H), 7.37 (t, J = 8.19 Hz, 1H), 6.90–6.83 (m, 2H), 5.90 (s, 2H), 4.56 (t, J = 5.13 Hz, 1H), 4.34–4.22 (m, 2H), 4.10–3.99 (m, 4H), 3.59–3.50 (m, 2H), 1.89 (br s, 2H), 1.84–1.74 (m, 4H), 1.53 (br s, 4H). LC–MS: 498.2 ($[M+H]^+$). HPLC RT: 2.14 min, purity 99.9%.

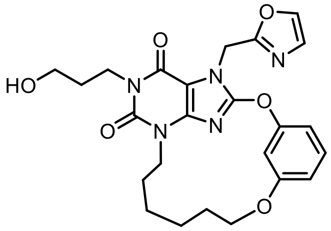

(*E*)-1[1]-(3-Hydroxypropyl)-1[7]-(oxazol-2-ylmethyl)-1[2],1[3],1[6],1[7]-tetrahydro-1[1]*H*-2,4-dioxa-1(8,3)-purina-3(1,3)-benzenacyclodecaphane-1[2],1[6]-dione (15). Compound **15** was prepared using a similar procedure to that described above for the synthesis of **12** by **42d**. The residue was purified by prep-HPLC (column: Welch Xtimate C18 150 mm × 25 mm × 5 µm; mobile phase: [water (TFA)-ACN]; gradient: 44%–74% B over 10 min) to give compound **15** as a white solid (7.9 mg, 15.6% yield). ^1H NMR (400 MHz, DMSO-d_6) δ 8.18–8.13 (m, 1H), 7.96–7.92 (m, 1H), 7.28 (t, J = 8.25 Hz, 1H), 7.21 (s, 1H), 6.81–6.73 (m, 2H), 5.66 (s, 2H), 4.23–4.15 (m, 2H), 4.00–3.85 (m, 4H), 3.42 (br s, 2H), 1.84–1.64 (m, 6H), 1.45 (br s, 4H). LC–MS: 482.1 ($[M+H]^+$). HPLC RT: 1.93 min, purity 95.5%.

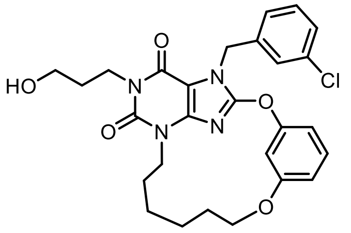

(*E*)-1^7-(3-Chlorobenzyl)-1^1-(3-hydroxypropyl)-1^2,1^3,1^6,1^7-tetrahydro-1^1*H*-2,4-dioxa-1(8,3)-purina-3(1,3)-benzenacyclodecaphane-1^2,1^6-dione (16). Compound **16** was prepared using a similar procedure to that described above for the synthesis of **12** by **42e**. The residue was purified by prep-HPLC (column: Welch Ultimate C18 150 mm × 25 mm × 5 μm; mobile phase: [water (TFA)-ACN]; B%: 46%–76%, 10 min) to give **16** as a white solid (17.1 mg, 9.5% yield). ^{1}H NMR (400 MHz, DMSO-d_6): δ 7.94 (t, J = 2.25 Hz, 1H), 7.55–7.50 (m, 1H), 7.44–7.34 (m, 3H), 7.32–7.25 (m, 1H), 6.90–6.81 (m, 1H), 6.79–6.73 (m, 1H), 5.45 (s, 2H), 4.49–4.43 (m, 1H), 4.19–4.12 (m, 2H), 3.96–3.89 (m, 4H), 3.47–3.41 (m, 2H), 1.82–1.65 (m, 6H), 1.43 (br d, J = 2.13 Hz, 4H). LC–MS: 525.4 ([M+H]$^+$). HPLC RT: 2.53 min, purity 99.8%.

3.4. General procedures for the synthesis of compound ***17***

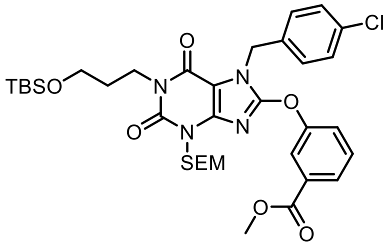

Methyl 3-((1-(3-((*tert*-butyldimethylsilyl)oxy)propyl)-7-(4-chlorobenzyl)-2,6-dioxo-3-((2-(trimethyl silyl)ethoxy)methyl)-2,3,6,7-tetrahydro-1*H*-purin-8-yl)oxy)benzoate (43). To a solution of compound **30** (2.00 g, 3.26 mmol, 1.00 eq.) in DMF (20 mL) was added K_2CO_3 (901 mg, 6.52 mmol, 2.00 eq.) and methyl 3-hydroxybenzoate (495 mg, 3.58 mmol, 1.1 eq.). The mixture was stirred at 80 ℃ for 5 h. The reaction mixture was quenched by addition H_2O 20.0 mL at rt, and then extracted with EtOAc 20.0 mL (10.0 mL × 2). The combined organic layers concentrated under reduced pressure to give compound **43** as a yellow oil (2.00 g, 84.4% yield). LC–MS: 729.2 ([M+H]$^+$).

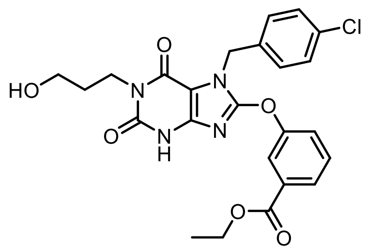

Ethyl 3-((7-(4-chlorobenzyl)-1-(3-hydroxypropyl)-2,6-dioxo-2,3,6,7-tetrahydro-1*H*-purin-8-yl)oxy) benzoate (44). To a solution of compound **43** (2.00 g, 2.80 mmol, 1.00 eq.) in EtOH (20.0 mL) was added HCl (12 mol/L, 2 mL). The mixture was stirred at 80 ℃ for 16 h. Concentrated the reaction mixture under reduced pressure to give a residue. The residue was purified by prep-TLC (Petroleum ether/Ethyl acetate=1/1) to give compound **44** as a white solid (300 mg, 21.5% yield). LC–MS: 499.1 ([M+H]$^+$).

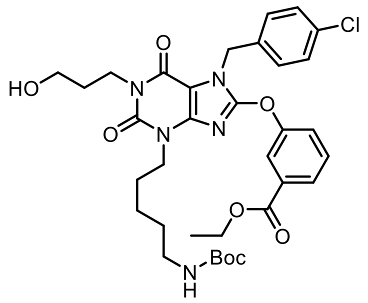

Ethyl 3-((3-(5-((*tert*-butoxycarbonyl)amino)pentyl)-7-(4-chlorobenzyl)-1-(3-hydroxypropyl)-2,6-dioxo-2,3,6,7-tetrahydro-1*H*-purin-8-yl)oxy)benzoate (45). To a solution of compound **44** (300 mg, 601 μmol, 1.00 eq.) in DMF (2 mL) was added *tert*-butyl (5-bromopentyl)carbamate (192 mg, 722 μmol, 1.20 eq.), Cs_2CO_3 (392 mg, 1.20 mmol, 2.00 eq.). The mixture was stirred at 80 ℃ for 3 h. The reaction mixture was quenched by addition H_2O 20.0 mL at rt, and then extracted with EtOAc 10.0 mL. The combined organic layers concentrated under reduced pressure to give a residue. The residue was purified by prep-TLC (Petroleum ether/Ethyl acetate = 1/1) to give **45** as a colorless oil (240 mg, 58.3% yield). LC–MS: 684.2 ([M+H]$^+$).

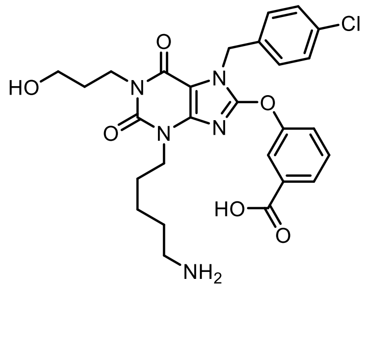

3-((3-(5-Aminopentyl)-7-(4-chlorobenzyl)-1-(3-hydroxypropyl)-2,6-dioxo-2,3,6,7-tetrahydro-1*H*-purin-8-yl)oxy)benzoic acid (46). To a solution of compound **45** (240 mg, 351 μmol, 1.00 eq.) in MeOH (2.00 mL), THF (2.00 mL), H_2O (2 mL) was added LiOH·H_2O (22.4 mg, 534 μmol, 2.00 eq.). The mixture was stirred at rt for 1 h. The reaction mixture was quenched by addition aq $KHSO_4$ 10 mL at rt, and then extracted with EtOAc 5.00 mL. The combined organic layers concentrated under reduced pressure to give a residue. The residue was solute in EtOAc (3.00 mL) and added HCl/EtOAc (4 mol/L, 2 mL). The mixture was stirred at rt for 2 h. Concentrated the reaction mixture to give compound **46** as a white solid (100 mg, 47.0% yield). LC–MS: 556.2 ($[M+H]^+$).

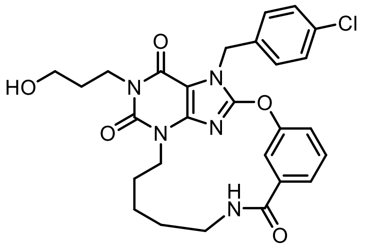

(*E*)-1^7-(4-Chlorobenzyl)-1^1-(3-hydroxypropyl)-1^2,1^3,1^6,1^7-tetrahydro-1^1*H*-2-oxa-5-aza-1(8,3)-purina-3(1,3)-benzenacyclodecaphane-1^2,1^6,4-trione (17). To a solution of compound **46** (200 mg, 338 μmol, 1.00 eq, HCl) in DCM (3 mL) was added DIEA (131 mg, 1.01 mmol, 3.00 eq.) and T_3P (1 g, 1.57 mmol, 935 μL, 50% purity, 4.66 eq.). The mixture was stirred at rt for 2 h. The reaction mixture was quenched by addition H_2O 20.0 mL at rt, and then extracted with EtOAc 10.0 mL. The combined organic layers concentrated under reduced pressure to give a residue. The residue was purified by prep-HPLC (column: Welch Ultimate C18 150 mm × 30 mm × 5 μm; mobile phase: [water (TFA)-ACN]; B%: 26%–56%, 10 min) to give compound **17** as a white solid (44.2 mg, 24.3% yield). ^{1}H NMR (400 MHz, DMSO-d_6): δ 8.69 (br s, 1H), 8.06 (br s, 1H), 7.61–7.41 (m, 7H), 5.45 (s, 2H), 4.47 (t, J = 5.1 Hz, 1H), 4.01–3.83 (m, 4H), 3.45 (q, J = 6.0 Hz, 2H), 3.30–3.24 (m, 2H), 1.79–1.65 (m, 4H), 1.53 (br d, J = 4.9 Hz, 2H), 1.49–1.37 (m, 2H). LC–MS: 556.2 ($[M+H]^+$). HPLC RT: 2.06 min, purity 99.7%.

3.5. General procedures for the synthesis of compound ***18***

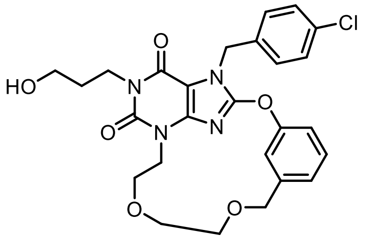

(*E*)-1^{7}-(4-Chlorobenzyl)-1^{1}-(3-hydroxypropyl)-1^{2},1^{3},1^{6},1^{7}-tetrahydro-1^{1}*H*-2,5,8-trioxa-1(8,3)-purina-3(1,3)-benzenacyclodecaphane-1^{2},1^{6}-dione (18). To a solution of compound **47** (100 mg, 270.85 μmol, 1 eq.) in DMF (5.0 mL) was added K_2CO_3 (74.87 mg, 541.71 μmol, 2 eq.) and 3-[2-(2-bromoethoxy)ethoxymethyl]phenol (89.43 mg, 325.03 μmol, 1.2 eq.). The mixture was stirred at 80 ℃ for 16 h. The reaction mixture was diluted with H_2O 10 mL and extracted with ethyl acetate 15 mL (5 mL × 3). The combined organic layers were concentrated under reduced pressure to give a residue. The residue was purified by prep-HPLC (TFA condition; column: Welch Xtimate C18 150 mm × 25 mm × 5 μm; mobile phase: [water(TFA)-ACN]; gradient: 35%–65% B over 10 min) to give compound **18** as a white solid (40 mg, 27.9% yield). ^{1}H NMR (400 MHz, DMSO-d_6): δ 7.57–7.50 (m, 1H), 7.42–7.33 (m, 5H), 7.23–7.16 (m, 1H), 7.02 (d, J = 7.50 Hz, 1H), 5.43–5.36 (m, 2H), 4.50–4.46 (m, 2H), 4.13–4.08 (m, 2H), 3.94 (br t, J = 7.25 Hz, 2H), 3.62 (br t, J = 4.25 Hz, 2H), 3.51–3.44 (m, 6H), 3.43 (br s, 1H), 1.71 (quin, J = 6.82 Hz, 2H). LC–MS: 527.1 ([M+H]$^{+}$). HPLC RT: 2.27 min, purity 99.6%.

3.6. General procedures for the synthesis of compound ***19***

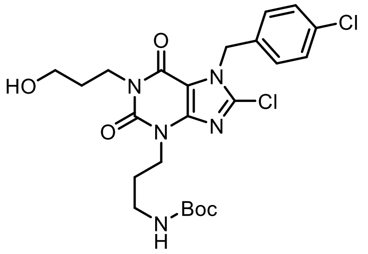

***tert*-Butyl (3-(8-chloro-7-(4-chlorobenzyl)-1-(3-hydroxypropyl)-2,6-dioxo-1,2,6,7-tetrahydro-3*H*-purin-3-yl)propyl)carbamate (48).** To a solution of compound **47** (500 mg, 1.35 mmol, 1.00 eq.) in DMF (5.00 mL) was added *tert*-butyl (3-bromopropyl)carbamate (387 mg, 1.63 mmol, 1.20 eq.), Cs_2CO_3 (883 mg, 2.71 mmol, 2.00 eq.). The mixture was stirred at 80 ℃ for 1 h. The reaction mixture was quenched by addition H_2O 20.0 mL at rt, and then extracted with EtOAc 10.0 mL. The combined organic

layers concentrated under reduced pressure to give compound **48** as a yellow oil (700 mg, 98.8% yield). LC–MS: 526.1 ($[M+H]^+$).

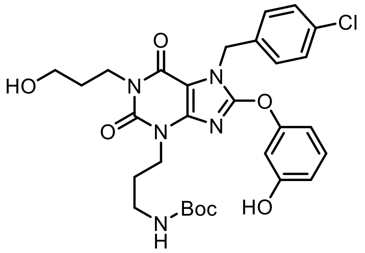

***tert*-Butyl (3-(7-(4-chlorobenzyl)-8-(3-hydroxyphenoxy)-1-(3-hydroxypropyl)-2,6-dioxo-1,2,6,7-tetrahydro-3*H*-purin-3-yl)propyl)carbamate (49).** To a solution of compound **48** (200 mg, 380 μmol, 1.00 eq.) in MeCN (2.00 mL) was added K_2CO_3 (105 mg, 760 μmol, 2.00 eq.), resorcinol (62.8 mg, 570 μmol, 95.1 μL, 1.50 eq.). The mixture was stirred at 80 ℃ for 2 h. The reaction mixture was quenched by addition H_2O 20.0 mL at rt, and then extracted with EtOAc 10.0 mL to give compound **49** as a yellow oil (200 mg, 87.7% yield). LC–MS: 600.3 ($[M+H]^+$).

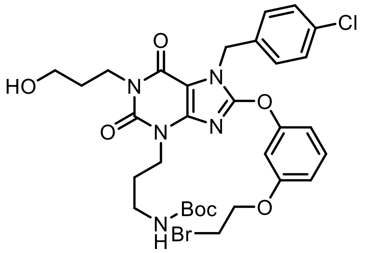

***tert*-Butyl (3-(8-(3-(2-bromoethoxy)phenoxy)-7-(4-chlorobenzyl)-1-(3-hydroxypropyl)-2,6-dioxo-1,2,6,7-tetrahydro-3*H*-purin-3-yl)propyl)carbamate (50).** To a solution of compound **49** (200 mg, 333 μmol, 1.00 eq.) in MeCN (3.00 mL) was added K_2CO_3 (92.1 mg, 667 μmol, 2.00 eq.), 1,2-dibromoethane (75.1 mg, 400 μmol, 30.2 μL, 1.20 eq.). The mixture was stirred at 80 ℃ for 16 h. The reaction mixture was quenched by addition H_2O 15.0 mL at rt, and then extracted with EtOAc 10.0 mL (5.00 mL × 2). The combined organic layers concentrated under reduced pressure to give a residue. The residue was purified by prep-TLC (Petroleum ether/Ethyl acetate = 1/2) to give compound **50** as a white solid (120 mg, 50.9% yield). LC–MS: 708.1 ($[M+H]^+$).

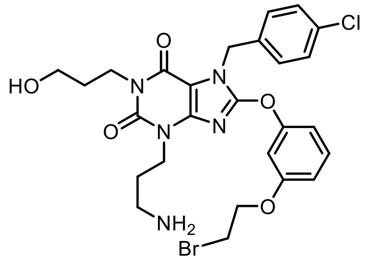

3-(3-Aminopropyl)-8-(3-(2-bromoethoxy)phenoxy)-7-(4-chlorobenzyl)-1-(3-hydroxypropyl)-3,7-dihydro-1*H*-purine-2,6-dione (51). To a solution of compound **50** (100 mg, 141 μmol, 1.00 eq.) in DCM (2.00 mL) was added HCl/dioxane (4 mol/L, 1 mL). The mixture was stirred at rt for 2 h. Concentrated the reaction mixture to give compound **51** as a white solid (80 mg, 94.1% yield). LC–MS: 608.2 ($[M+H]^+$).

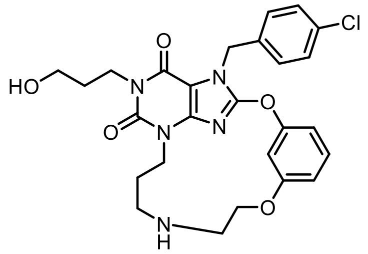

(*E*)-1^7-(4-Chlorobenzyl)-1^1-(3-hydroxypropyl)-1^2,1^3,1^6,1^7-tetrahydro-1^1*H*-2,4-dioxa-7-aza-1(8,3)-purina-3(1,3)-benzenacyclodecaphane-1^2,1^6-dione (19). To a solution of compound **51** (50.0 mg, 82.4 μmol, 1.00 eq.) in MeCN (5.00 mL) was added K_2CO_3 (45.6 mg, 330 μmol, 4.00 eq.). The mixture was stirred at 50 ℃ for 16 h. The reaction mixture was quenched by addition H_2O 15 mL at rt, and then extracted with EtOAc 10.0 mL. The combined organic layers concentrated under reduced pressure to give a residue. The residue was purified by prep-HPLC (column: Welch Xtimate C18 150 mm × 25 mm × 5 μm; mobile phase: [water (TFA)-ACN]; gradient: 20%–50% B over 10 min) to give compound **19** as a white solid (12.4 mg, 27.4% yield). ^{1}H NMR (400 MHz, DMSO-d_6): δ 8.74 (br s, 2H), 7.70 (s, 1H), 7.54 (s, 4H), 7.45 (t, J = 8.2 Hz, 1H), 7.06–7.00 (m, 1H), 6.96 (dd, J = 1.9, 8.4 Hz, 1H), 5.51 (s, 2H), 4.63–4.44 (m, 3H), 4.07 (br t, J = 6.8 Hz, 2H), 4.00 (br t, J = 7.3 Hz, 2H), 3.52 (br d, J = 2.5 Hz, 2H), 3.36 (br s, 2H), 3.21 (br t, J = 6.0 Hz, 2H), 2.04 (br t, J = 6.7 Hz, 2H), 1.82–1.72 (m, 2H). LC–MS: 526.3 ($[M+H]^+$). HPLC RT: 1.67 min, purity 95.4%.

*3.7. General procedures for the synthesis of compounds **20**–**24***

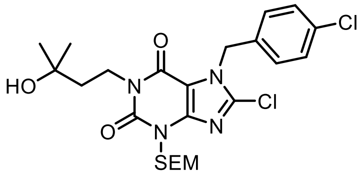

8-Chloro-7-(4-chlorobenzyl)-1-(3-hydroxy-3-methylbutyl)-3-((2-(trimethylsilyl)ethoxy)methyl)-3,7-dihydro-1*H*-purine-2,6-dione (52a). To a solution of **29** (1.5 g, 3.40 mmol, 1.00 eq.) in DMF (15 mL) was added K_2CO_3 (939.36 mg, 6.80 mmol, 2.00 eq.) and 4-bromo-2-methylbutan-2-ol (681.22 mg, 4.08 mmol, 1.20 eq.). The mixture was stirred at 80 ℃ for 3 h. The reaction mixture was partitioned between H_2O 10 mL and EtOAc 20 mL. The organic phase was separated, filtered and concentrated under reduced pressure to give compound **52a** as yellow oil (1.58 g, 88.4% yield). LC–MS: 549.2 ($[M+Na]^+$).

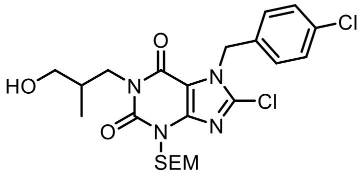

8-Chloro-7-(4-chlorobenzyl)-1-(3-hydroxy-2-methylpropyl)-3-((2-(trimethylsilyl)ethoxy)methyl)-3,7-dihydro-1*H*-purine-2,6-dione (52b). To a solution of compound **29** (1.70 g, 3.85 mmol, 1.00 eq.) in DMF (20.0 mL) was added K_2CO_3 (1.06 g, 7.70 mmol, 2.00 eq.) and 3-bromo-2-methylpropan-1-ol (0.47 g, 3.07 mmol, 0.80 eq.). The mixture was stirred at 80 ℃ for 16 h. The residue was diluted with H_2O 30.0 mL and extracted with Ethyl acetate 60.0 mL (20.0 mL × 3). The combined organic layers were concentrated under reduced pressure to give compound **52b** as a yellow oil (1.80 g, 91.4% yield). LC–MS: 513.1 ($[M+H]^+$).

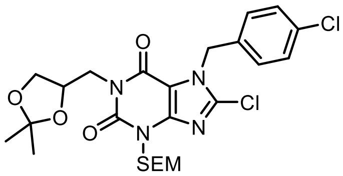

8-Chloro-7-(4-chlorobenzyl)-1-((2,2-dimethyl-1,3-dioxolan-4-yl)methyl)-3-((2-(trimethylsilyl)eth oxy)methyl)-3,7-dihydro-1*H*-purine-2,6-dione (52c). Compound **52c** was prepared using a similar procedure to that described above for the synthesis of **52a** by 4-(bromomethyl)-2,2-dimethyl-1,3-dioxolane (1.90 g, 9.74 mmol, 1.60 eq.) and **29**

(2.69 g, 6.09 mmol, 1 eq.). The combined organic layers were concentrated under reduced pressure to give compound **52c** as a yellow oil (3.4 g, 97.1% yield). LC–MS: 577.3 ($[M+Na]^+$).

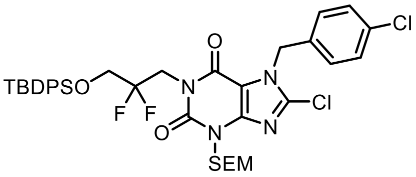

1-(3-((*tert*-Butyldiphenylsilyl)oxy)-2,2-difluoropropyl)-8-chloro-7-(4-chlorobenzyl)-3-((2-(trimethyl silyl)ethoxy)methyl)-3,7-dihydro-1*H*-purine-2,6-dione (52d). Compound **52d** was prepared using a similar procedure to that described above for the synthesis of **52a** by 3-((*tert*-butyldiphenylsilyl)oxy)-2,2-difluoropropyl trifluoromethanesulfonate (2.46 g, 5.10 mmol, 1.50 eq.) and **29** (1.50 g, 3.40 mmol, 1.00 eq.). The reaction mixture was quenched by addition H_2O 15.0 mL, and then extracted with EtOAc 16.0 mL. The combined organic layers were washed with aq. NaCl 5.00 mL, concentrated under reduced pressure to give a residue. The residue was purified by prep-TLC (SiO_2, Petroleum ether: Ethyl acetate = 5:1) to give compound **52d** as a yellow solid (2.40 g, 91.7% yield). LC–MS: 795.2 ($[M+Na]^+$).

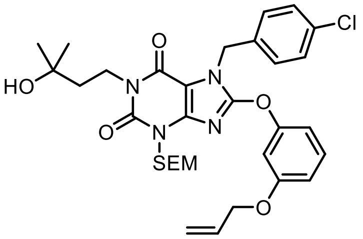

8-(3-(Allyloxy)phenoxy)-7-(4-chlorobenzyl)-1-(3-hydroxy-3-methylbutyl)-3-((2-(trimethylsilyl) ethoxy)methyl)-3,7-dihydro-1*H*-purine-2,6-dione (53a). To a solution of **52a** (2.18 g, 4.13 mmol, 1.00 eq.) and 3-(allyloxy)phenol (1.06 g, 4.96 mmol, 1.20 eq.) in DMF (20 mL) was added K_2CO_3 (1.14 g, 8.27 mmol, 2.00 eq.). The mixture was stirred at 80 ℃ for 16 h. The reaction mixture was partitioned between H_2O 10 mL and EtOAC 10 mL. The organic phase was separated, filtered and concentrated under reduced pressure to give a residue. compound **53a** as brown oil (2.0 g, 75.7% yield). LC–MS: 641.3 ($[M+H]^+$).

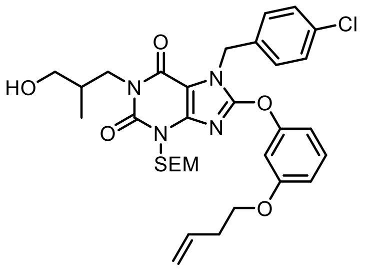

8-(3-(But-3-en-1-yloxy)phenoxy)-7-(4-chlorobenzyl)-1-(3-hydroxy-2-methylpropyl)-3-((2-(trimethyl silyl)ethoxy)methyl)-3,7-dihydro-1*H*-purine-2,6-dione (53b). To a solution of **52b** (2.00 g, 3.89 mmol, 1.00 eq.) in DMF (15.0 mL) was added K_2CO_3 (1.08 g, 7.79 mmol, 2.00 eq.) and 3-(but-3-en-1-yloxy)phenol (770 mg, 4.67 mmol, 1.20 eq.). The mixture was stirred at 80 ℃ for 16 h. The reaction mixture was concentrated under reduced pressure to remove solvent. The residue was diluted with H_2O 20.0 mL and extracted with Ethyl acetate 45.0 mL. The combined organic layers were concentrated under reduced pressure to give compound **53b** as a yellow oil (2.0 g, 80.4% yield). LC–MS: 641.3 ($[M+H]^+$).

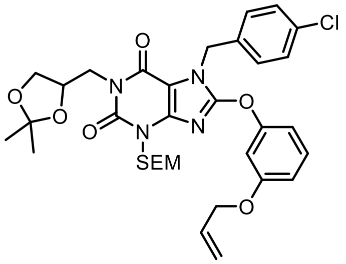

8-(3-(Allyloxy)phenoxy)-7-(4-chlorobenzyl)-1-((2,2-dimethyl-1,3-dioxolan-4-yl)methyl)-3-((2-(tri methylsilyl)ethoxy)methyl)-3,7-dihydro-1*H*-purine-2,6-dione (53c). Compound **53c** was prepared using a similar procedure to that described above for the synthesis of **53a** by **52c** (3.4 g, 6.12 mmol, 1.00 eq.) and 3-(allyloxy)phenol (1.47 g, 9.79 mmol, 1.60 eq.). The reaction mixture was quenched by addition H_2O 30 mL at rt, and then extracted with Ethyl acetate 40 mL. The combined organic layers were concentrated under reduced pressure to give compound **53c** as yellow oil (4.0 g, 97.6% yield). LC–MS: 669.3 ($[M+H]^+$).

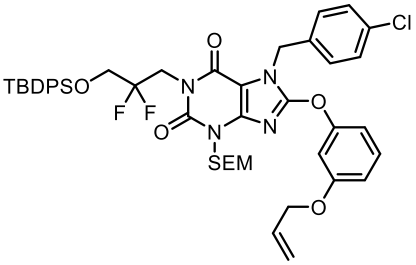

8-(3-(Allyloxy)phenoxy)-1-(3-((*tert*-butyldiphenylsilyl)oxy)-2,2-difluoropropyl)-7-(4-chlorobenzyl)-3-((2-(trimethylsilyl)ethoxy)methyl)-3,7-dihydro-1*H*-purine-2,6-dione (53d). Compound **53d** was prepared using a similar procedure to that described above for the synthesis of **53d** by **52d** (2.40 g, 3.10 mmol, 1.00 eq.) and 3-(allyloxy)phenol (559 mg, 3.72 mmol, 1.20 eq.). The reaction mixture was quenched by addition H_2O 25.0 mL, and then extracted with EtOAc 30.0 mL (15.0 mL × 2). The combined organic layers were washed with aq. NaCl 3.00 mL, concentrated under reduced pressure to give a residue. The residue was purified by column chromatography (SiO_2, Petroleum ether/thyl acetate = 50/1 to 2/1) to give compound **53d** as a yellow oil (600 mg, 21.8% yield). LC–MS: 887.4 ($[M+H]^+$).

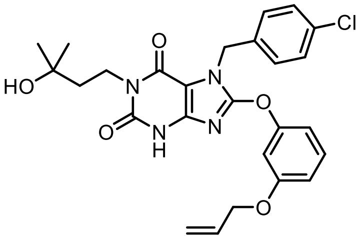

8-(3-(Allyloxy)phenoxy)-7-(4-chlorobenzyl)-1-(3-hydroxy-3-methylbutyl)-3,7-dihydro-1*H*-purine-2,6-dione (54a). To a solution of **53a** (3.6 g, 5.61 mmol, 1.00 eq.) in EtOH (36 mL) was added HCl (12 mol/L, 4 mL, 8.55 eq.) The mixture was stirred at 80 ℃ for 16 h. Filtered and concentrated under reduced pressure to give a residue. The residue was purified by column chromatography (SiO_2, Petroleum ether/Ethyl acetate = 1/1) to give compound **54a** as a white solid (556 mg, 19.4% yield). LC–MS: 533.1 ($[M+H]^+$).

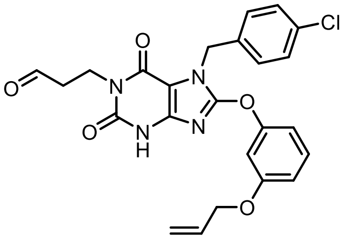

3-(8-(3-(Allyloxy)phenoxy)-7-(4-chlorobenzyl)-2,6-dioxo-2,3,6,7-tetrahydro-1*H*-purin-1-yl)propanal (54b). To a solution of compound **32b** (0.05 g, 103.54 μmol, 1.00 eq.) in DCM (3 mL) was added DMP (52.70 mg, 124.25 μmol, 38.47 μL, 1.20 eq.). The mixture was stirred at rt for 16 h. Concentrated under reduced pressure to give compound **54b** as a yellow solid (60 mg, crude). LC–MS: 481.1 ($[M+H]^+$).

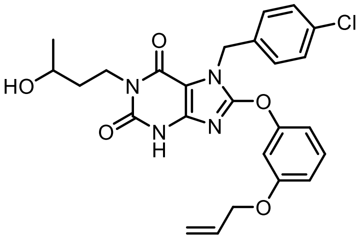

8-(3-(Allyloxy)phenoxy)-7-(4-chlorobenzyl)-1-(3-hydroxybutyl)-3,7-dihydro-1*H*-purine-2,6-dione (54c). To a solution of compound **54b (**60mg**,** 1.00 eq.) in THF (3 mL) was added MeMgBr (3 mol/L, 436.68 μL, 1.50 eq.) at 0 ℃. The mixture was stirred at 0 ℃ for 0.5 h. The reaction mixture was quenched by addition H_2O 20 mL at 0 ℃, and extracted with EtOAc 20 mL (10 mL × 2). The combined organic layers were washed with aq NaCl 5 mL. Concentrated under reduced pressure to give a residue. The residue was purified by prep-TLC (SiO_2, Petroleum ether/Ethyl acetate = 1:2) to give compound **54c** as a yellow solid (180 mg, 40.9% yield). LC–MS: 497.1 ($[M+H]^+$).

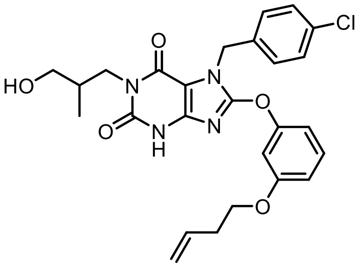

8-(3-(But-3-en-1-yloxy)phenoxy)-7-(4-chlorobenzyl)-1-(3-hydroxy-2-methylpropyl)-3,7-dihydro-1*H*-purine-2,6-dione (54d). To a solution of compound **53b** (0.83 g, 1.29 mmol, 1.00 eq.) in EtOH (15.0 mL) was added HCl (7.5 mL). The mixture was stirred at 80 ℃ for 16 h. The residue was purified by prep-HPLC (TFA condition; column: Phenomenex Luna C18 (250*70mm,10 μm); mobile phase: [water(TFA)-ACN]; gradient: 39%–79% B over 30 min) to give compound **54d** as a white solid (650 mg, 98.8% yield). LC–MS: 511.1 ($[M+H]^+$).

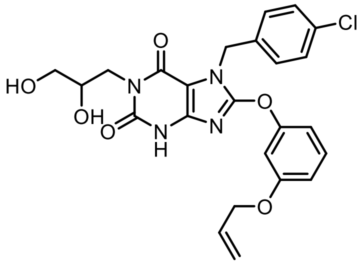

8-(3-(Allyloxy)phenoxy)-7-(4-chlorobenzyl)-1-(2,3-dihydroxypropyl)-3,7-dihydro-1*H*-purine-2,6-dione (54e). To a solution of compound **53c** (4 g, 5.98 mmol, 1.00 eq.) in EtOH (30 mL) was added HCl (4 mol/L, 1.49 mL, 1.00 eq.). The mixture was stirred at 80 °C for 16 h. The reaction mixture was quenched by addition H_2O 30 mL at rt, and then extracted with Ethyl acetate 40 mL. The combined organic layers were concentrated under reduced pressure to give a residue. The residue was purified by column chromatography (SiO_2, Petroleum ether/Ethyl acetate=1/2) to give compound **54e** as white solid (1.48 g, 49.6% yield). LC–MS: 499.3 ($[M+H]^+$).

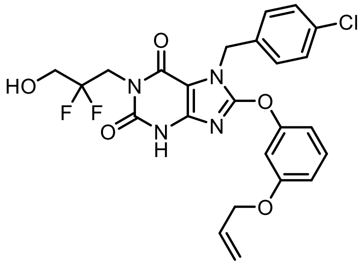

8-(3-(Allyloxy)phenoxy)-7-(4-chlorobenzyl)-1-(2,2-difluoro-3-hydroxypropyl)-3,7-dihydro-1*H*-purine-2,6-dione (54f). To a solution of compound **53d** (600 mg, 676.01 μmol, 1.00 eq.) in EtOH (2.00 mL) was added HCl (12 mol/L, 0.50 mL, 8.88 eq.). The mixture was stirred at 80 ℃ for 16 hrs. LC–MS (EC12352-193-P1A) showed starting material was consumed completely and one main peak with desired *m*/*z*. The reaction mixture was concentrated under reduced pressure to give a residue. The residue was purified by prep-TLC (SiO_2, Petroleum ether: Ethyl acetate = 1:1) to give compound **54f** as a white solid (210 mg, 59.9% yield). LC–MS: 519.1 ($[M+H]^+$).

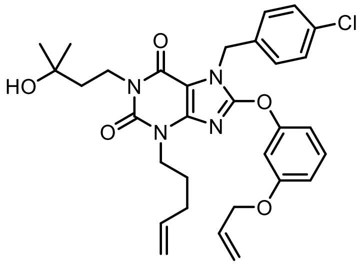

8-(3-(Allyloxy)phenoxy)-7-(4-chlorobenzyl)-1-(3-hydroxy-3-methylbutyl)-3-(pent-4-en-1-yl)-3,7-dihydro-1*H*-purine-2,6-dione (55a). To a solution of **54a** (400 mg, 782.83 μmol, 1.00 eq.) and 5-bromopent-1-ene (140.00 mg, 939.39 μmol, 111.11 μL, 1.20 eq.) in DMF (4 mL) was added Cs_2CO_3 (510.12 mg, 1.57 mmol, 2 eq.). The mixture was stirred at 80 ℃ for 1 h. The reaction mixture was partitioned between H_2O 10 mL and EtOAC 15 mL. The organic phase was separated, filtered and concentrated under reduced pressure to give a residue. The residue was purified by column chromatography (SiO_2, Petroleum ether/Ethyl acetate = 1/1) to give compound **55a** as brown oil (280 mg, 61.8% yield). LC–MS: 579.1 ($[M+H]^+$).

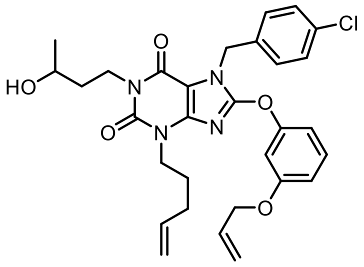

8-(3-(Allyloxy)phenoxy)-7-(4-chlorobenzyl)-1-(3-hydroxybutyl)-3-(pent-4-en-1-yl)-3,7-dihydro-1*H*-purine-2,6-dione (55b). Compound **55b** was prepared using a similar procedure to that described above for the synthesis of **55a** by **54b** (0.18 g, 362.22 μmol, 1.00 eq.) and 5-bromopent-1-ene (80.97 mg, 543.32 μmol, 1.5 eq.). The reaction mixture was quenched by addition H_2O 10 mL at 0 ℃, and extracted with EtOAc 6 mL (3 mL × 2). Concentrated under reduced pressure to give a residue. The residue was purified by prep-TLC (SiO_2, Petroleum ether/Ethyl acetate = 1:2) to give compound **55b** as an off-white solid (110 mg, 52.67% yield). LC–MS: 565.2 ($[M+H]^+$).

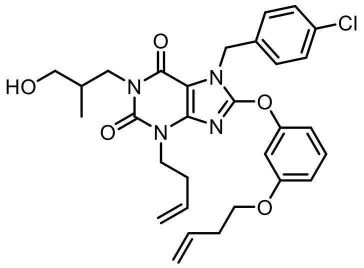

3-(But-3-en-1-yl)-8-(3-(but-3-en-1-yloxy)phenoxy)-7-(4-chlorobenzyl)-1-(3-hydroxy-2-methylpropyl) -3,7-dihydro-1*H*-purine-2,6-dione (55c). To a solution of compound **54c** (600 mg, 1.17 mmol, 1.00 eq.) in DMF (5.00 mL) was added Cs_2CO_3 (765 mg, 2.35 mmol, 2.00 eq.) and 4-bromobut-1-ene-methane (180 mg, 1.21 mmol, 1.20 eq.). The

mixture was stirred at 80 ℃ for 1 h. The residue was diluted with H_2O 10.0 mL and extracted with Ethyl acetate 45.0 mL (15.0 mL × 3). The combined organic layers were concentrated under reduced pressure to give compound **55c** as a yellow oil (600 mg, 90.9% yield). LC–MS: 565.2 ($[M+H]^+$).

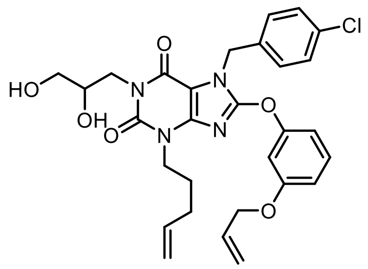

8-(3-(Allyloxy)phenoxy)-7-(4-chlorobenzyl)-1-(2,3-dihydroxypropyl)-3-(pent-4-en-1-yl)-3,7-dihydro-1*H*-purine-2,6-dione (55d). Compound **55d** was prepared using a similar procedure to that described above for the synthesis of **55a** by **54d** (0.5 g, 1.00 mmol, 1.00 eq.) and 5-bromopent-1-ene (224.03 mg, 1.50 mmol, 1.50 eq.). The reaction mixture was quenched by addition H_2O 30 mL at rt, and then extracted with ethyl acetate 30 mL. The combined organic layers were concentrated under reduced pressure to give a residue. The residue was purified by column chromatography (SiO_2, Petroleum ether/Ethyl acetate = 1/2) to give compound **55d** as white solid (412 mg, 72.50% yield). LC–MS: 567.3 ($[M+H]^+$).

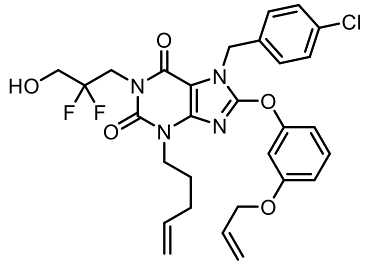

8-(3-(Allyloxy)phenoxy)-7-(4-chlorobenzyl)-1-(2,2-difluoro-3-hydroxypropyl)-3-(pent-4-en-1-yl)-3,7-dihydro-1*H*-purine-2,6-dione (55e). Compound **55e** was prepared using a similar procedure to that described above for the synthesis of **55a** by **54e** (210 mg, 404.71 µmol, 1.00 eq.) and 5-bromopent-1-ene (120.63 mg, 809.41 µmol, 2.00 eq.). The reaction mixture was quenched by addition of H_2O 3.00 mL at rt, and then extracted with EtOAc 4.00 mL (2.00 mL × 2). The combined organic layers were concentrated under reduced pressure to give a residue. The residue was purified by prep-TLC (SiO_2, Petroleum

ether: Ethyl acetate = 2:1) to give compound **55e** as a yellow solid (50.0 mg, 21.1% yield). LC–MS: 587.2 ($[M+H]^+$).

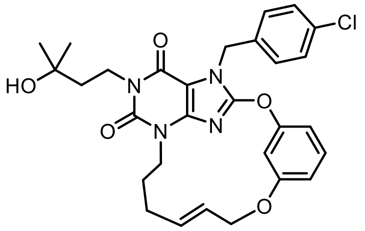

(1^8E,6E)-1^7-(4-Chlorobenzyl)-1^1-(3-hydroxy-3-methylbutyl)-1^2,1^3,1^6,1^7-tetrahydro-1^1H-2,4-dioxa-1(8,3)-purina-3(1,3)-benzenacyclodecaphan-6-ene-1^2,1^6-dione (56a). To a solution of **55a** (280 mg, 483.52 μmol, 1.00 eq.) in DCM (71 mL) was added HG-II (90.90 mg, 145.06 μmol, 0.30 eq.). The mixture was stirred at rt for 16 h. Filtered and concentrated under reduced pressure to give a residue. The residue was purified by column chromatography (SiO_2, Petroleum ether/Ethyl acetate = 1/1) to give compound **56a** as a white solid (190 mg, 71.3% yield). ^{1}H NMR (400 MHz, DMSO-d_6): δ 7.86–7.82 (m, 1H), 7.50–7.41 (m, 4 H), 7.27 (t, J = 8.19 Hz, 1H), 6.84–6.78 (m, 2H), 6.10–5.96 (m, 1H), 5.80–5.60 (m, 1H), 5.43 (s, 2H), 4.70 (br d, J = 5.38 Hz, 2H), 4.00–3.91 (m, 4H), 2.07 (br s, 2H), 1.73 (br d, J = 7.13 Hz, 2H), 1.65–1.54 (m, 2H), 1.15 (s, 6H). LC–MS: 551.1 ($[M+H]^+$).

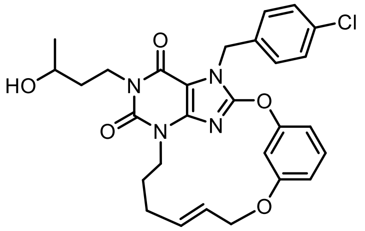

(1^8E,6E)-1^7-(4-Chlorobenzyl)-1^1-(3-hydroxybutyl)-1^2,1^3,1^6,1^7-tetrahydro-1^1H-2,4-dioxa-1(8,3)-purina-3(1,3)-benzenacyclodecaphan-6-ene-1^2,1^6-dione (56b). Compound **56b** was prepared using a similar procedure to that described above for the synthesis of **56a** by **55b** (0.11 g, 194.67 μmol, 1 eq.). The reaction mixture was partitioned between H_2O 100 mL and dichloromethane 60 mL (30 mL × 2). Concentrated under reduced pressure to give a residue. The residue was purified by column chromatography (SiO_2, Petroleum ether/Ethyl acetate=100/1 to 1/2). The residue was purified by prep-HPLC (column: Welch Xtimate C18 150 mm × 25 mm × 5 μm; mobile phase: [water (TFA)-ACN]; B%: 51%–81%,10 min) to give compound **56b** as a white solid (22 mg, 19.8% yield). ^{1}H NMR (400 MHz, DMSO-d_6): δ 7.83 (t, J = 2.25 Hz, 1H), 7.48–7.41 (m, 4H),

7.27 (t, J = 8.19 Hz, 1H), 6.84–6.77 (m, 2H), 6.06–5.95 (m, 1H), 5.71–5.61 (m, 1H), 5.42 (s, 2H), 4.70 (br d, J = 5.63 Hz, 2H), 4.05-3.92 (m, 3H), 3.83 (ddd, J = 12.60, 9.04, 5.75 Hz, 1H), 3.69–3.59 (m, 1H), 2.11–2.02 (m, 2H), 1.78–1.67 (m, 2H), 1.64–1.52 (m, 2H), 1.08 (d, J = 6.13 Hz, 3H). LC–MS: 537.1 ($[M+H]^+$).

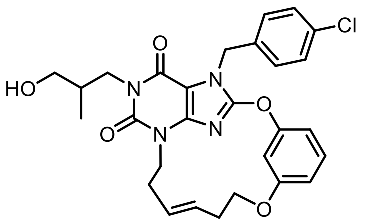

(1^8E,7E)-1^7-(4-Chlorobenzyl)-1^1-(3-hydroxy-2-methylpropyl)-1^2,1^3,1^6,1^7-tetrahydro-1^1H-2,4-dioxa-1(8,3)-purina-3(1,3)-benzenacyclodecaphan-7-ene-1^2,1^6-dione (56c). Compound **56c** was prepared using a similar procedure to that described above for the synthesis of **56a** by **55c** (300 mg, 531 μmol, 1.00 eq.). The residue was purified by prep-HPLC (TFA condition; column: Welch Xtimate C18 150 mm × 25 mm × 5 μm; mobile phase: [water(TFA)-ACN]; gradient: 63%–93% B over 10 min) to give compound **56c** as a white solid (5.41 mg, 18.0% yield). ^{1}H NMR (400 MHz, DMSO-d_6): δ 8.23 (t, J = 2.19 Hz, 1H), 7.52–7.42(m, 4H), 7.31 (t, J = 8.25 Hz, 1H), 6.92–6.72(m, 2H), 5.70–5.53(m, 2H), 5.43 (s, 2H), 4.45–4.37 (m, 1H), 4.19–4.07 (m, 2H), 3.93–3.73 (m, 4H), 3.31–3.20 (m, 2H), 2.62–2.54 (m, 2H), 2.48 (br s, 2H), 2.10–1.94 (m, 1H), 0.82 (d, J = 6.88 Hz, 3H). LC–MS: 537.4 ($[M+H]^+$).

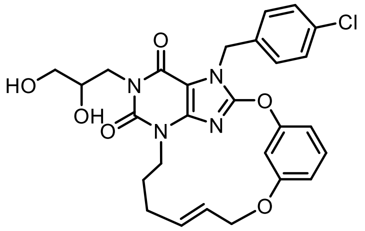

(1^8E,6E)-1^7-(4-Chlorobenzyl)-1^1-(2,3-dihydroxypropyl)-1^2,1^3,1^6,1^7-tetrahydro-1^1H-2,4-dioxa-1(8,3)-purina-3(1,3)-benzenacyclodecaphan-6-ene-1^2,1^6-dione (56d). Compound **56d** was prepared using a similar procedure to that described above for the synthesis of **56a** by **55d** (0.38 g, 670.16 μmol, 1.00 eq.). The residue was purified by prep-HPLC (column: Welch Xtimate C18 150 mm × 25 mm × 5 μm; mobile phase: [water (TFA)-ACN]; B%: 38%–68%, 10 min) to give compound **56d** as a white solid (24.58 mg, 6.8% yield). ^{1}H NMR (400 MHz, DMSO-d_6): δ 7.84 (t, J = 2.31 Hz, 1H), 7.45 (s, 4H), 7.28

(t, J = 8.19 Hz, 1H), 6.88–6.76 (m, 2H), 6.11–5.95 (m, 1H), 5.77–5.58 (m, 1H), 5.43 (s, 2H), 4.71 (br d, J = 5.50 Hz, 2H), 4.65 (d, J = 5.38 Hz, 1H), 4.51 (t, J = 5.75 Hz, 1H), 4.06–3.91 (m, 3H), 3.85–3.76 (m, 2H), 2.15–2.02 (m, 2H), 1.74 (br d, J = 6.63 Hz, 2H). LC–MS: 539.5 ($[M+H]^+$).

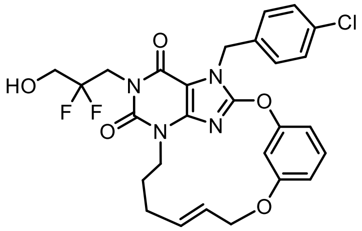

(1^8E,6E)-1^7-(4-Chlorobenzyl)-1^1-(2,2-difluoro-3-hydroxypropyl)-1^2,1^3,1^6,1^7-tetrahydro-1^1H-2,4-dioxa-1(8,3)-purina-3(1,3)-benzenacyclodecaphan-6-ene-1^2,1^6-dione (56e). Compound **56e** was prepared using a similar procedure to that described above for the synthesis of **56a** by **55e** (16.0 mg, 27.26 μmol, 1.00 eq.). The residue was purified by prep-HPLC (column: Waters xbridge 150 mm × 25mm × 10 μm; mobile phase: [water (NH_4HCO_3)-ACN]; gradient: 58%–88% B over 10 min) to give compound **56e** as a black gum (3.28 mg, 20.9% yield). ^{1}H NMR (400 MHz, DMSO-d_6): δ 7.82 (s, 1H), 7.44 (s, 4H), 7.28 (t, J = 8.25 Hz, 1H), 6.81 (br t, J = 8.25 Hz, 2H), 6.05–5.92 (m, 1H), 5.74–5.60 (m, 1H), 5.51 (t, J = 6.44 Hz, 1H), 5.42 (s, 2H), 4.70 (br d, J = 5.38 Hz, 2H), 4.39 (br d, J = 14.38 Hz, 2H), 3.99–3.92 (m, 2H), 3.66–3.60 (m, 2H), 2.10–2.03 (m, 2H), 1.76–1.69 (m, 2H). LC–MS: 559.0 ($[M+H]^+$).

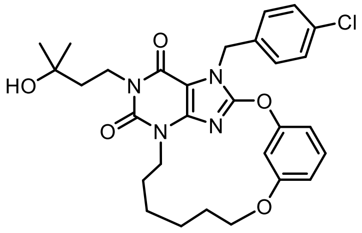

(E)-1^7-(4-Chlorobenzyl)-1^1-(3-hydroxy-3-methylbutyl)-1^2,1^3,1^6,1^7-tetrahydro-1^1H-2,4-dioxa-1(8,3)-purina-3(1,3)-benzenacyclodecaphane-1^2,1^6-dione (20). To a solution of **56a** (0.1 g, 181.48 μmol, 1 eq.), Lindlar (18.15 μmol, 0.1 eq.) (100 mg) in EtOAc (10.0 mL). The suspension was degassed and purged with H_2 for 3 times. The mixture was stirred under H_2 (15 psi) at rt for 0.5 h. Filtered the mixture and the filtrate was concentrated to get the crude product. The residue was purified by prep-HPLC (column: Phenomenex Luna C18 150 mm × 25mm × 10 μm; mobile phase: [water (TFA) -ACN]; gradient: 68%–98%

B over 10 min) to give compound **20** as a white solid (58.58 mg, 58.4% yield). ^{1}H NMR (400 MHz, DMSO-d_6) δ 8.01–7.88 (m, 1H), 7.45 (s, 4H), 7.28 (t, J = 8.25 Hz, 1H), 6.92–6.74 (m, 2H), 5.44 (s, 2H), 4.23–4.11 (m, 2H), 4.03–3.88 (m, 4H), 1.87–1.57 (m, 6H), 1.43 (br s, 4H), 1.15 (s, 6H). LC–MS: 553.4 ([M+H]$^+$). HPLC RT: 2.68 min, purity 99.7%.

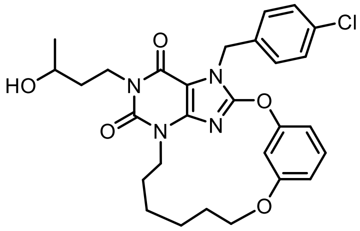

(*E*)-1^7-(4-Chlorobenzyl)-1^1-(3-hydroxybutyl)-1^2,1^3,1^6,1^7-tetrahydro-1^1*H*-2,4-dioxa-1(8,3)-purina-3(1,3)-benzenacyclodecaphane-1^2,1^6-dione (21). Compound **21** was prepared using a similar procedure to that described above for the synthesis of **20** by **56b** (0.05 g, 93.11 μmol, 1.00 eq.). The residue was purified by prep-HPLC (column: Welch Xtimate C18 150 mm × 25 mm × 5 μm; mobile phase: [water (TFA)-ACN]; B%: 52%–82%,10 min) to give compound **21** as a white solid (8 mg, 15.94% yield). ^{1}H NMR (400 MHz, DMSO-d_6): δ 7.93 (t, J = 2.06 Hz, 1H), 7.45 (s, 4H), 7.28 (t, J = 8.19 Hz, 1H), 6.86–6.81 (m, 1H), 6.78–6.73 (m, 1H), 5.44 (s, 2H), 4.21–4.12 (m, 2H), 4.06-3.96 (m, 1H), 3.95–3.79 (m, 3H), 3.71–3.59 (m, 1H), 1.84–1.75 (m, 2H), 1.74–1.67 (m, 2H), 1.63–1.52 (m, 2H), 1.49–1.37 (m, 4H), 1.08 (d, J = 6.13 Hz, 3H). LC–MS: 539.1 ([M+H]$^+$). HPLC RT: 2.94 min, purity 99.9%.

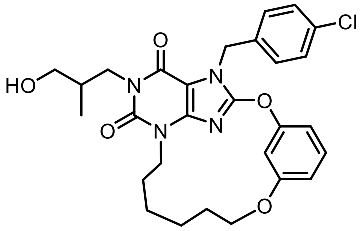

(*E*)-1^7-(4-Chlorobenzyl)-1^1-(3-hydroxy-2-methylpropyl)-1^2,1^3,1^6,1^7-tetrahydro-1^1*H*-2,4-dioxa-1(8,3)-purina-3(1,3)-benzenacyclodecaphane-1^2,1^6-dione (22). Compound **22** was prepared using a similar procedure to that described above for the synthesis of **20** by **56c** (50.0 mg, 93.1 μmol, 1.00 eq.). The residue was purified by prep-HPLC (TFA condition; column: Phenomenex Luna C18 150 mm × 25mm × 10 μm; mobile phase: [water (TFA)-ACN]; gradient: 62%–92% B over 10 min) to give compound **22** as a white solid (5.00 mg, 9.6% yield). ^{1}H NMR (400 MHz, DMSO-d_6): δ 7.94 (t, J = 2.25 Hz, 1H),

7.45 (s, 4H), 7.31–7.26(m, 1H), 6.87–6.83 (m, 1H), 6.76 (dd, *J* = 8.25, 1.63 Hz, 1H), 5.44 (s, 2H), 4.44–4.40 (m, 1H), 4.19–4.13 (m, 2H), 3.95–3.88 (m, 2H), 3.86–3.75 (m, 2H), 3.31–3.19 (m, 2H), 2.01 (dq, *J* = 12.91, 6.62 Hz, 1H), 1.79 (br s, 2H), 1.73–1.66 (m, 2H), 1.43 (br s, 4H), 0.81 (d, *J* = 6.88 Hz, 3H). LC–MS: 539.4 ($[M+H]^+$). HPLC RT: 2.27 min, purity 96.8%.

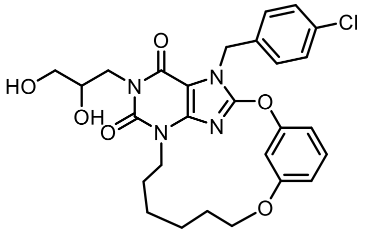

(*E*)-1^7-(4-Chlorobenzyl)-1^1-(2,3-dihydroxypropyl)-1^2,1^3,1^6,1^7-tetrahydro-1^1*H*-2,4-dioxa-1(8,3)-purina -3(1,3)-benzenacyclodecaphane-12,16-dione (23). Compound **23** was prepared using a similar procedure to that described above for the synthesis of **20** by **56d** (0.106 g, 196.67 μmol, 1.00 eq.). The residue was purified by prep-HPLC (column: Welch Xtimate C18 150 mm × 25 mm × 5 μm; mobile phase: [water (TFA)-ACN]; B%: 42%–72%, 10 min) to give compound **23** as a white solid (3.51 mg, 3.3% yield). ^{1}H NMR (400 MHz, DMSO-d_6): δ 7.95 (t, *J* = 2.25 Hz, 1H), 7.45 (s, 4H), 7.29 (t, *J* = 8.25 Hz, 1H), 6.85 (dd, *J* = 8.13, 1.50 Hz, 1H), 6.76 (dd, *J* = 8.32, 1.56 Hz, 1H), 5.45 (s, 2H), 4.74–4.40 (m, 2H), 4.23–4.12 (m, 2H), 4.06–3.97 (m, 1H), 3.96-3.87 (m, 2H), 3.86–3.76 (m, 2H), 1.87–1.65 (m, 4H), 1.43 (br s, 4H). LC–MS: 541.4 ($[M+H]^+$). HPLC RT: 2.38 min, purity 97.3%.

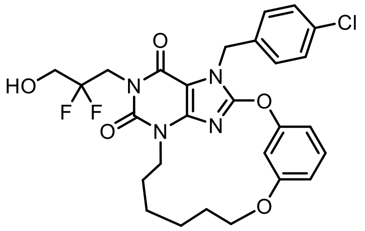

(*E*)-1^7-(4-Chlorobenzyl)-1^1-(2,2-difluoro-3-hydroxypropyl)-1^2,1^3,1^6,1^7-tetrahydro-1^1*H*-2,4-dioxa-1(8,3)-purina -3(1,3)-benzenacyclodecaphane-12,16-dione (24). Compound **24** was prepared using a similar procedure to that described above for the synthesis of **20** by **56e** (14.0 mg, 25.0 μmol, 1.00 eq.). The residue was purified by prep-HPLC (column: Waters xbridge 150 mm × 25mm × 10 μm; mobile phase: [water (NH4HCO3)-ACN]; gradient: 64%–94% B over 10 min) to give compound **24** as a white

solid (2.44 mg, 17.3% yield). ^{1}H NMR (400 MHz, DMSO-d_6): δ 7.93 (br s, 1H), 7.44 (s, 4H), 7.29 (t, J = 8.19 Hz, 1H), 6.85 (br d, J = 7.50 Hz, 1H), 6.79–6.74 (m, 1H), 5.52 (br t, J = 6.00 Hz, 1H), 5.43 (s, 2H), 4.42 (br t, J = 14.38 Hz, 2H), 4.21–4.12 (m, 2H), 3.96–3.88 (m, 2H), 3.65 (td, J = 13.98, 5.82 Hz, 2H), 1.81–1.68 (m, 4H), 1.43 (br s, 4H). LC–MS: 561.3 ($[M+H]^+$). HPLC RT: 2.56 min, purity 99.9%.

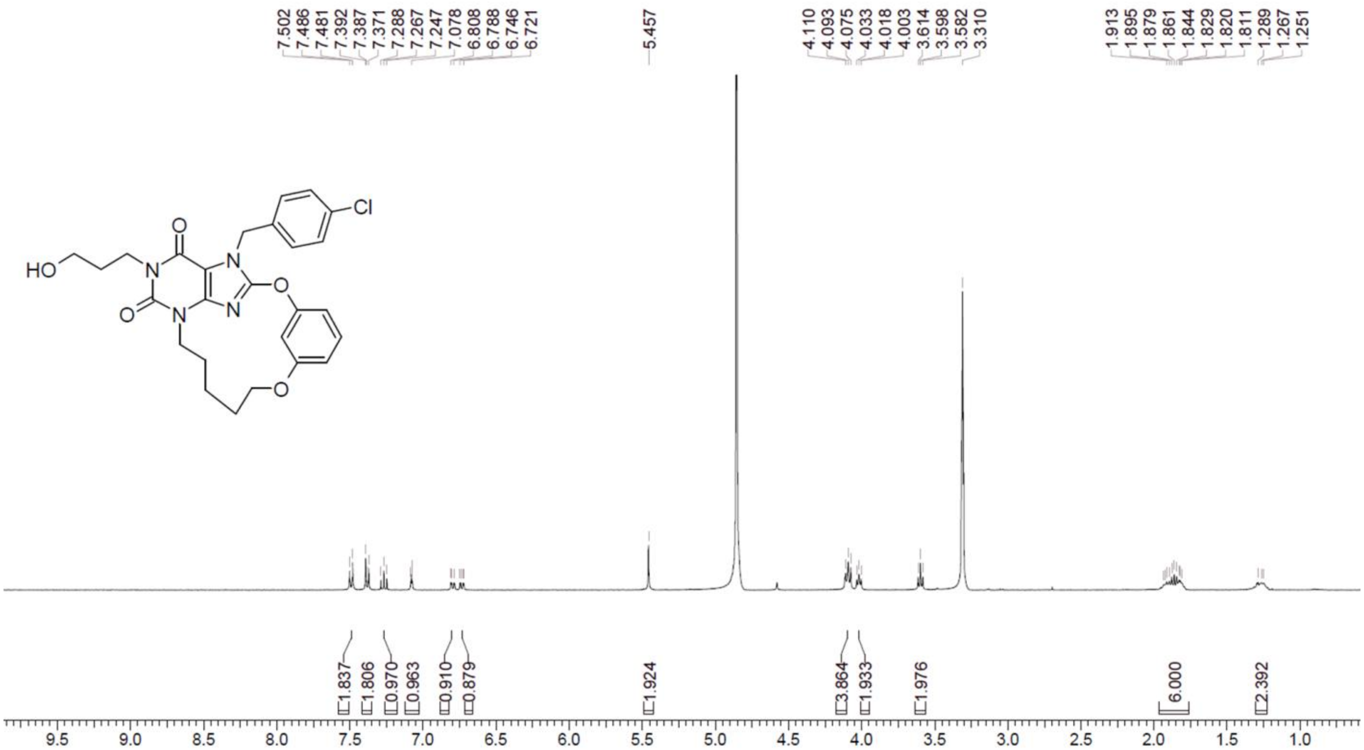

^{1}H NMR of compound **5**

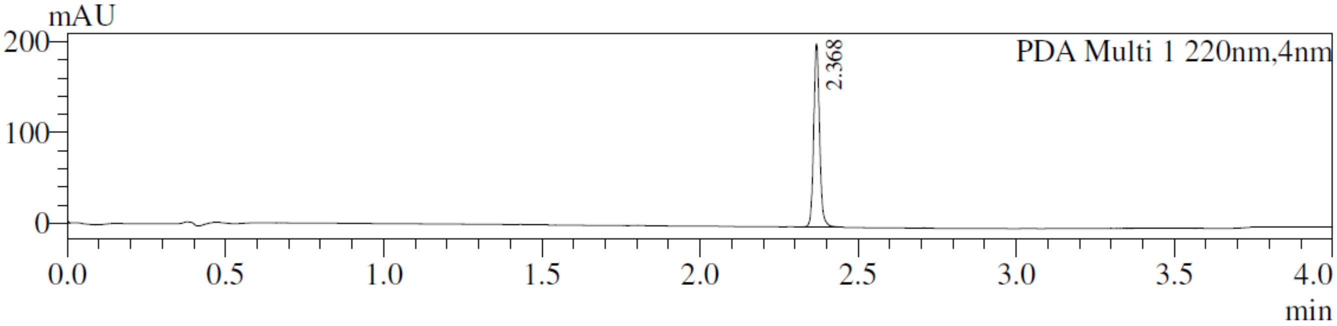

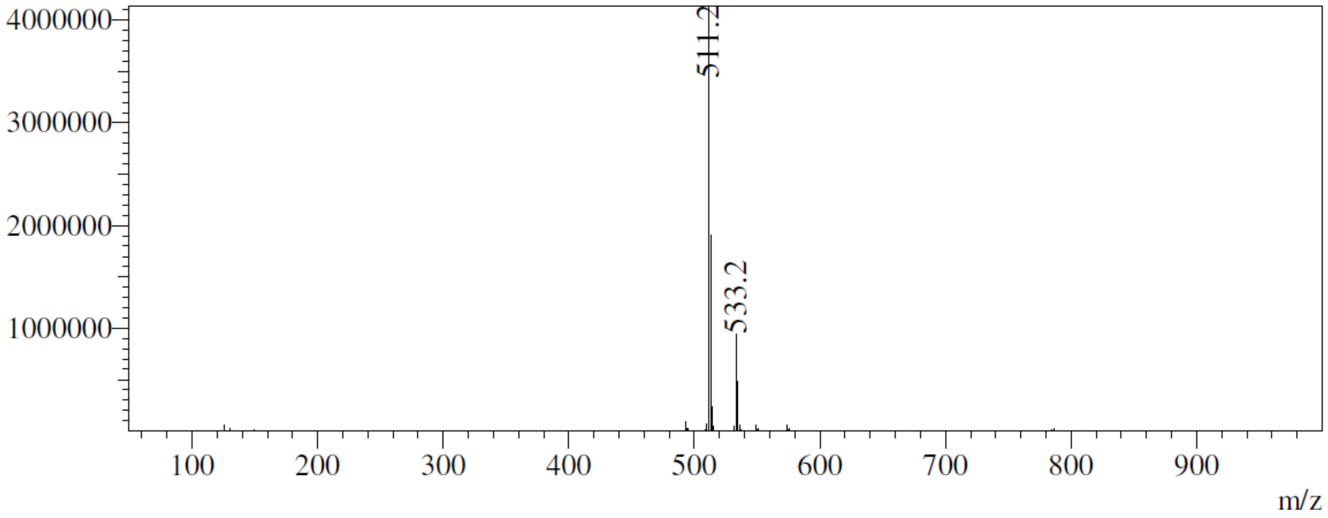

HPLC and MS of compound **5**

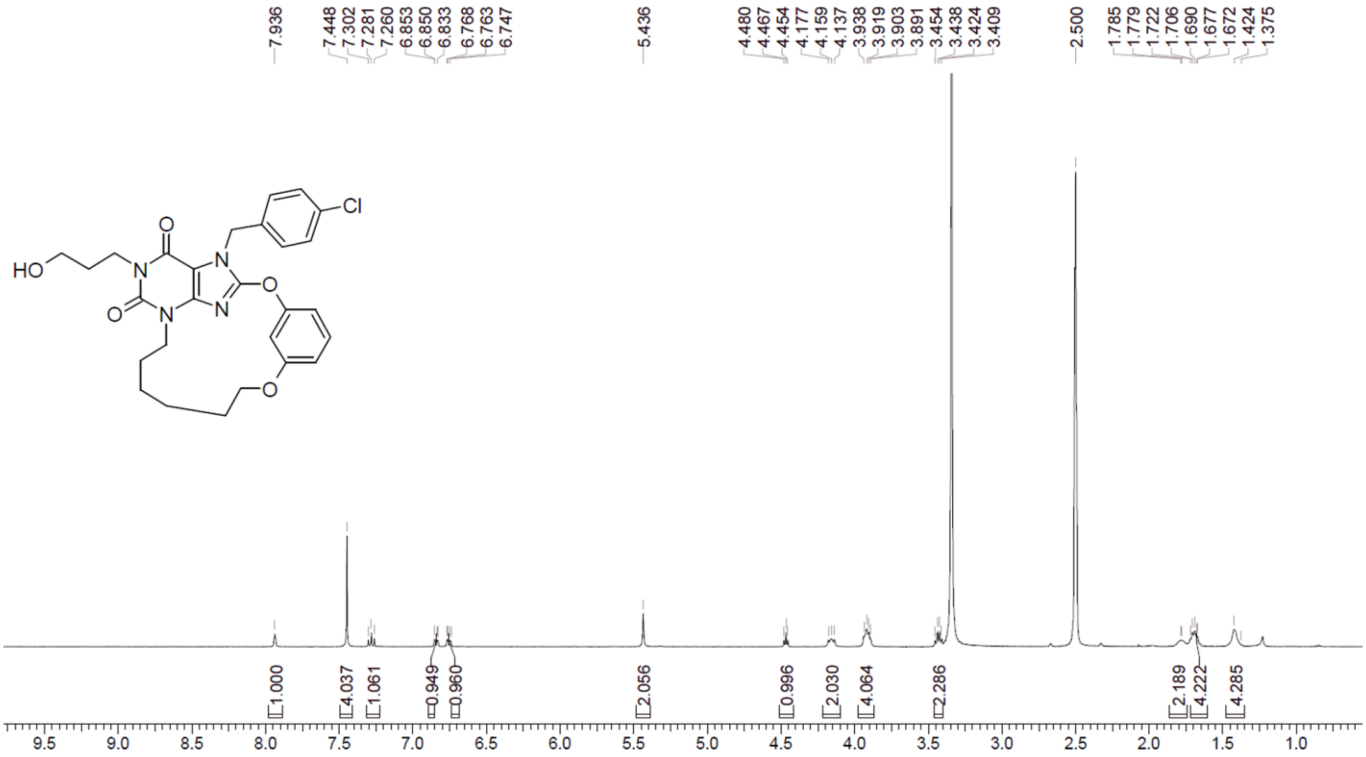

^{1}H NMR of compound **6**

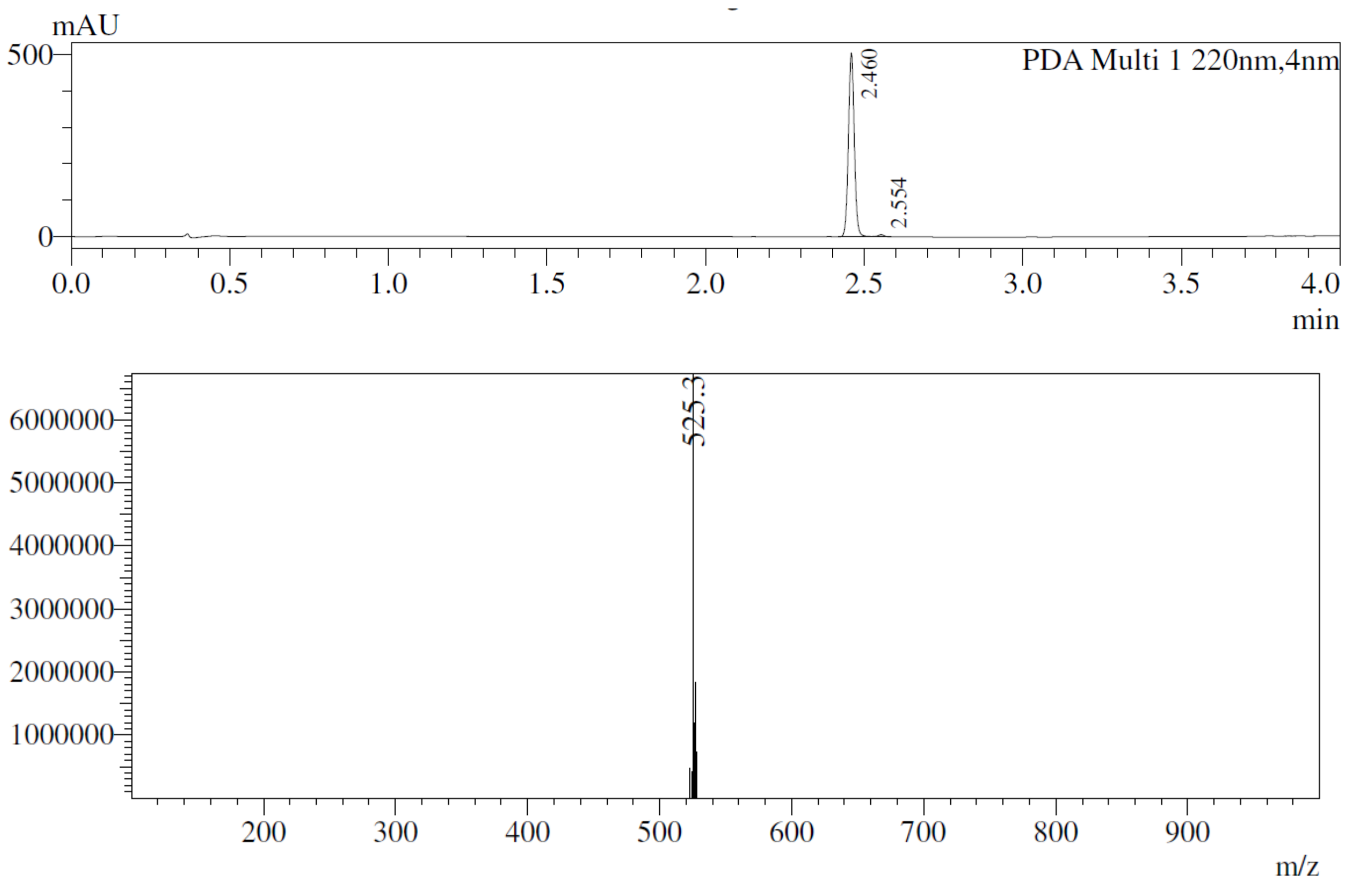

HPLC and MS of compound **6**

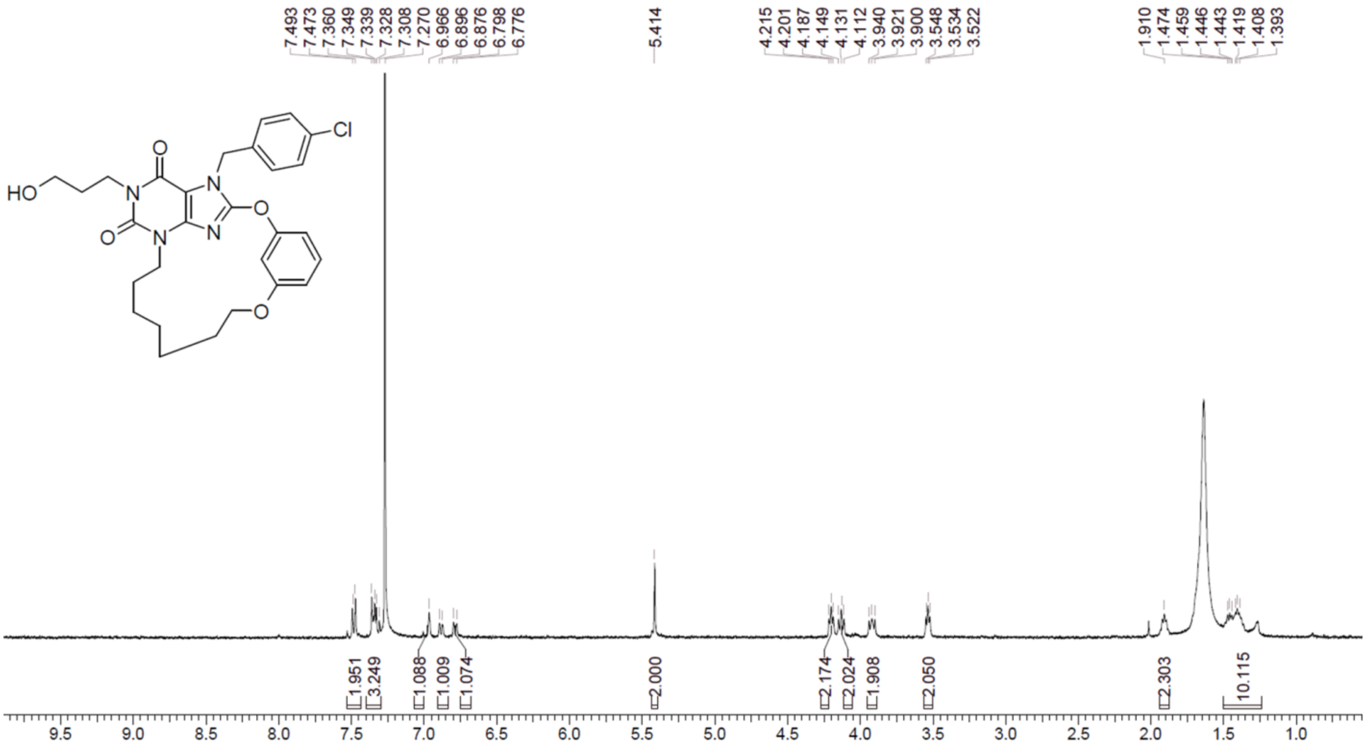

^{1}H NMR of compound **7**

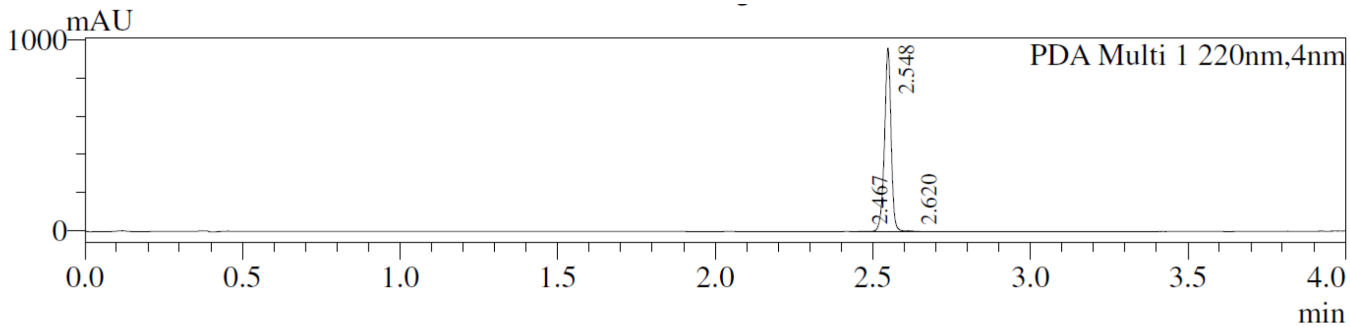

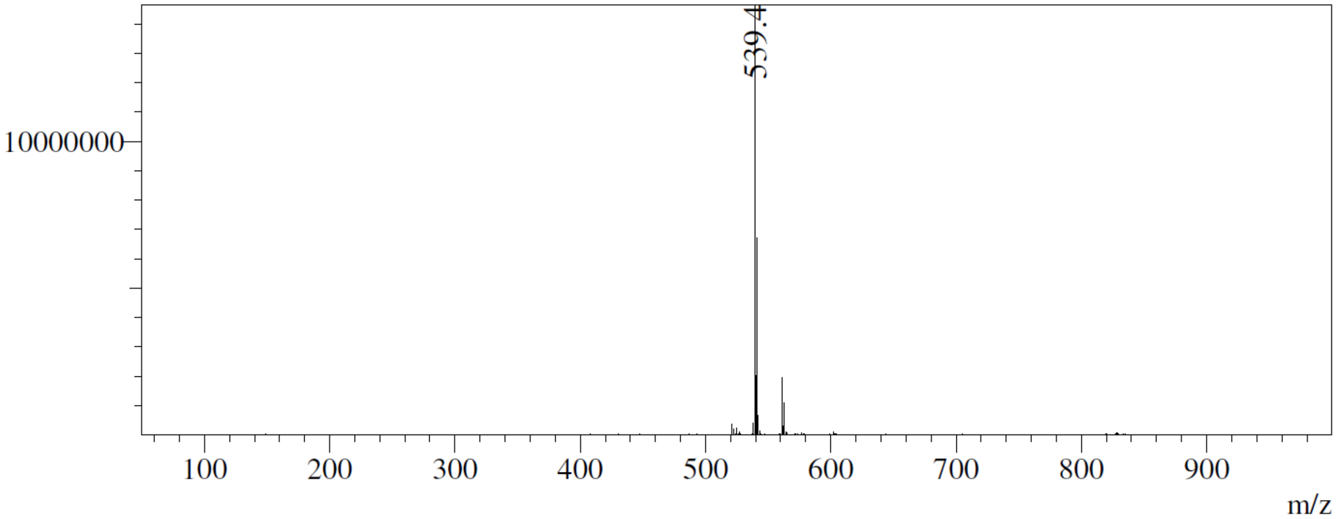

HPLC and MS of compound **7**

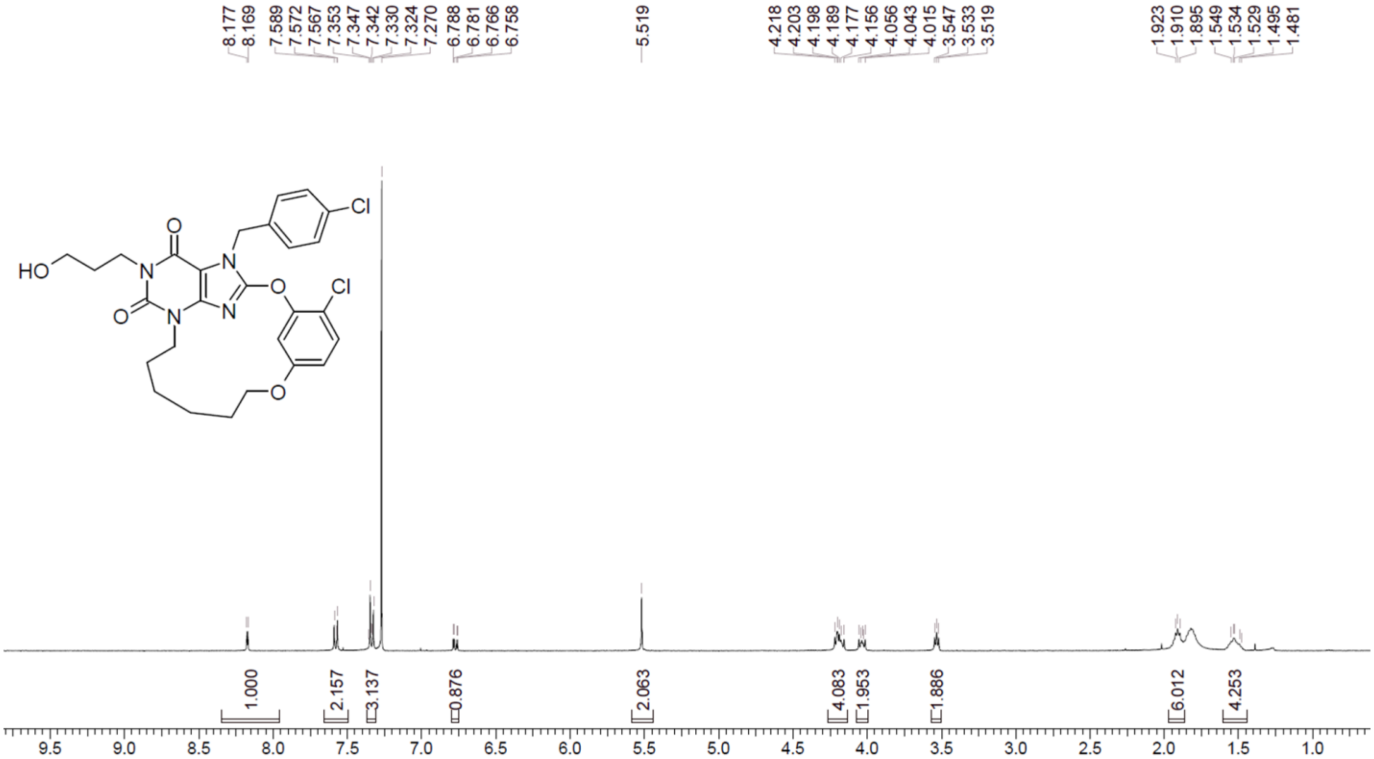

^{1}H NMR of compound **8**

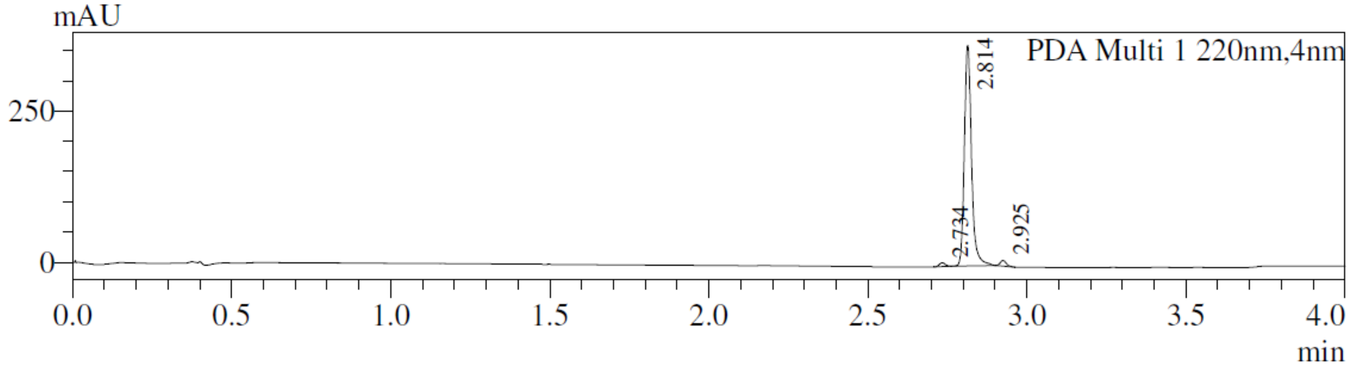

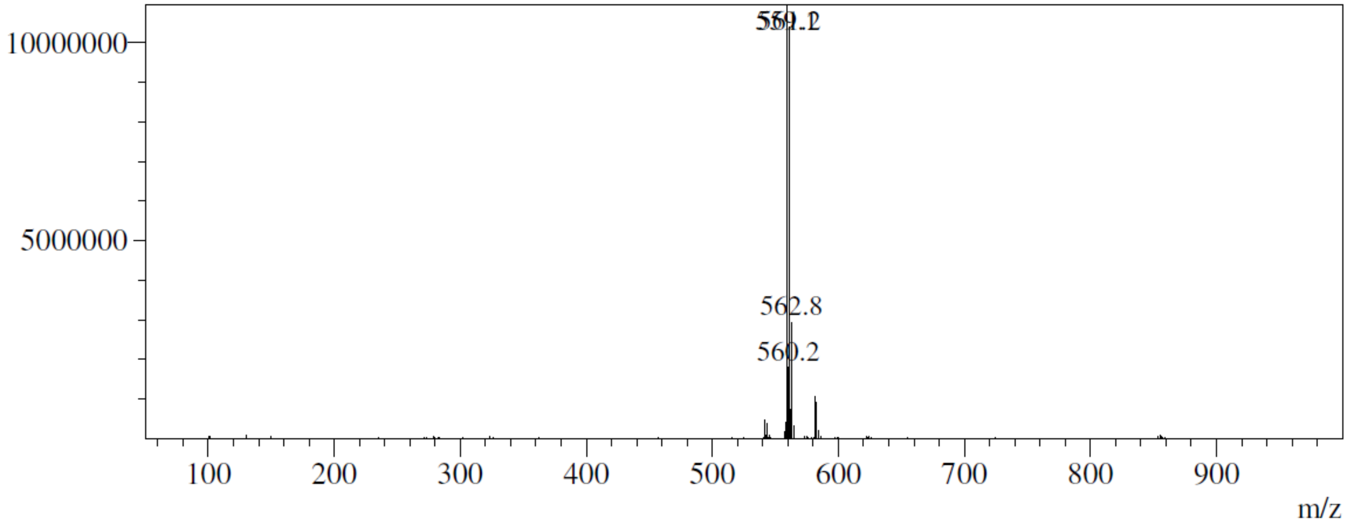

HPLC and MS of compound **8**

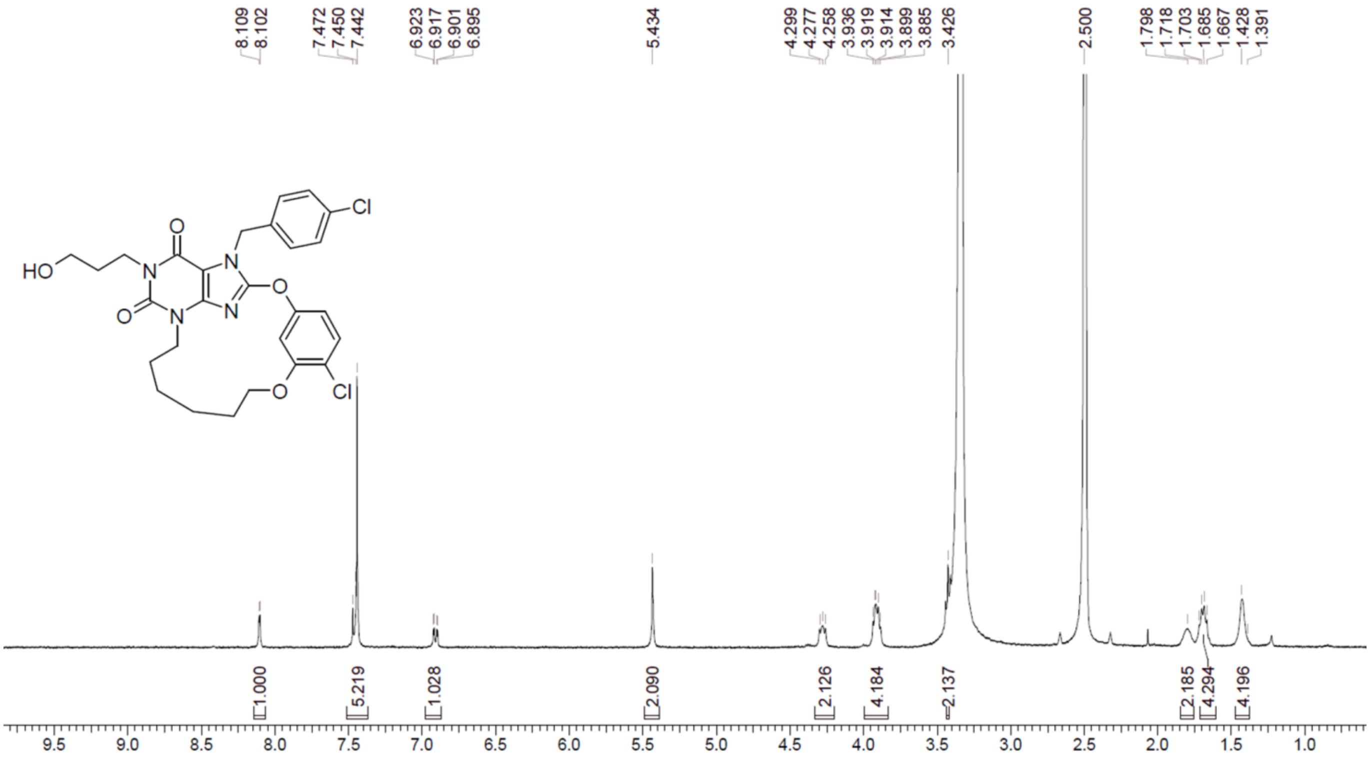

^{1}H NMR of compound **9**

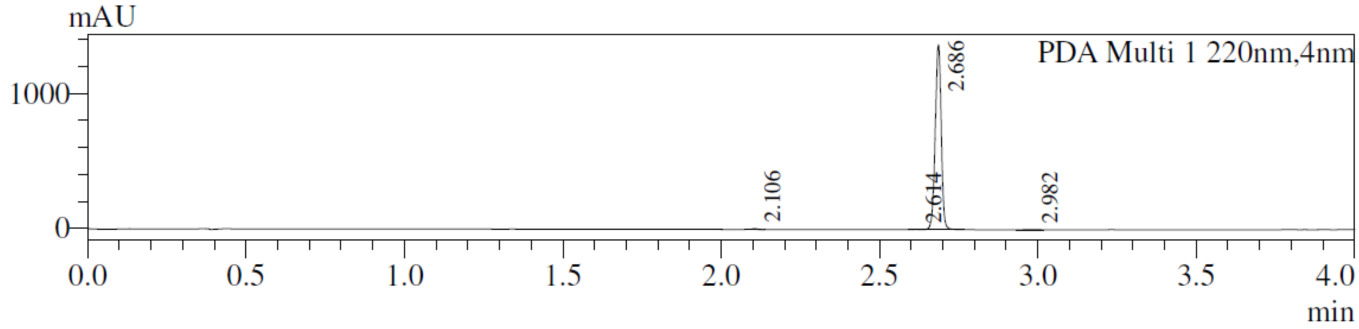

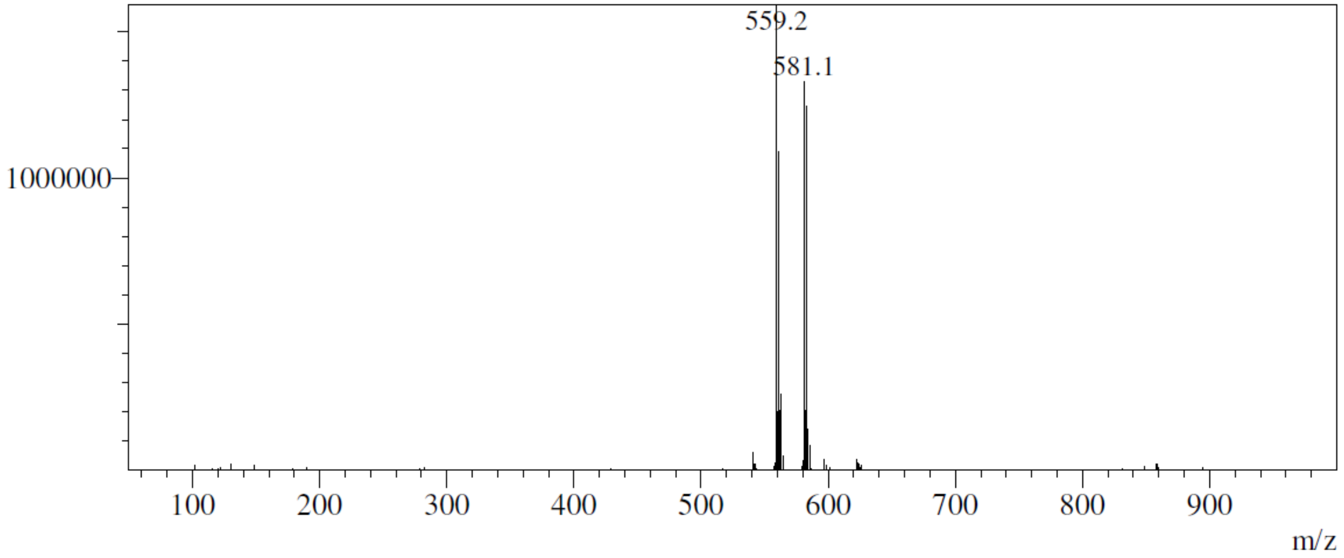

HPLC and MS of compound **9**

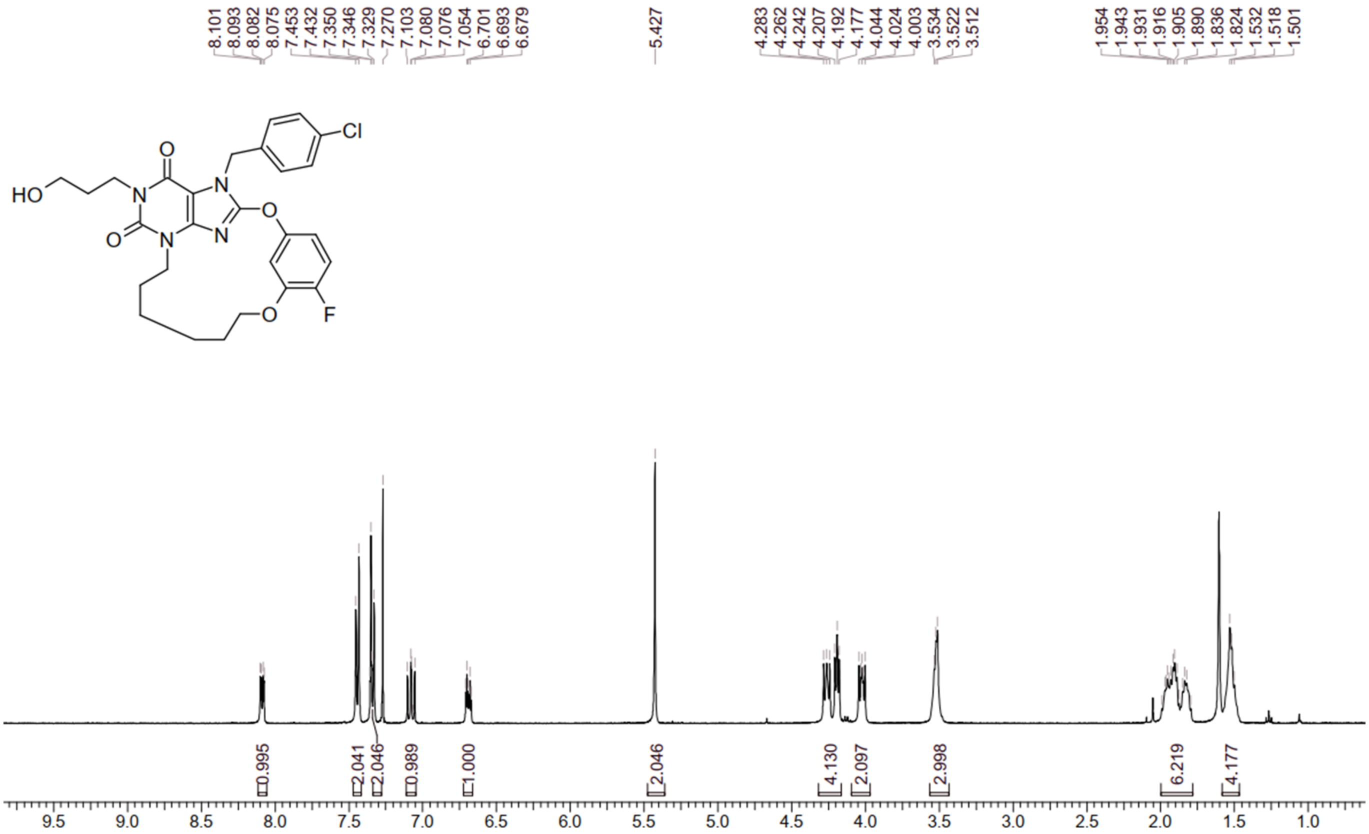

^{1}H NMR of compound **10**

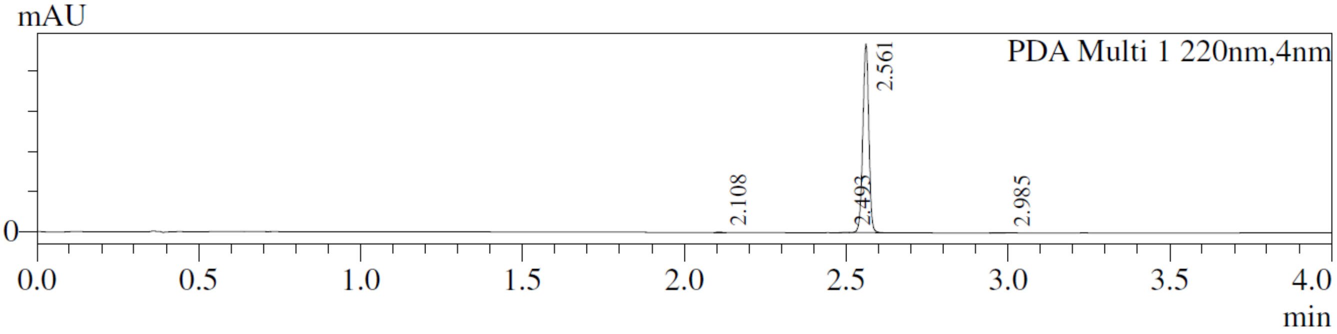

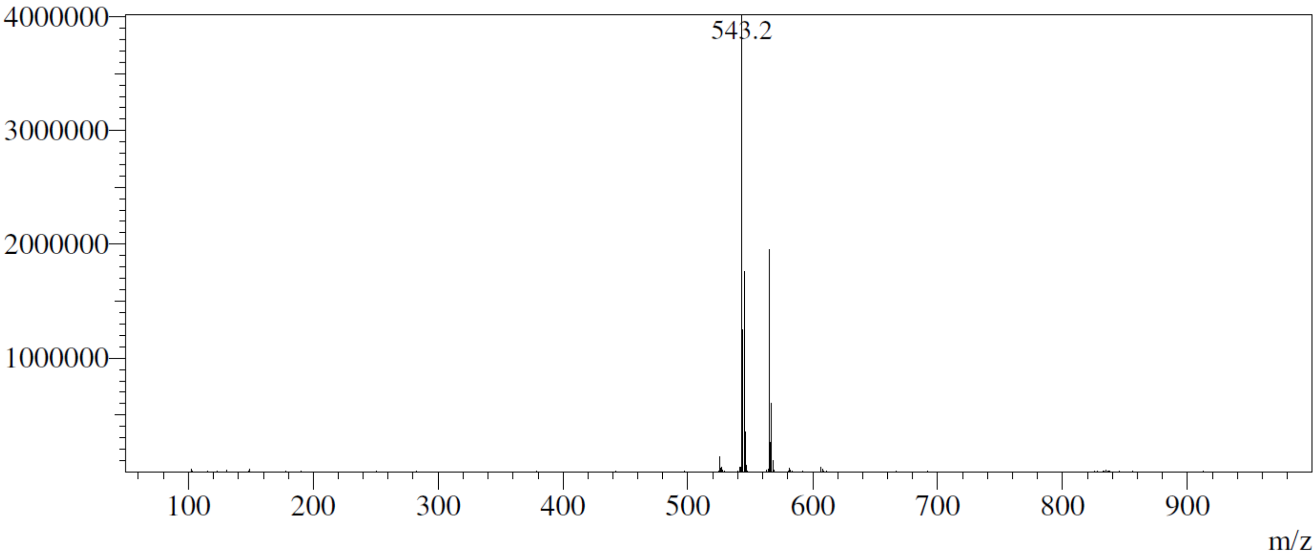

HPLC and MS of compound **10**

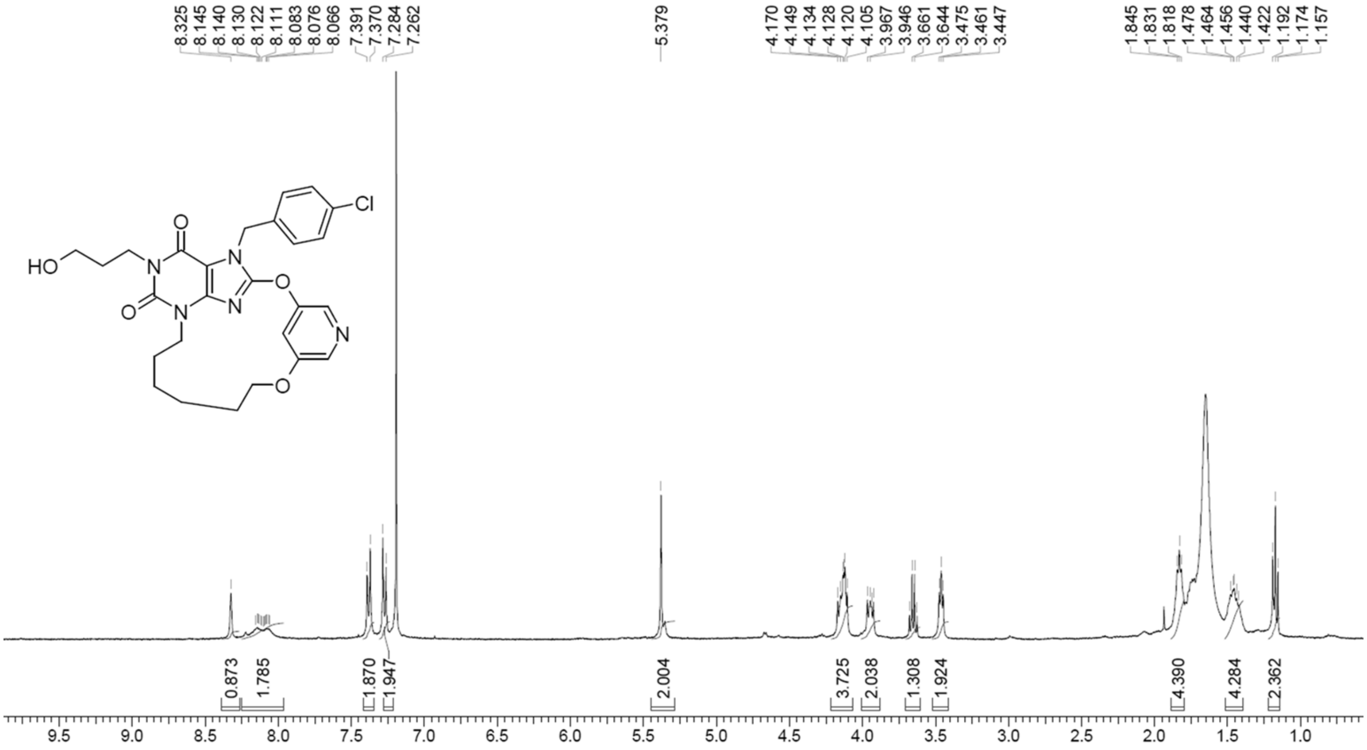

^{1}H NMR of compound **11**

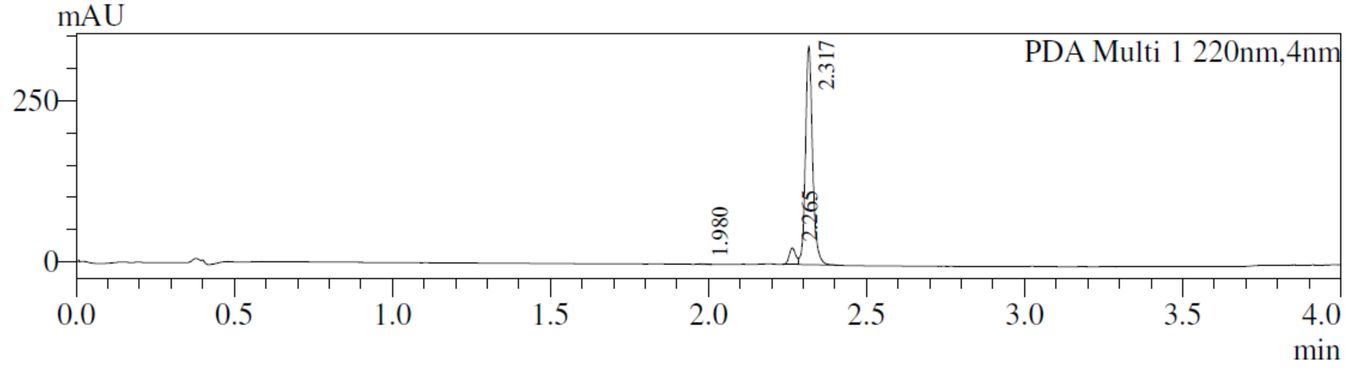

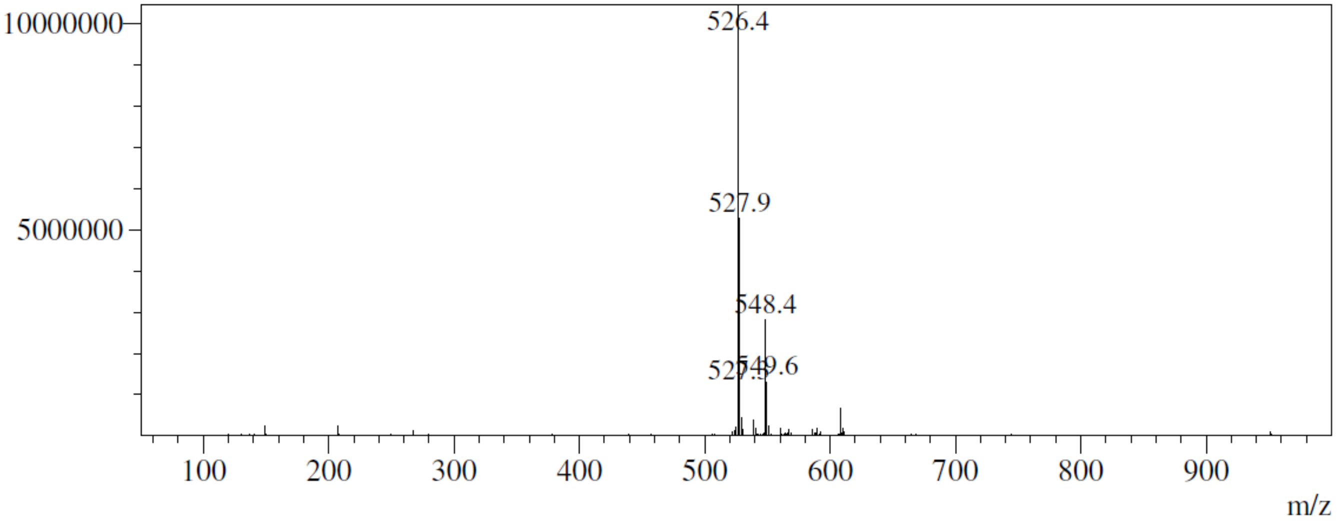

HPLC and MS of compound **11**

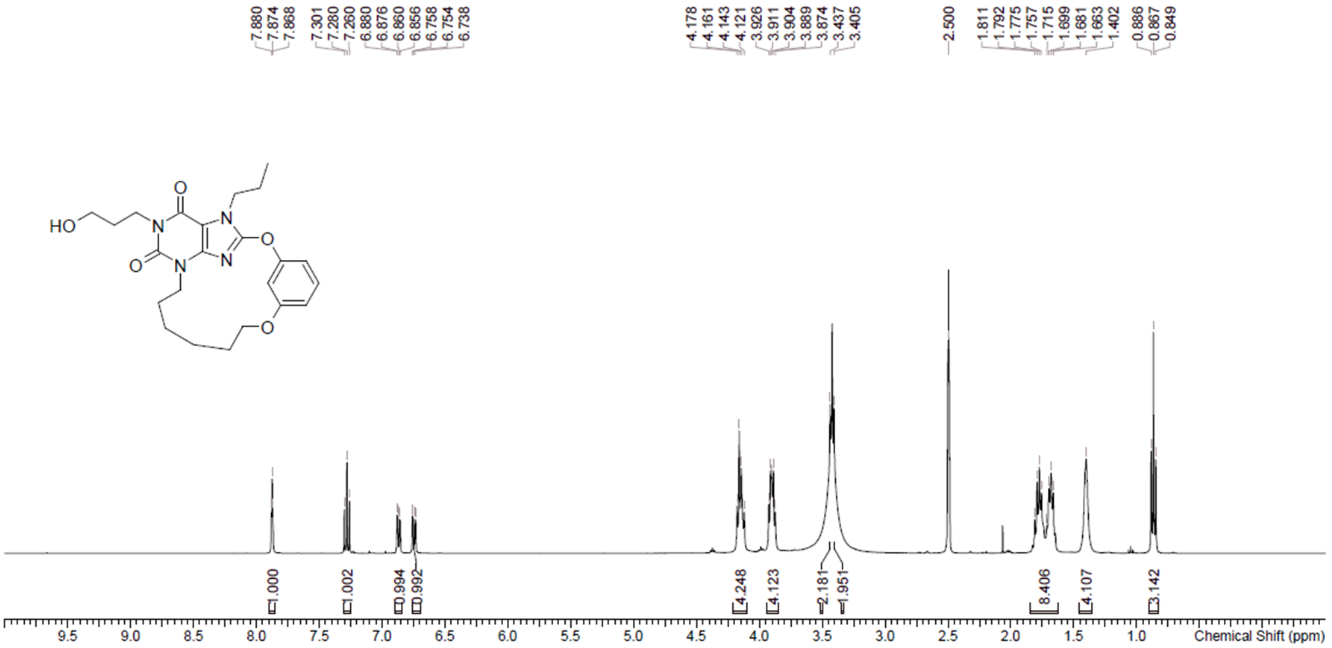

^{1}H NMR of compound **12**

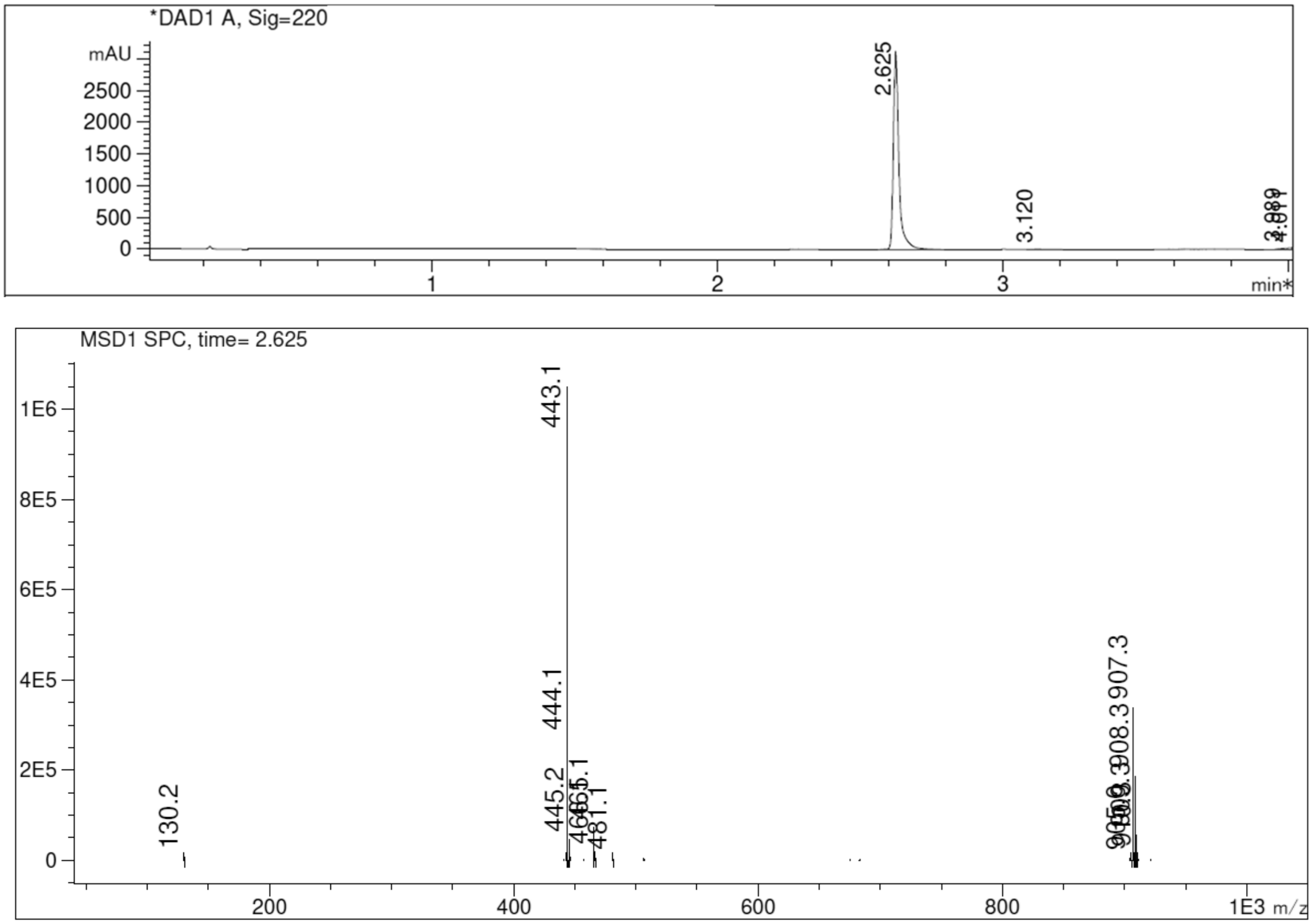

HPLC and MS of compound **12**

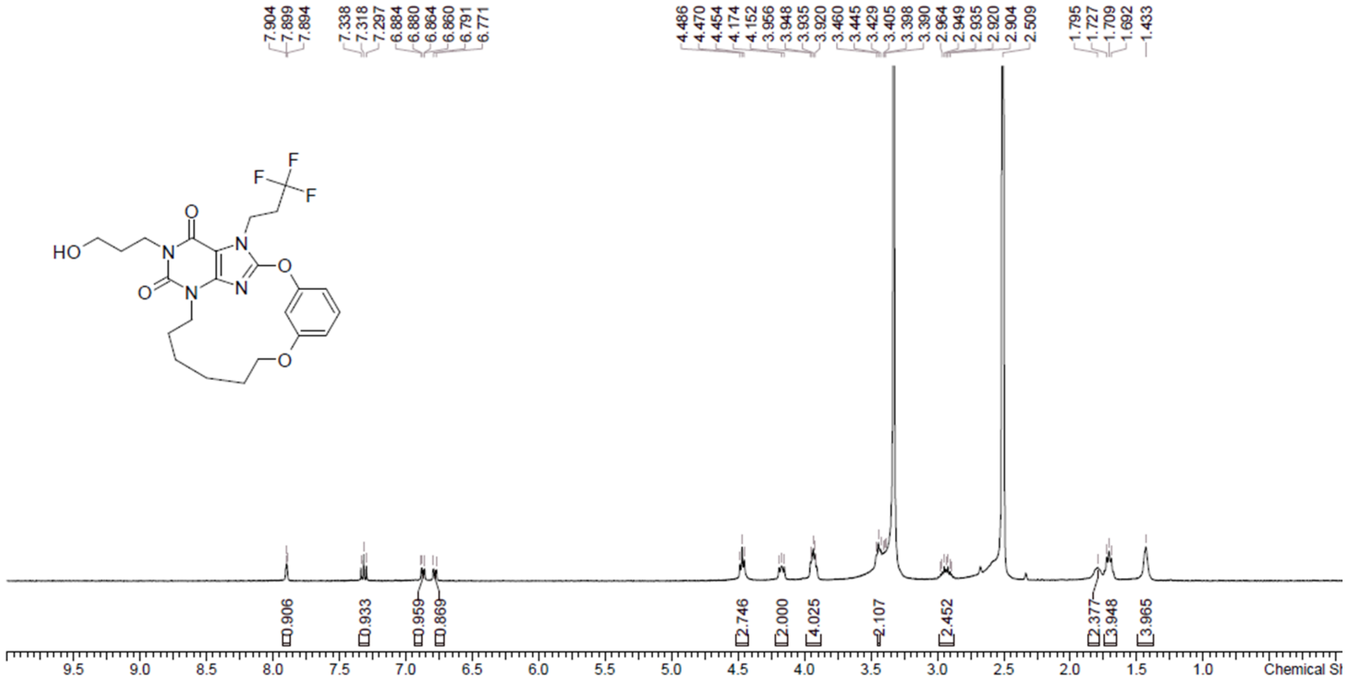

^{1}H NMR of compound **13**

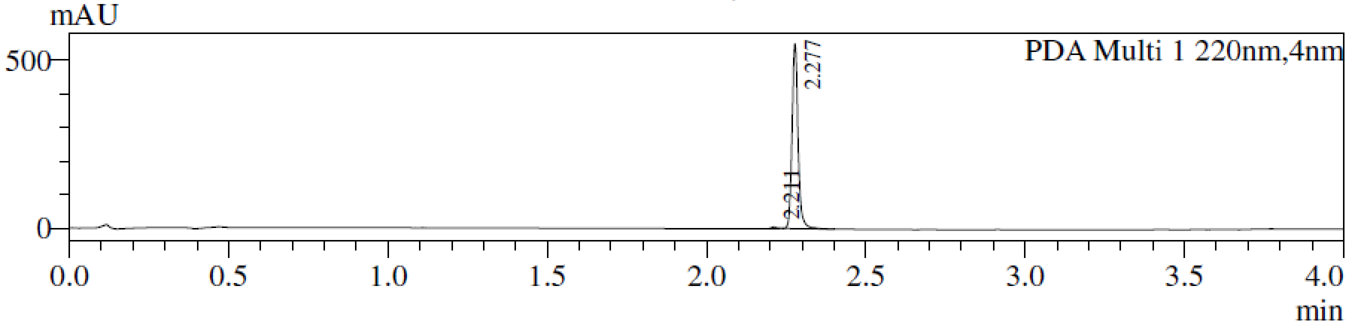

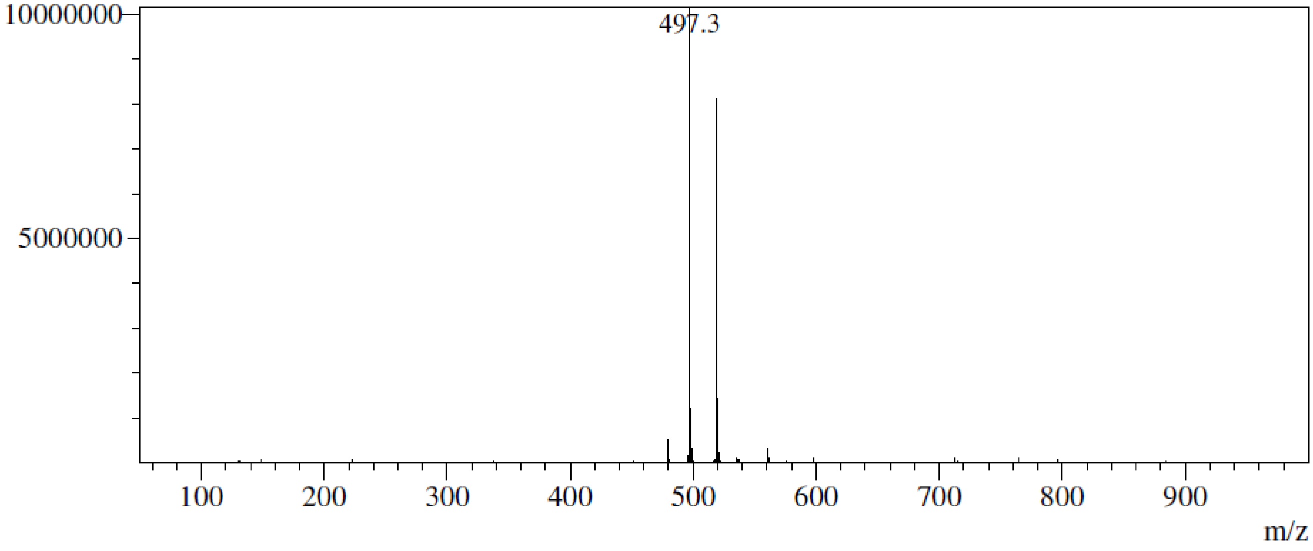

HPLC and MS of compound **13**

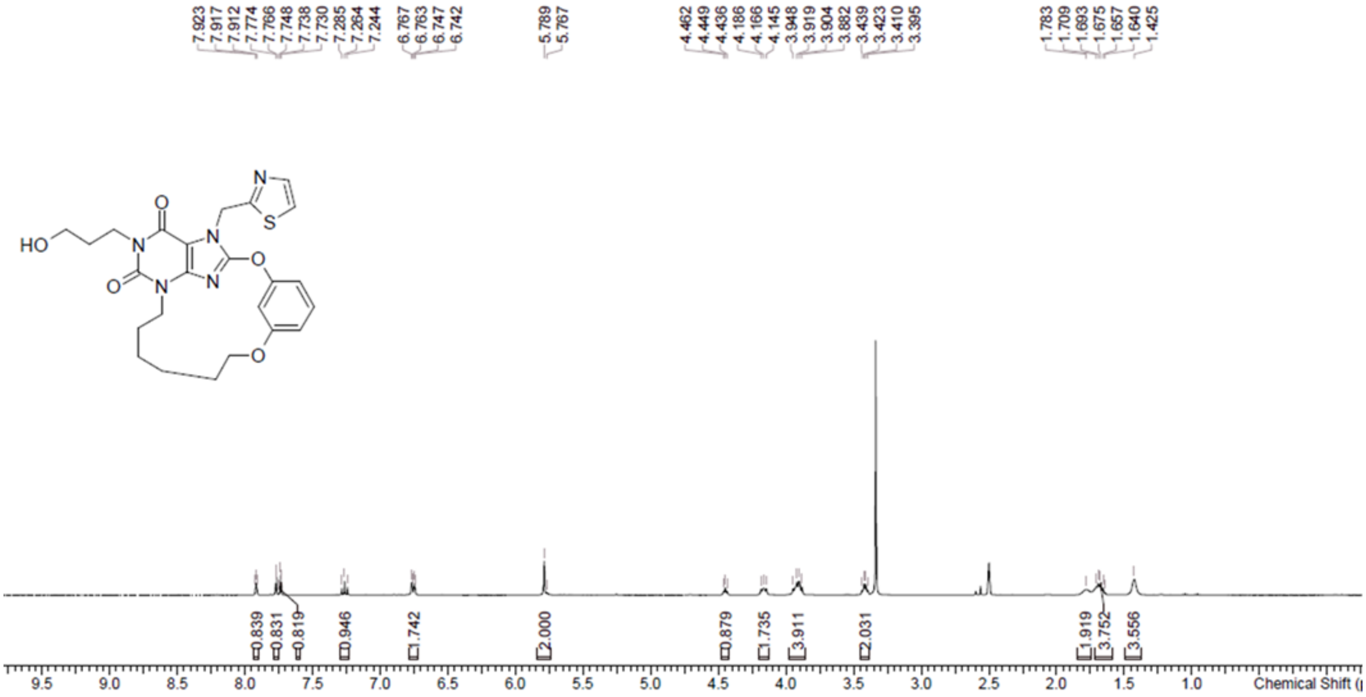

^{1}H NMR of compound **14**

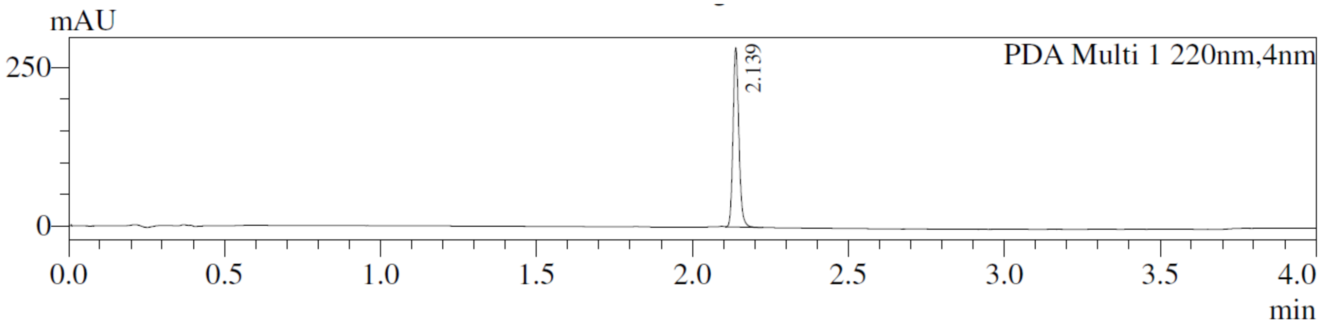

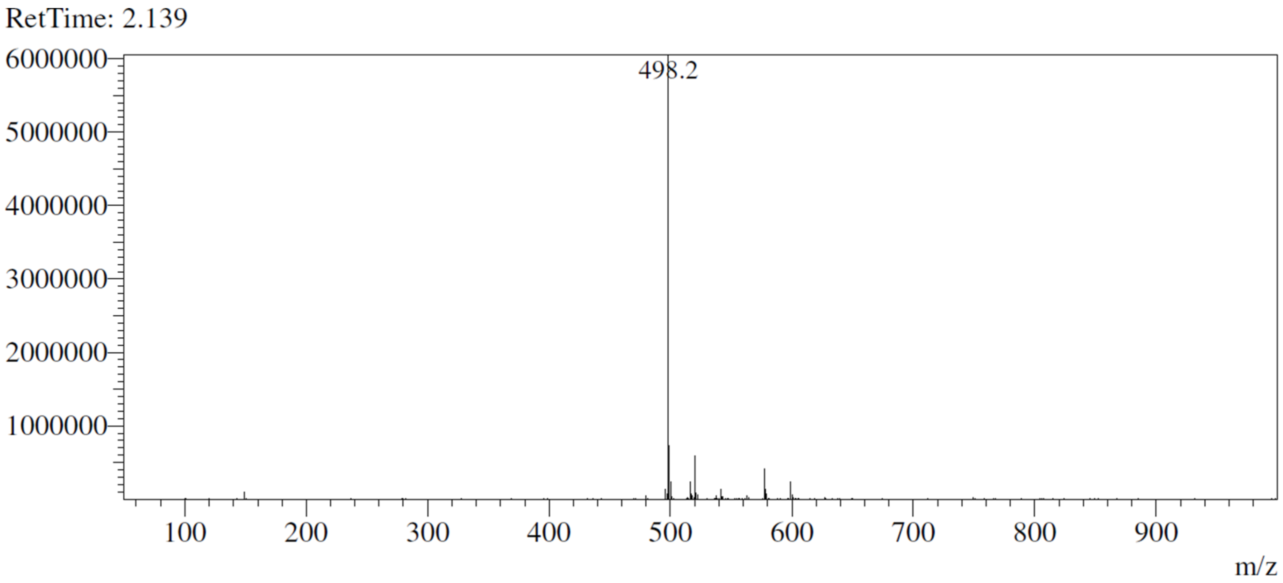

HPLC and MS of compound **14**

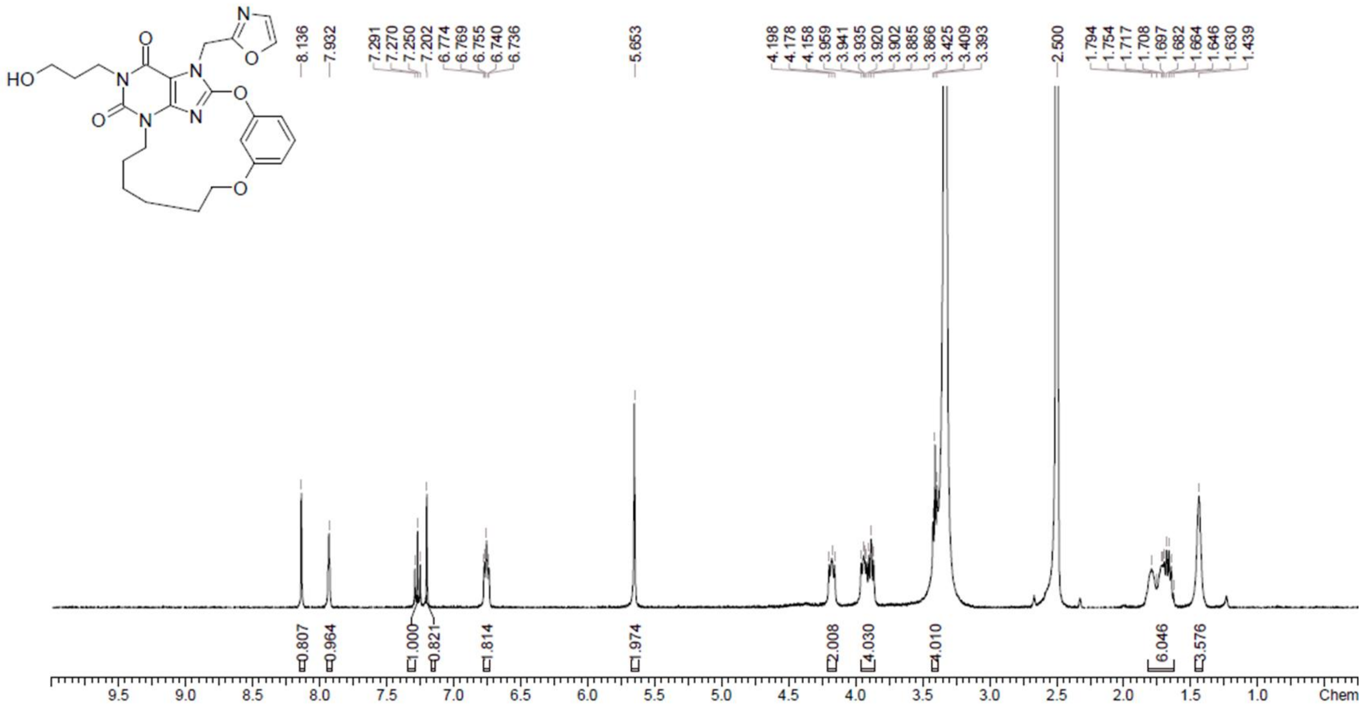

^{1}H NMR of compound **15**

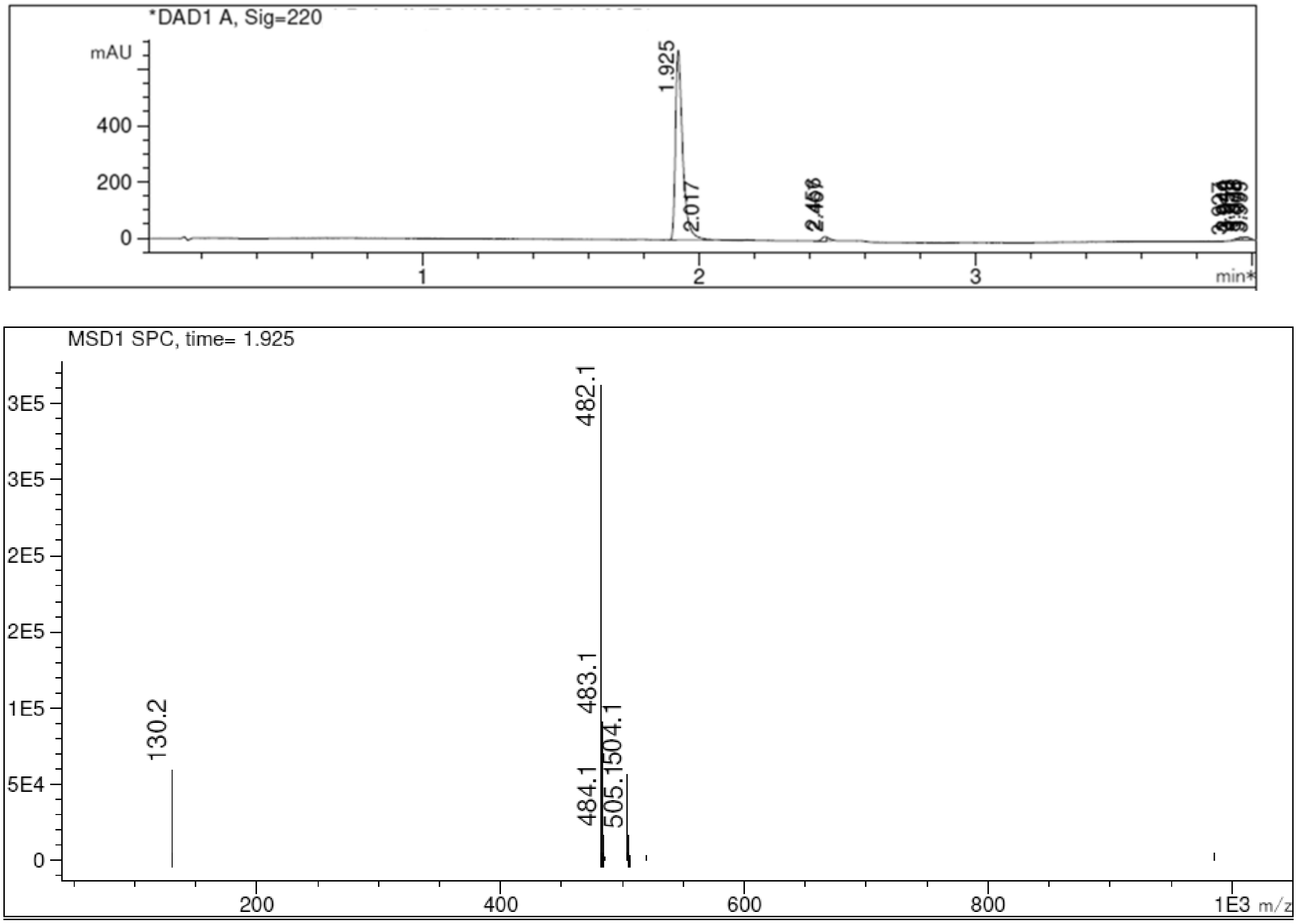

HPLC and MS of compound **15**

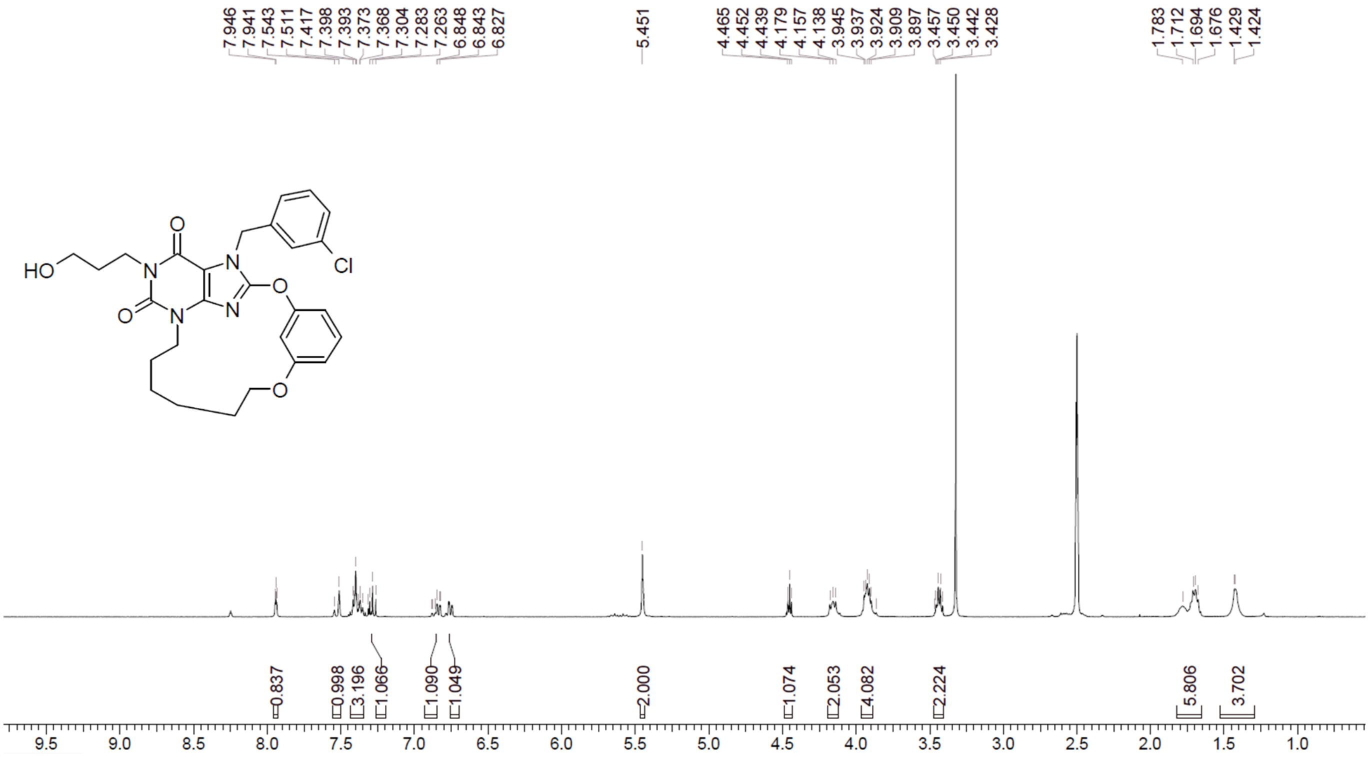

^{1}H NMR of compound **16**

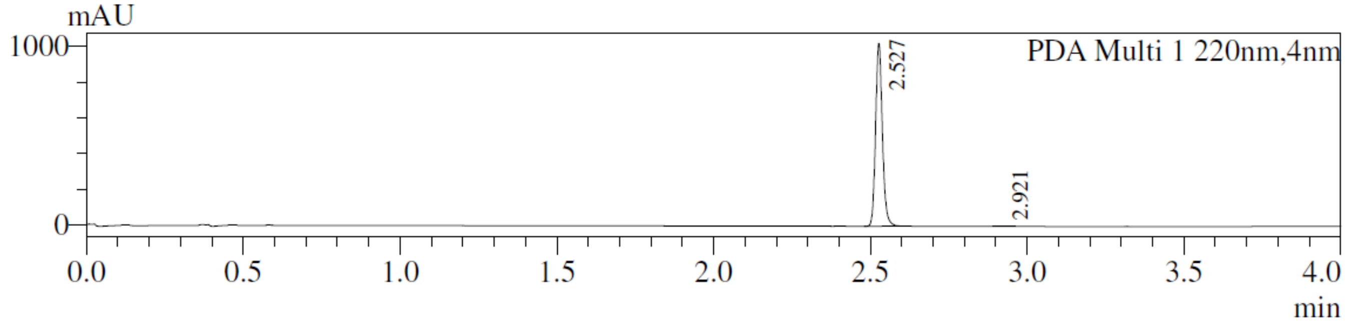

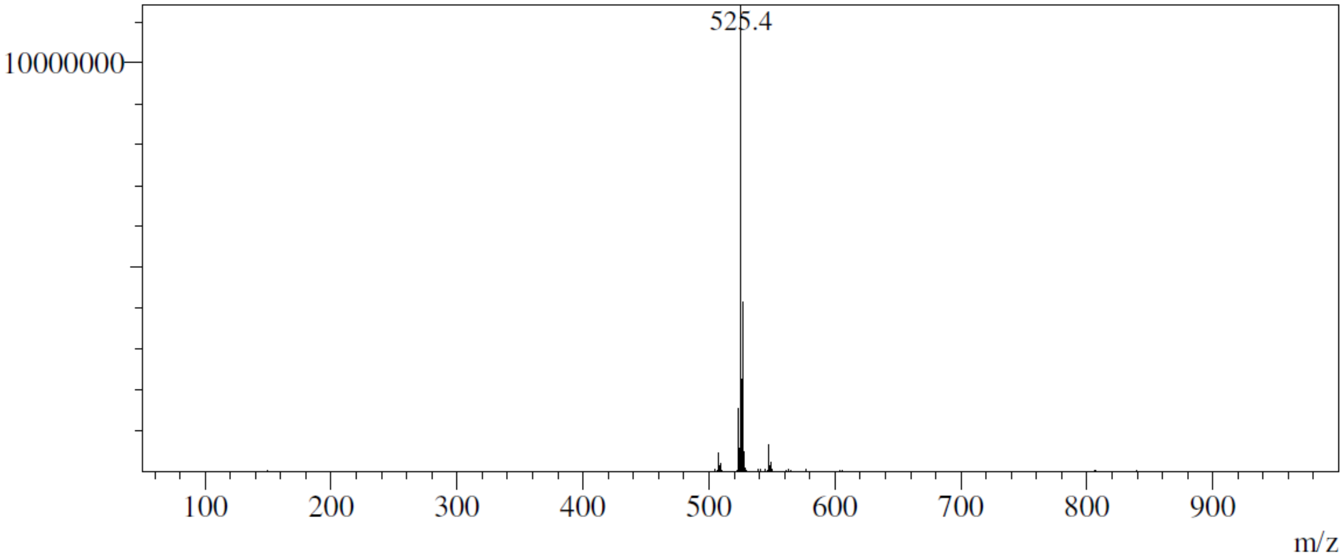

HPLC and MS of compound **16**

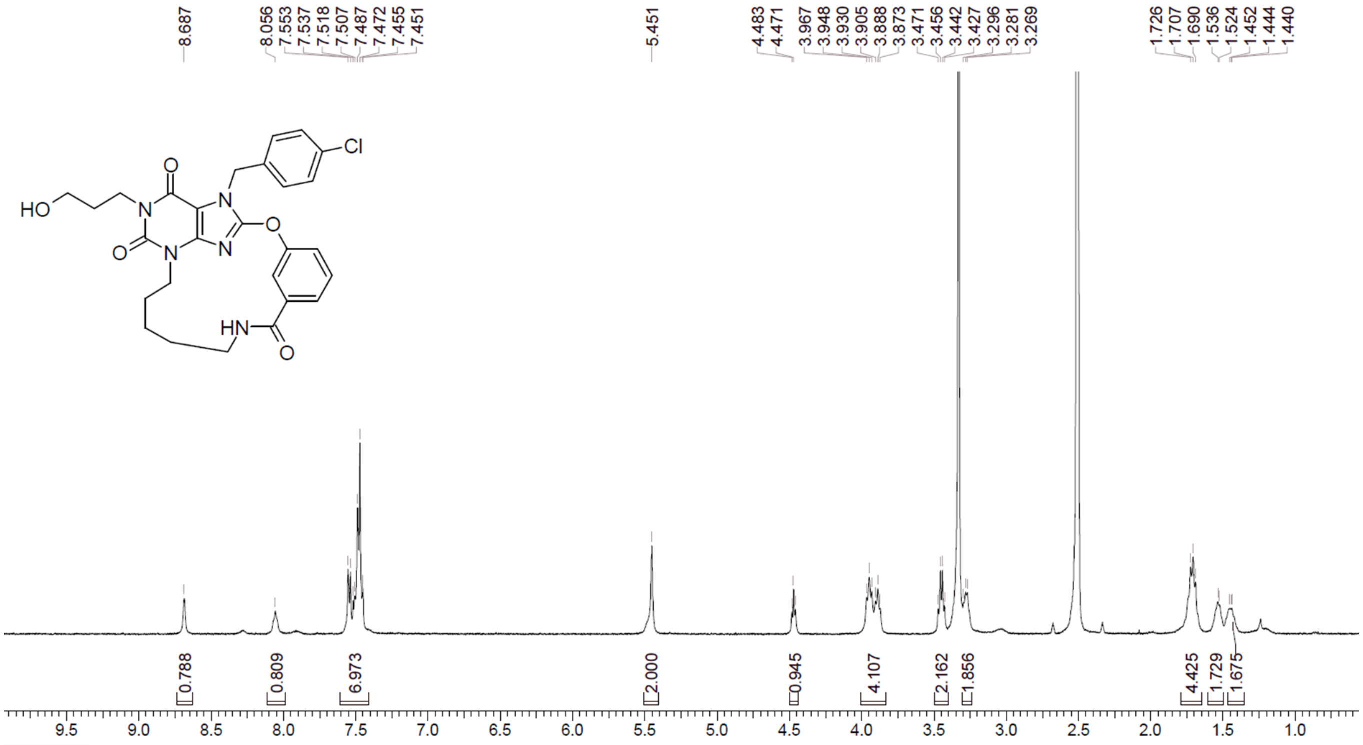

^{1}H NMR of compound **17**

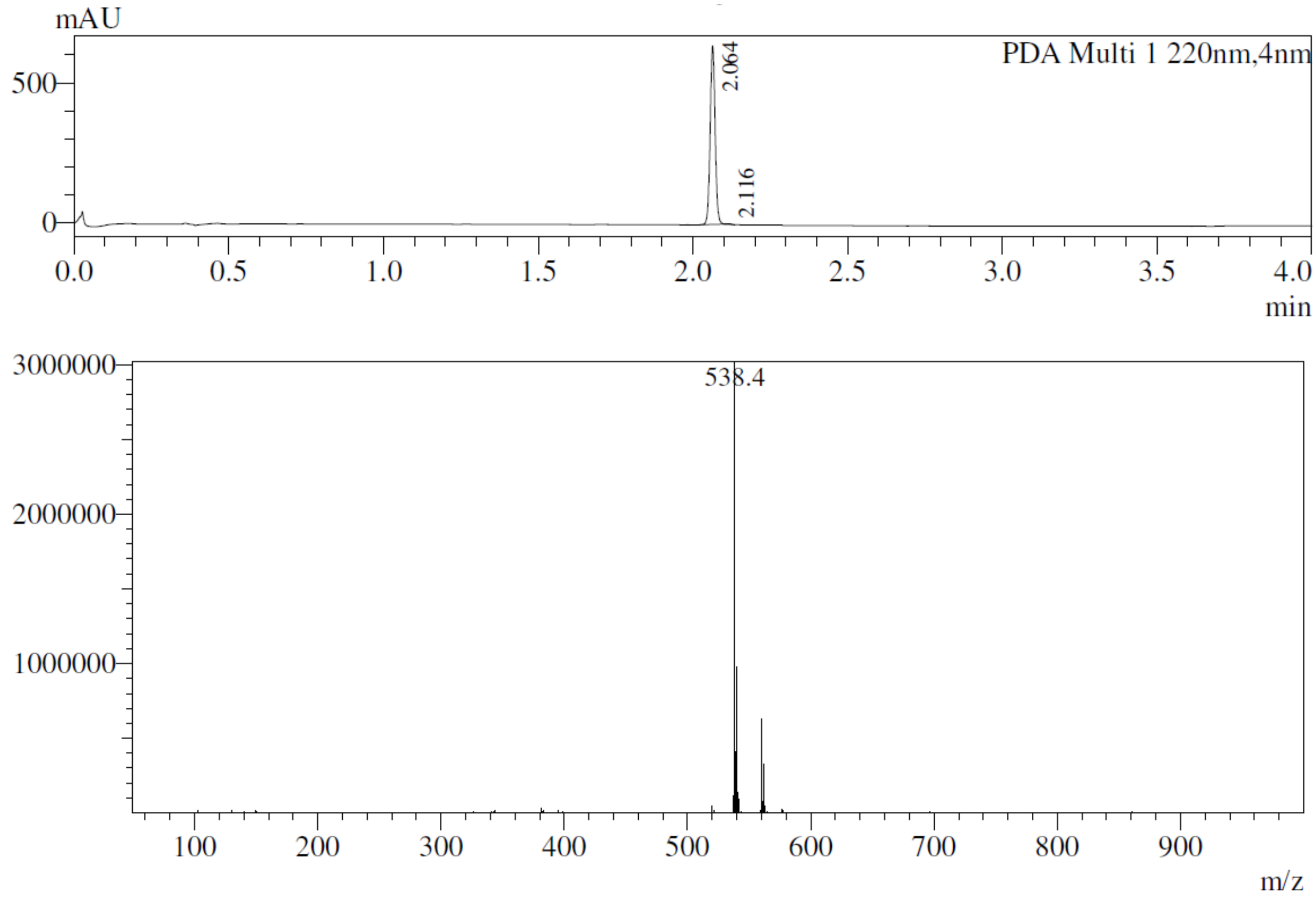

HPLC and MS of compound **17**

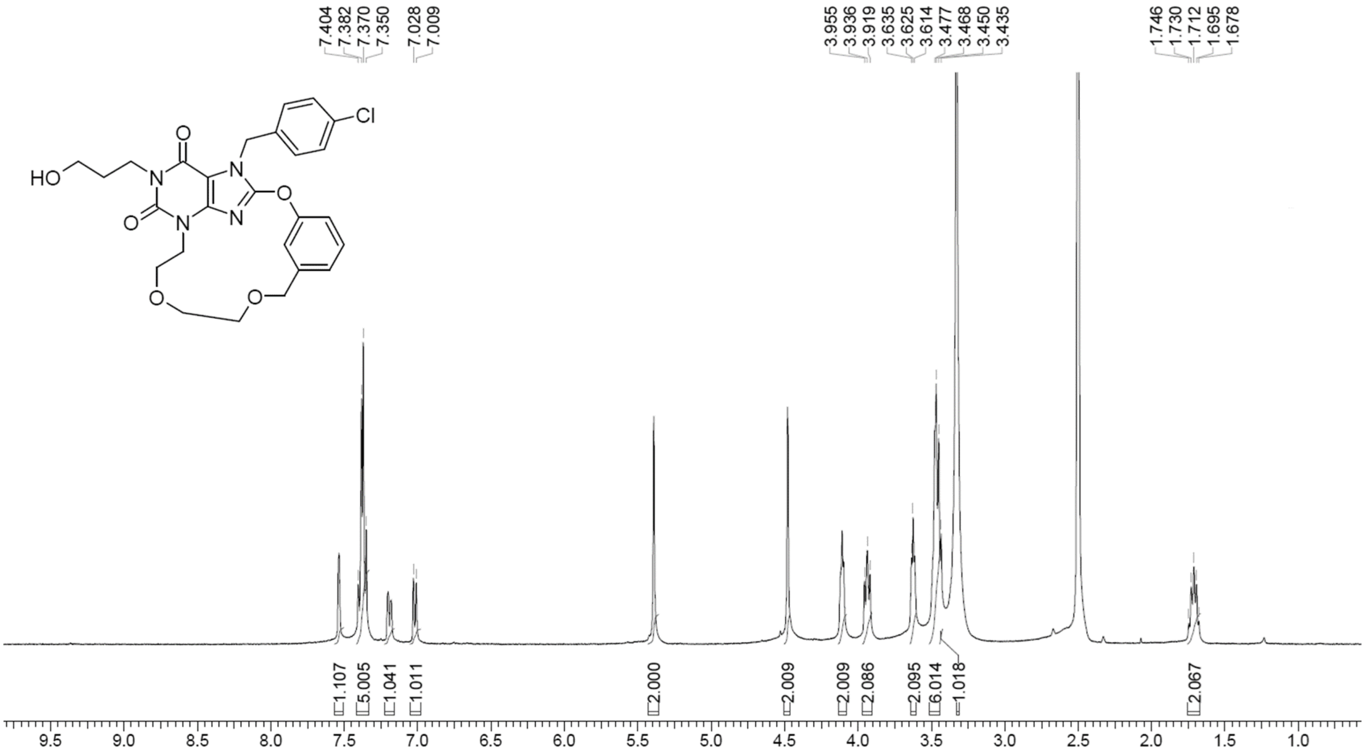

^{1}H NMR of compound **18**

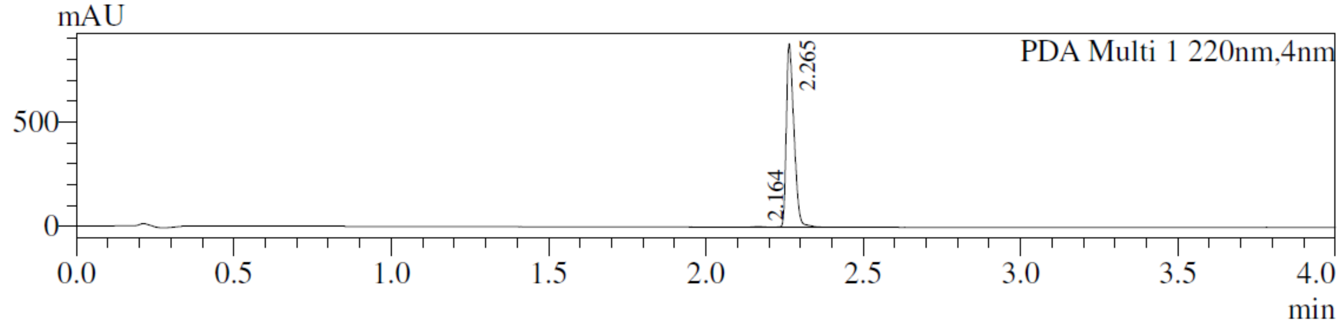

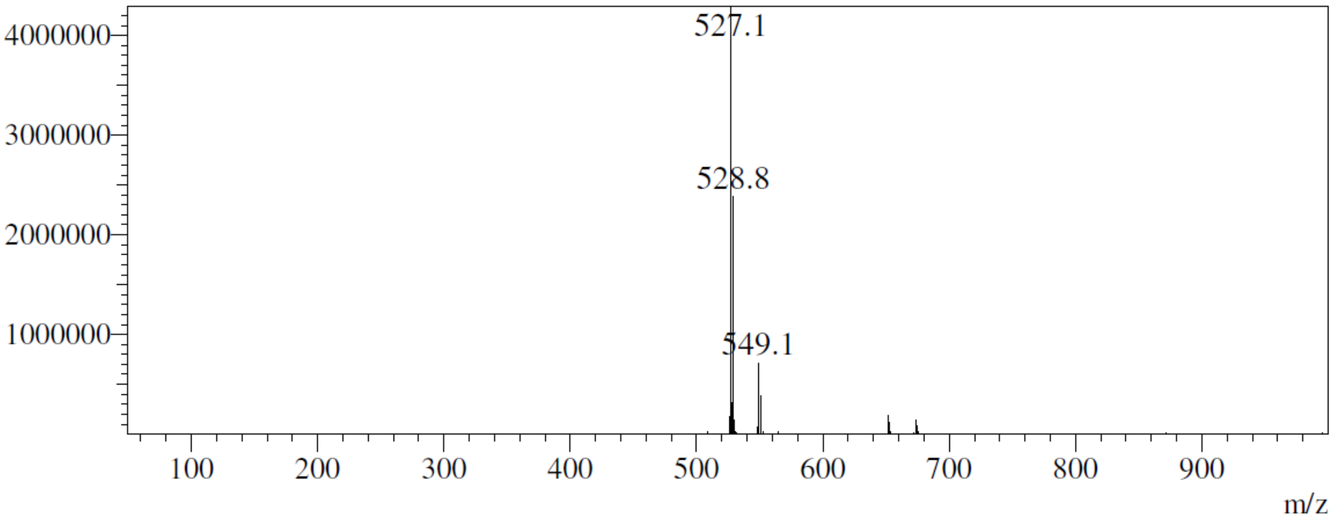

HPLC and MS of compound **18**

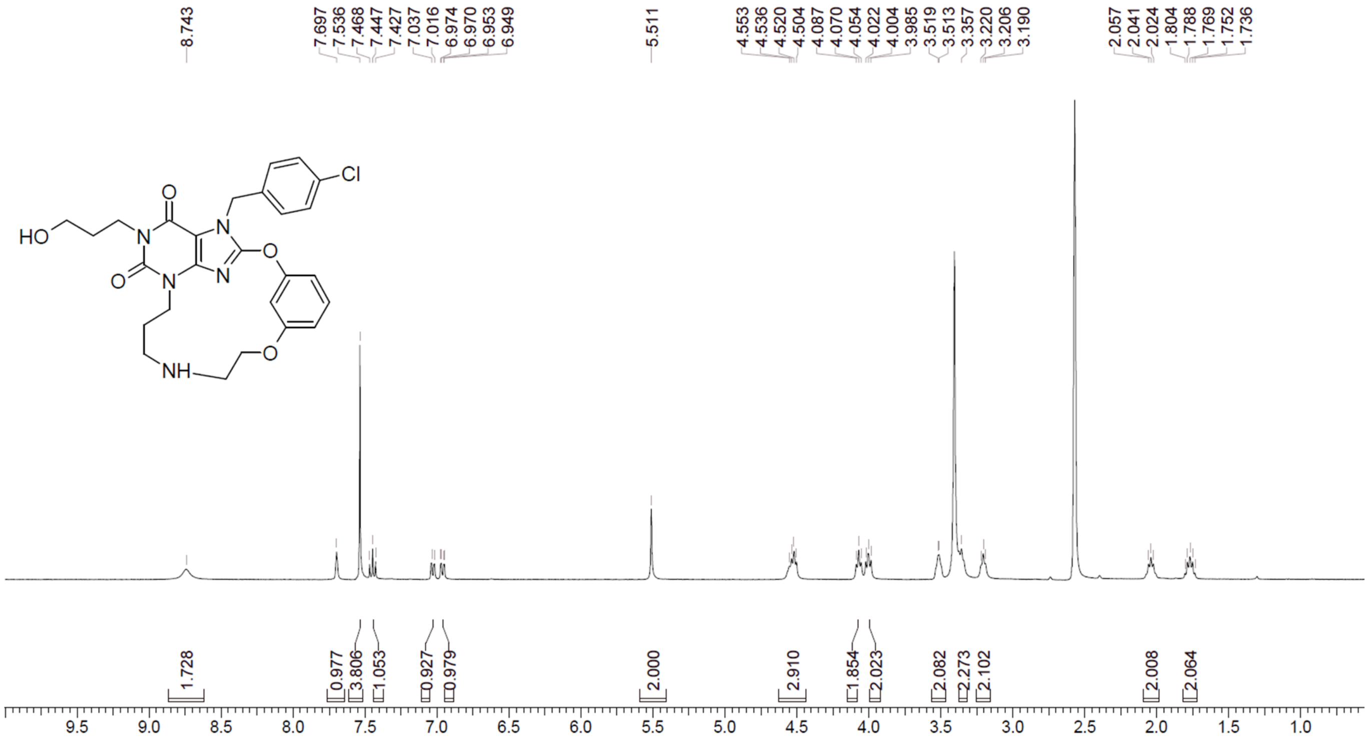

^{1}H NMR of compound **19**

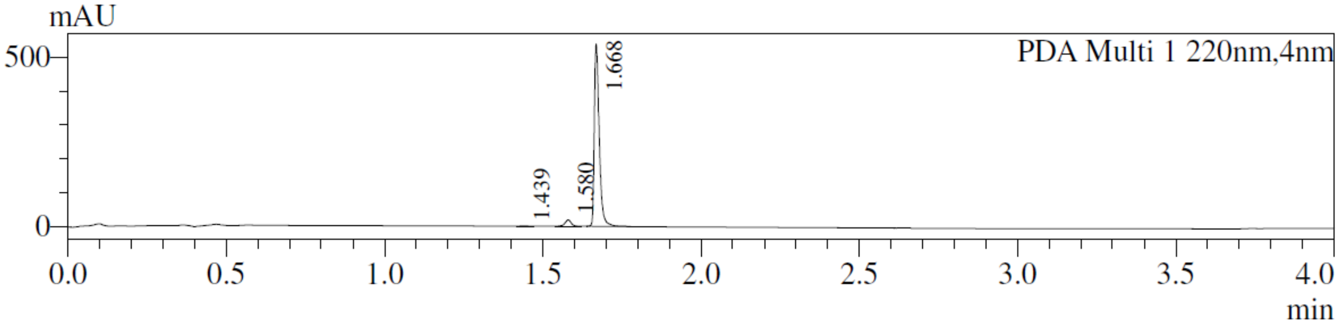

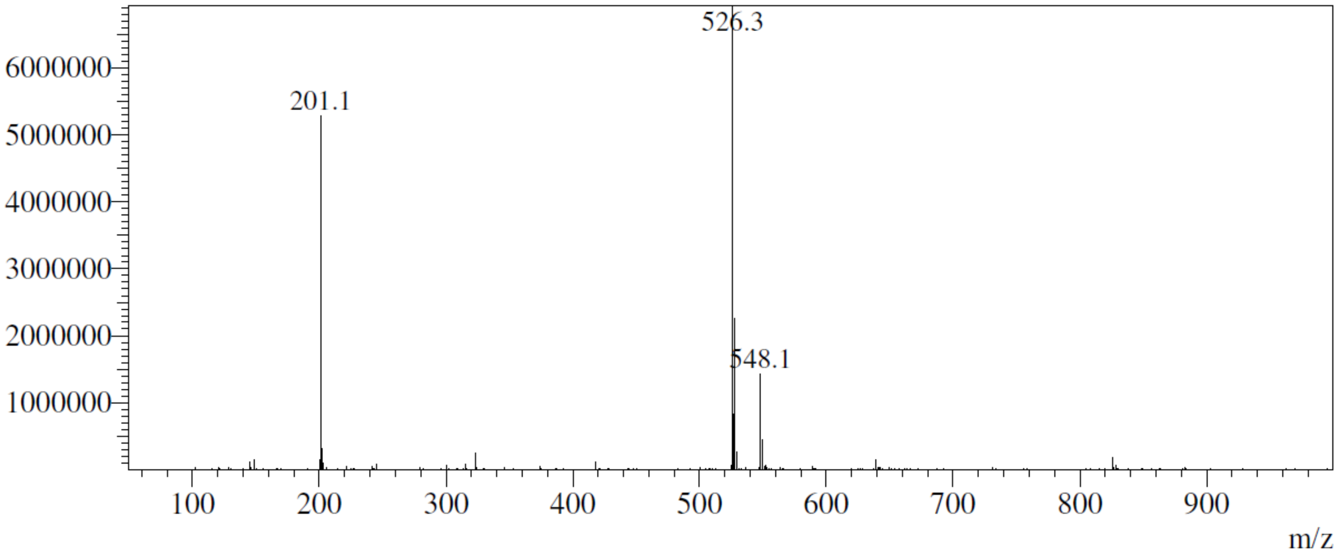

HPLC and MS of compound **19**

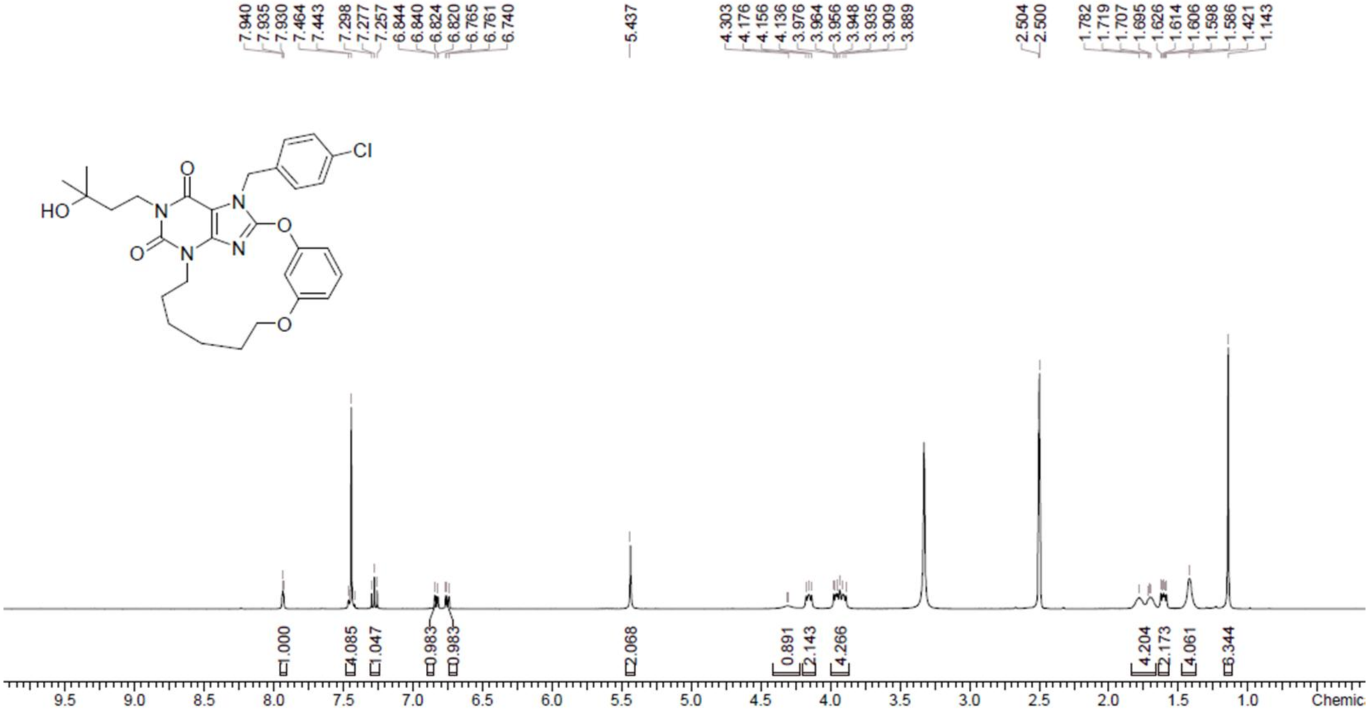

^{1}H NMR of compound **20**

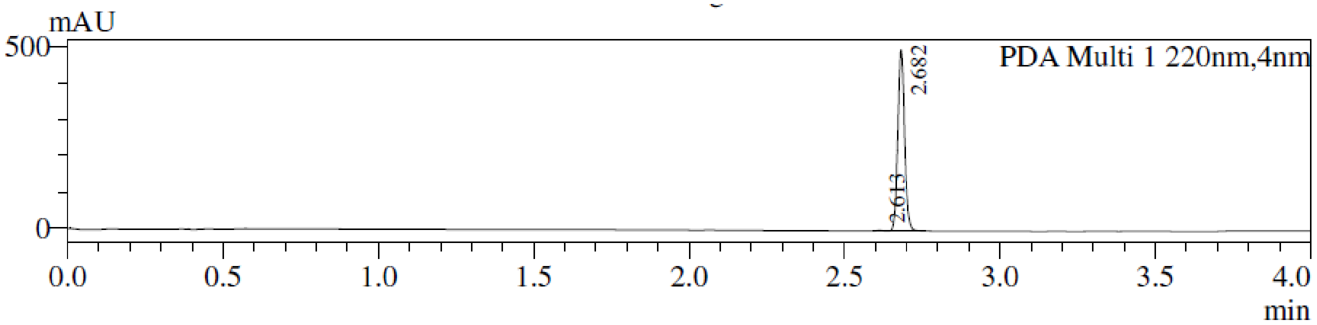

RetTime: 2.682

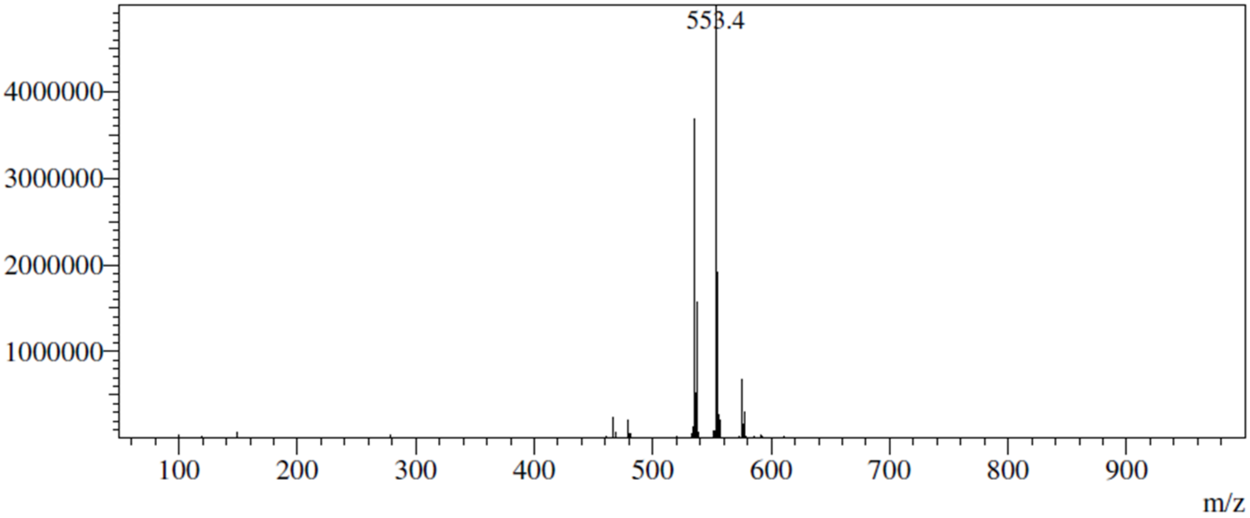

HPLC and MS of compound **20**

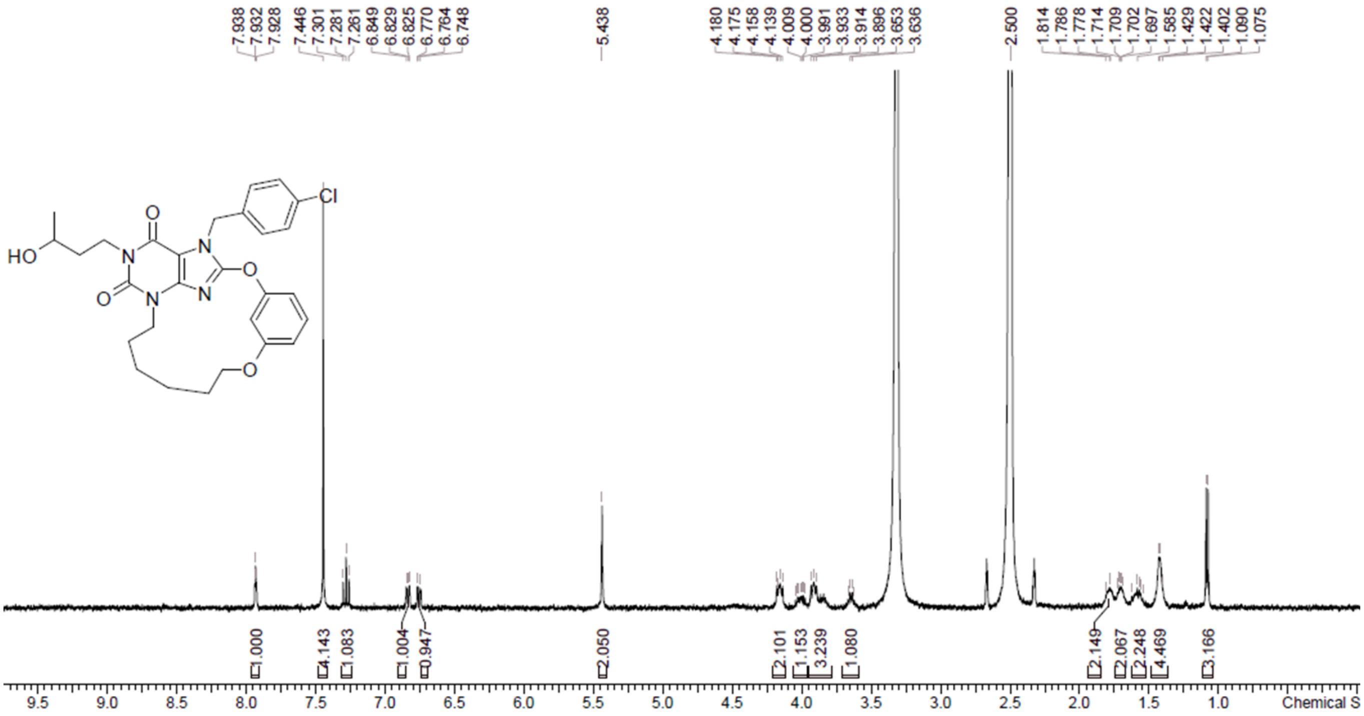

^{1}H NMR of compound **21**

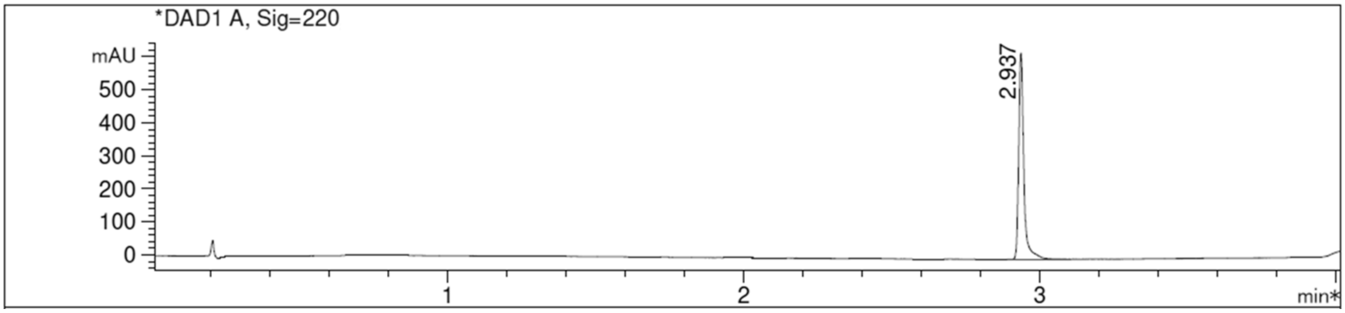

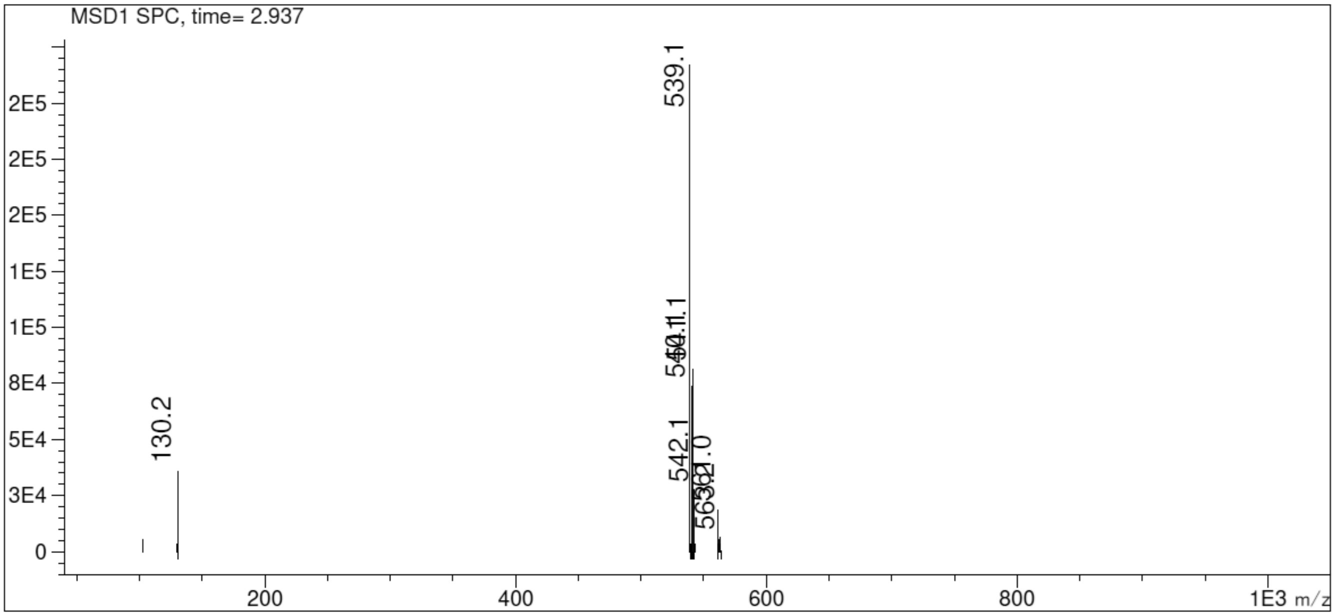

HPLC and MS of compound **21**

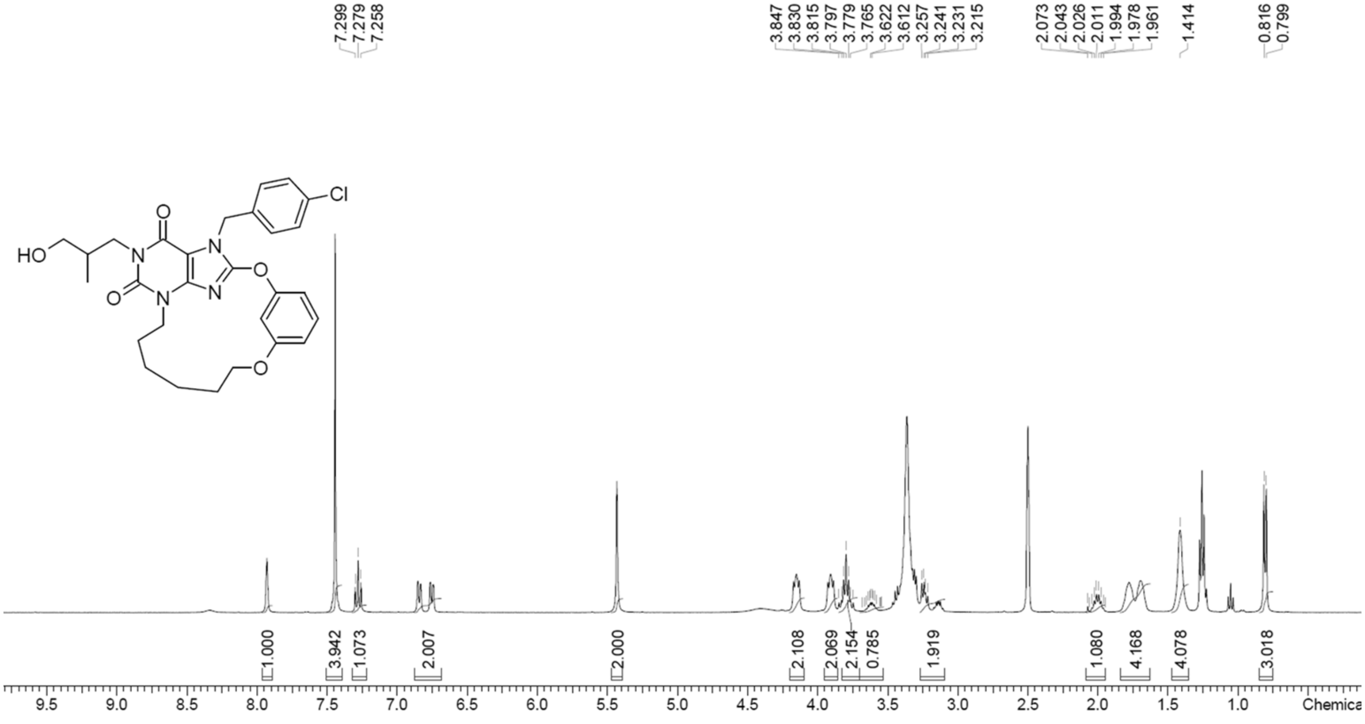

^{1}H NMR of compound **22**

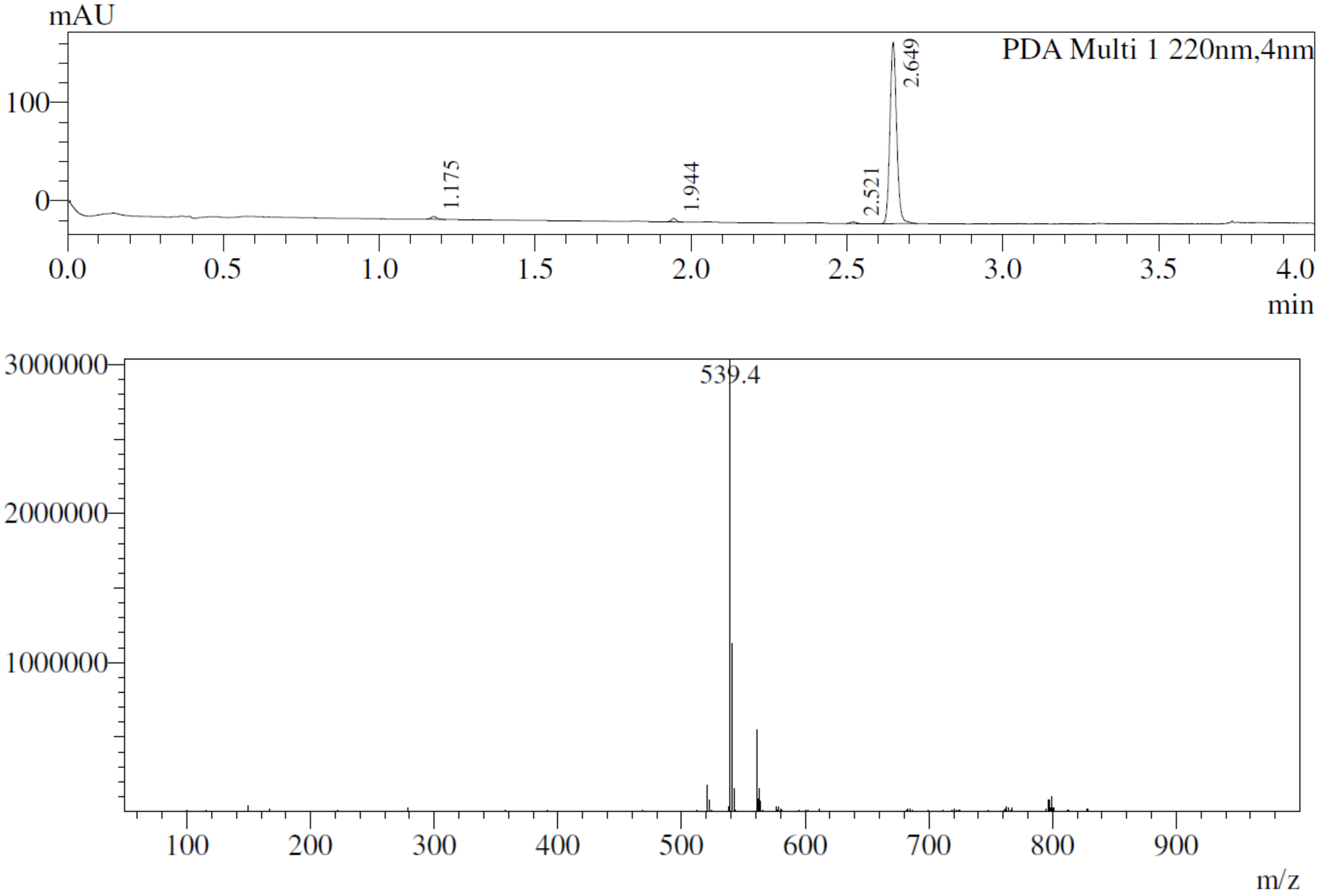

HPLC and MS of compound **22**

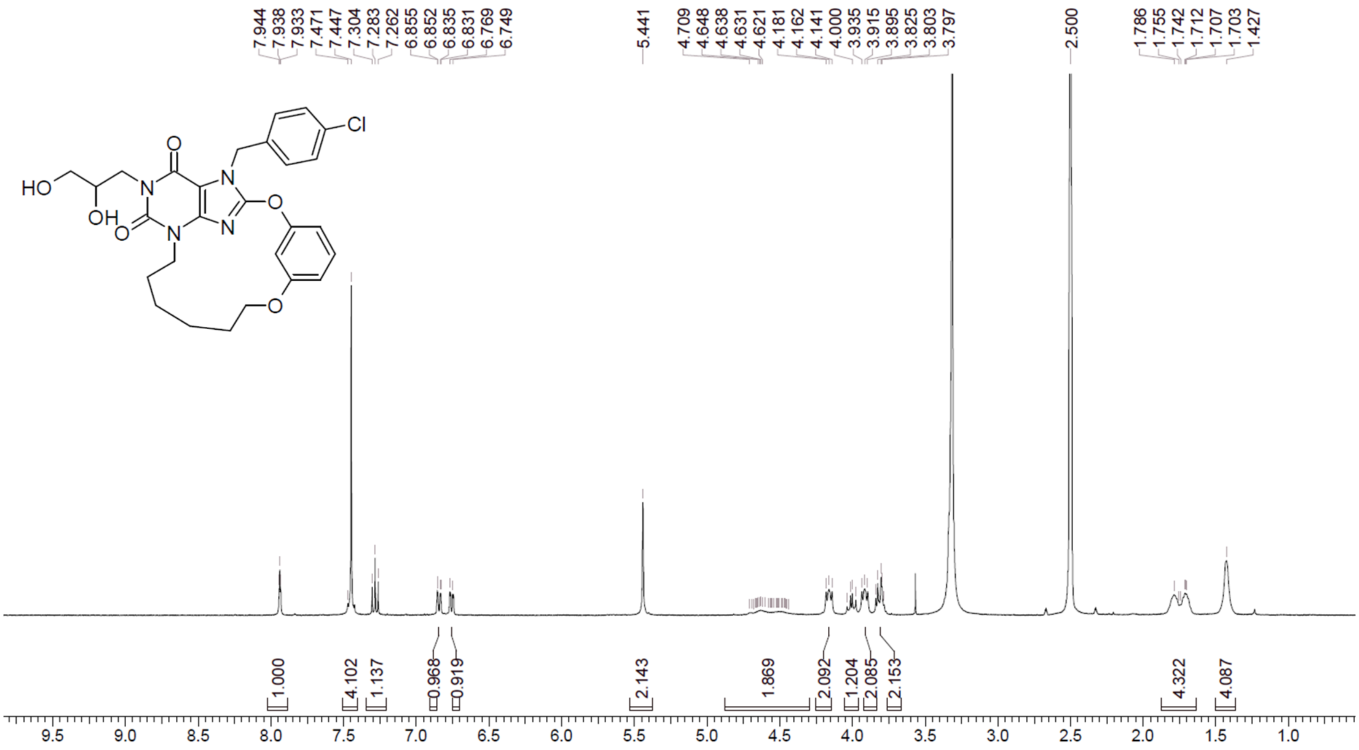

^{1}H NMR of compound **23**

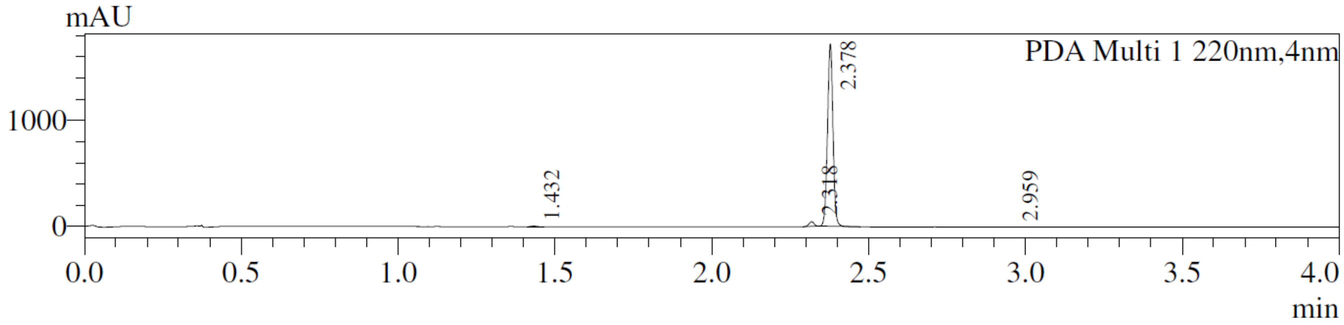

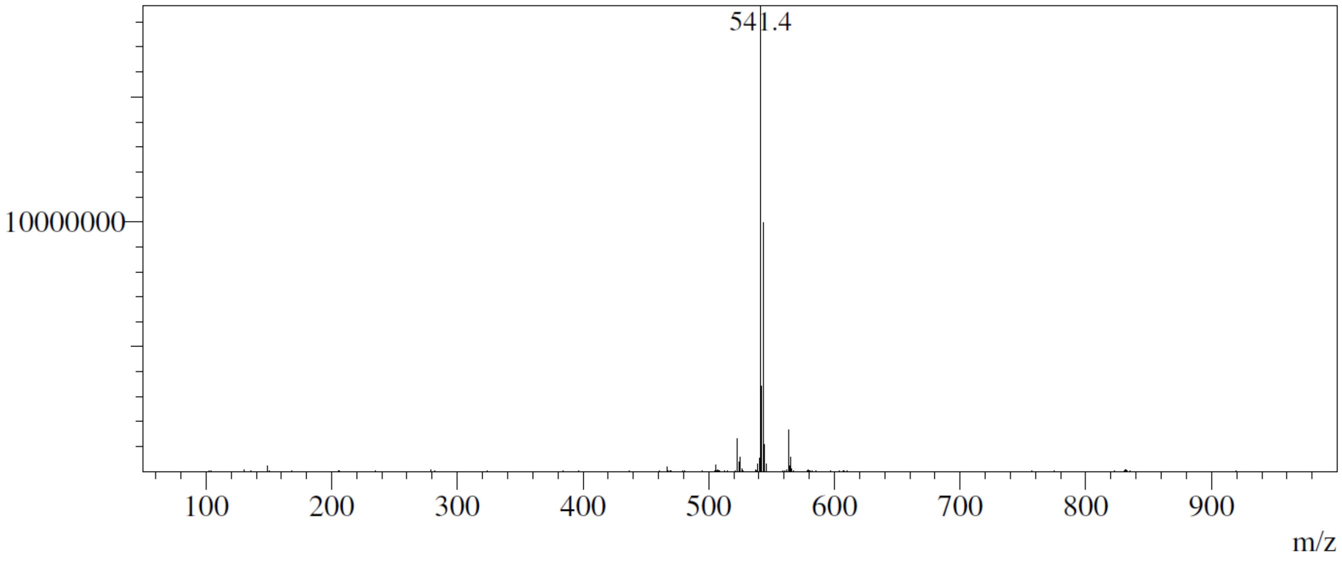

HPLC and MS of compound **23**

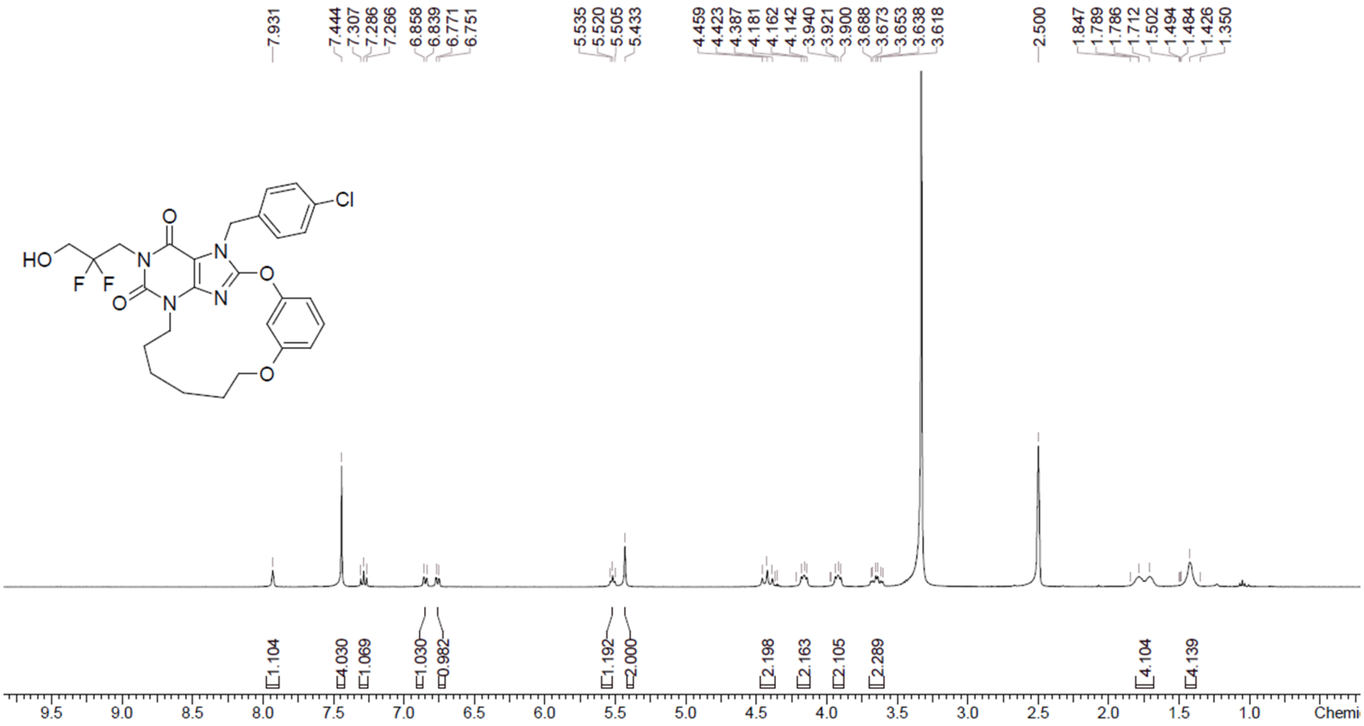

^{1}H NMR of compound **24**

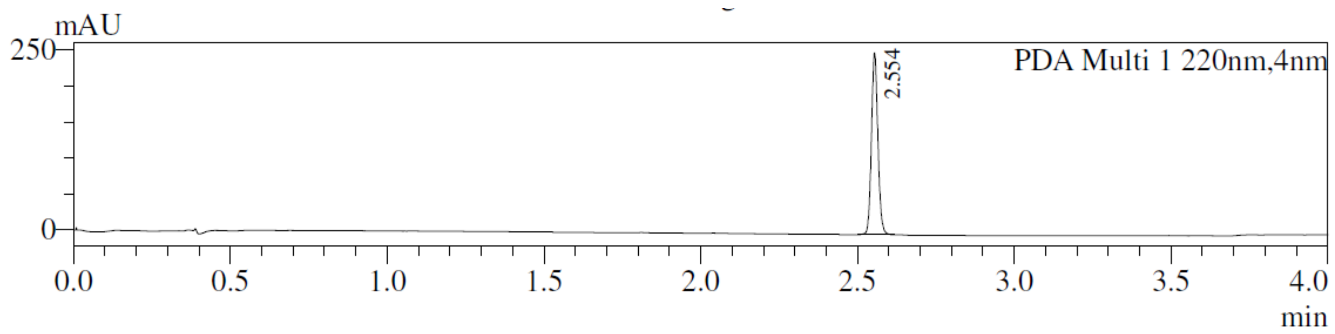

RetTime: 2.555

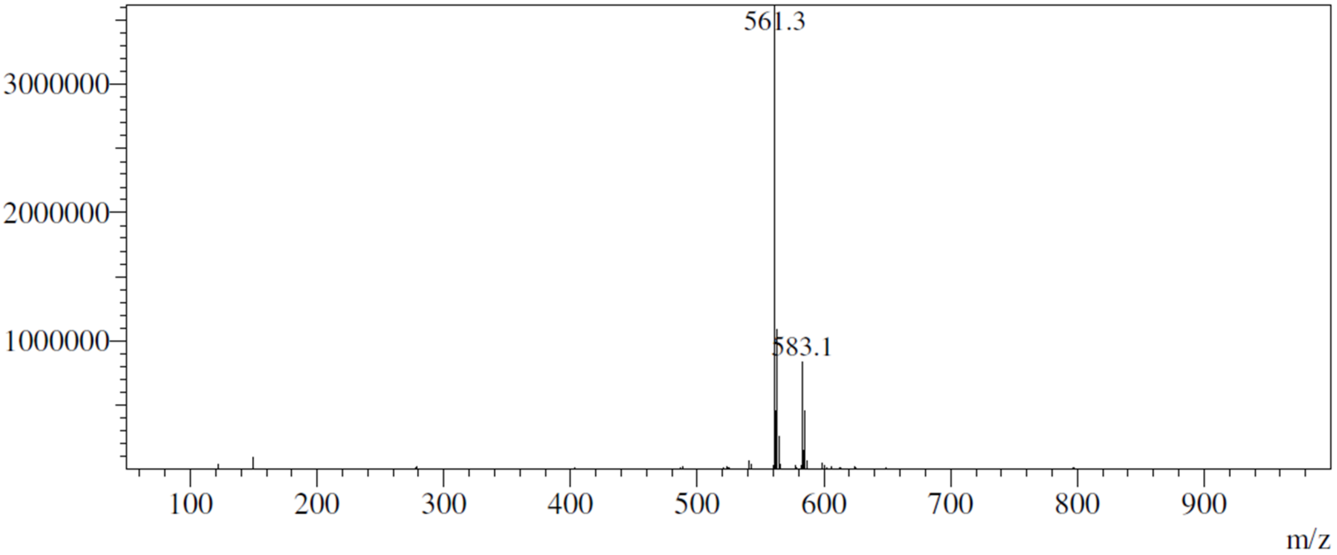

HPLC and MS of compound **24**
